# Design Platform for Sustainable Catalysis with Radicals: Electrochemical Activation of Cp_2_TiCl_2_ for Catalysis Unveiled

**DOI:** 10.1002/chem.202004519

**Published:** 2021-01-12

**Authors:** Tobias Hilche, Philip H. Reinsberg, Sven Klare, Theresa Liedtke, Luise Schäfer, Andreas Gansäuer

**Affiliations:** ^1^ Kekulé-Institut für Organische Chemie und Biochemie Universität Bonn Gerhard-Domagk-Straße 1 53121 Bonn Germany; ^2^ Institut für Physikalische und Theoretische Chemie, Universität Bonn Römerstraße 164 53117 Bonn Germany

**Keywords:** catalysis, density functional calculations, electrochemistry, radicals, screening

## Abstract

The combination of synthesis, rotating ring‐disk electrode (RRDE) and cyclic voltammetry (CV) measurements, and computational investigations with the aid of DFT methods shows how a thiourea, a squaramide, and a bissulfonamide as additives affect the E_q_C_r_ equilibrium of Cp_2_TiCl_2_. We have, for the first time, provided quantitative data for the E_q_C_r_ equilibrium and have determined the stoichiometry of adduct formation of [Cp_2_Ti(III)Cl_2_]^−^, [Cp_2_Ti(III)Cl] and [Cp_2_Ti(IV)Cl_2_] and the additives. By studying the structures of the complexes formed by DFT methods, we have established the Gibbs energies and enthalpies of complex formation as well as the adduct structures. The results not only demonstrate the correctness of our use of the E_q_C_r_ equilibrium as predictor for sustainable catalysis. They are also a design platform for the development of novel additives in particular for enantioselective catalysis.

## Graphical abstract

**What mechanism‐based screening for titanocene catalysis is all about**! Combining insights from synthesis and theory with cyclic voltammetry and the rotating ring disk electrode is the deal for understanding not only how but also why condition screening for the titanocene catalyzed radical arylation works.

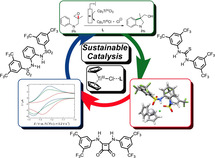

## Introduction

**Scheme 1:**
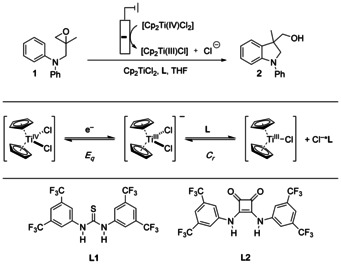
Radical arylation after electrochemical activation (top) and E_q_C_r_ equilibrium of Cp_2_TiCl_2_ (middle). **L** denotes anion receptors allowing a shift of the E_q_C_r_ equilibrium by reversible cleaving of Cl^−^ through hydrogen bonding.

Catalysis has shaped our modern society, providing functional molecules, materials, or processes both in the lab and on industrial scale. Substituting stoichiometric reactions by catalytic alternatives offers a conceptionally straightforward approach to a more sustainable, green chemistry.^[1]^ However, as simple this idea may appear in principle, identifying the optimal catalytic conditions usually demands laborious experimental reaction screening through expensive use of workforce, material and laboratory time. To reinforce the sustainability claim of catalysis, we propose to identify mechanistic key aspects or so‐called “predictors” before setting up any reaction, avoiding both the *tour de force* and even the designed experimental investigation of the overall catalytic reaction. With these mechanistic key features at hand, we can then predict the optimal catalytic reaction condition based on scientific arguments rather than our usual into the blue approach, so that ideally only a few experimental combinations need to be verified. These “predictors” require either the knowledge of the overall mechanism or at least an understanding of the essentials of the catalytic cycle. We chose to illustrate our “predictor” concept with the optimization of electrochemical epoxide arylation. It is only after we exploit widely used and readily available cyclic voltammetry (CV) and DFT techniques to identify the “predictors” of this reaction that we enter the synthesis lab to try our smart guess. For catalysis in single electron steps^[2]^ or metalloradical catalysis,^[3]^ cyclic voltammetry (CV) is particularly an ideal screening technique because the metals shuttle between neighboring oxidation states. Here, we demonstrate that the E_q_C_r_ equilibrium^[4]^ is a predictor for the performance of the electrochemical, titanocene catalyzed radical epoxide arylation, providing understanding of the catalyst formation by bulk electrolysis of precatalyst Cp_2_TiCl_2_ in THF (Scheme 1).^[5]^ For a successful electrochemical reduction of [Cp_2_Ti(IV)Cl_2_] the initial product of electron transfer [Cp_2_Ti(III)Cl_2_]^−^ at the electrode must be transformed into arylation catalyst [Cp_2_Ti(III)Cl], that is—in contrast to [Cp_2_Ti(III)Cl_2_]^−^—easily soluble in THF and can diffuse from the electrode. Increasing the concentration of [Cp_2_Ti(III)Cl] by halide abstraction from [Cp_2_Ti(III)Cl_2_]^−^ will therefore facilitate bulk electrolysis of [Cp_2_Ti(IV)Cl_2_]. While [Cp_2_Ti(III)Cl] constitutes the active catalyst for epoxide arylation, [Cp_2_Ti(III)Cl_2_]^−^ provides a catalytic resting state, that prevents undesired catalyst decomposition.^[6c]^

Adjusting the relative amounts of both species in a reversible way thus allows a fine‐tuning of the performance of the catalytic system. It turned out that the E_q_C_r_ equilibrium and therefore bulk electrolysis of [Cp_2_Ti(IV)Cl_2_] in THF can be controlled by the addition of supramolecular additives, that bind halides through hydrogen bonding.^[7]^ We show then that the control of the E_q_C_r_ equilibrium directly translates into the synthetic performance of the catalytic radical arylation^[6]^ in THF.

Here, we demonstrate that our qualitative analysis of the cyclic voltammograms (CVs) of the E_q_C_r_ equilibrium as predictor for the radical arylation is applicable to bissulfonamides as another class of supramolecular additives. Moreover, it is in accord with quantitative measurements at the rotating ring‐disk electrode (RRDE).^[8]^ The combination of the experimental results with the DFT studies of the intermediates of the E_q_C_r_ equilibrium delivers a complete mechanistic analysis of our predictor. Our study not only leads to a better understanding of the systems already investigated. It also delivers a design platform for the use of supramolecular halide binders in enantioselective catalysis and for their use with complexes of metals other than titanium.

## Results and Discussion

### Cyclic voltammetry and radical arylation

A critical aspect for efficient screening is a fast and cost‐efficient technique for a conclusive description of the investigated system. In our case, CV^[9]^ is an ideal tool because the E_q_C_r_ equilibrium includes an electron transfer step and involves species with different redox‐potentials. CV not only allows a characterization of the components of a solution. Relevant data can be obtained in short periods of time and with minor amounts of material (0.02 mmol).

**Figure 1:**
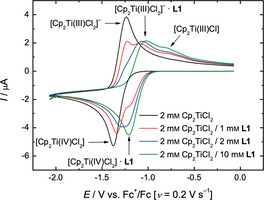
CVs of 2 mm Cp_2_TiCl_2_ and **L1** in 0.2 m Bu_4_NPF_6_/THF at 0.2 Vs^−1^.

**Figure 2:**
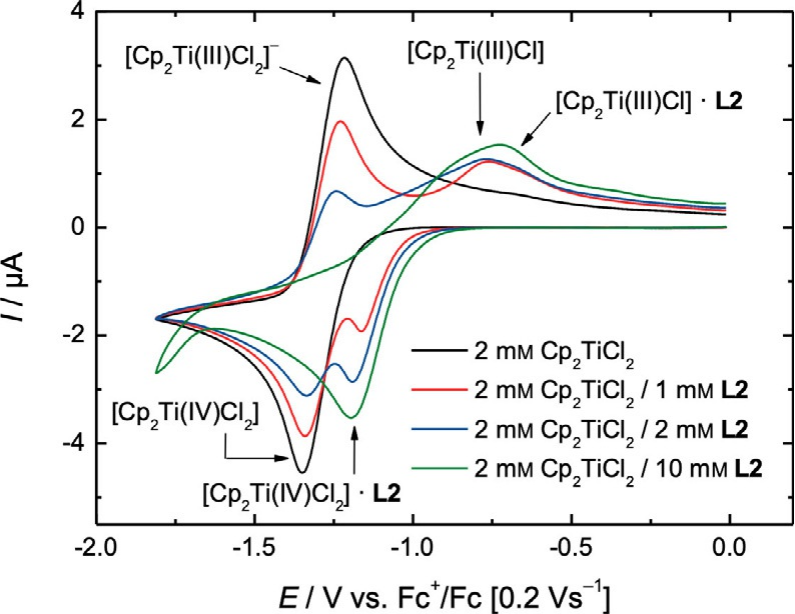
CVs of 2 mm Cp_2_TiCl_2_ and **L2** in 0.2 m Bu_4_NPF_6_/THF at 0.2 Vs^−1^.

Our previous studies showed that thiourea **L1** ^[10]^ and squaramide **L2** ^[11]^ (Scheme 1) are suitable additives for the bulk electrolysis of [Cp_2_Ti(IV)Cl_2_] in THF and the use of the resulting solutions for radical arylation. The CVs (**L1**: Figure 1; **L2**: Figure 2) highlight the reasons for this assessment. Compared to the CV of [Cp_2_Ti(IV)Cl_2_] without additives (black traces), the oxidation waves pertaining to [Cp_2_Ti(III)Cl_2_]^−^ have a reduced intensity and that of [Cp_2_Ti(III)Cl] an increased intensity. This effect is significantly more pronounced for **L2** than for **L1**. We assumed that these effects predict a successful bulk electrolysis and catalytic radical arylation.

**Scheme 2:**
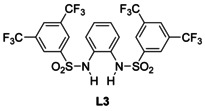
H‐bonding anion receptor **L3** investigated as additive to amplify the E_q_C_r_ mechanism of Cp_2_TiCl_2_.

**Figure 3:**
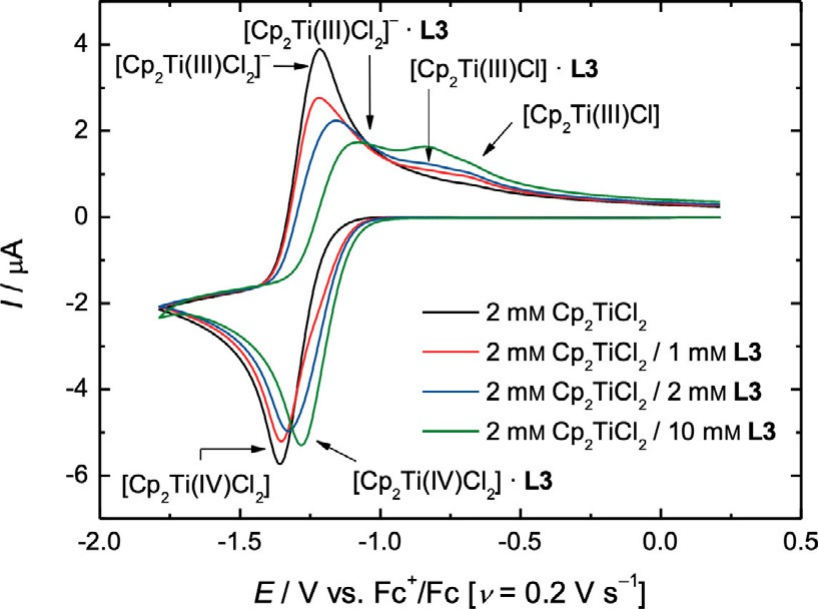
CVs of 2 mm Cp_2_TiCl_2_ and **L3** in 0.2 m Bu_4_NPF_6_/THF at 0.2 Vs^−1^.

Encouraged by these successes, we extended the screening to the bissulfonamide **L3** (Scheme 2).^[12]^ The CVs (Figure 3) demonstrate that the wave originating from [Cp_2_Ti(III)Cl] is visible. This predicts that addition of **L3** results in an efficient bulk electrolysis and a successful radical arylation (Table 1).

**Table 1:** Results of the arylation reaction after electrochemical activation in THF with Cp_2_TiCl_2_ as precatalyst and **L1**–**L3** as additives.

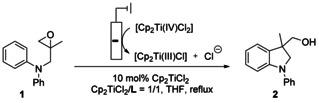
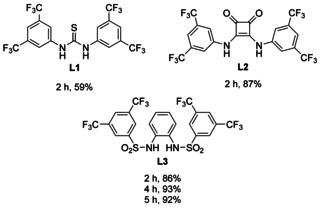

Reaction conditions: Catalyst Cp_2_TiCl_2_, additive L, 10 mm in THF, isolated yields.

The performance of the epoxide arylation^[7]^ with **1** and 10 mol % electrochemically reduced Cp_2_TiCl_2_ in the presence of 1 equiv. of **L1**–**L3** with respect to Cp_2_TiCl_2_ is summarized in Table 1. In agreement with the lowest amount of [Cp_2_Ti(III)Cl] observed in the CV‐screening, **L1** leads to the slowest reaction and lowest yield of **2. L2** and **L3** lead to a more active catalytic system giving essentially identical results after 2 h.

Besides their usefulness as predictors, the CVs highlight an interesting complexity of interactions between **L1**–**L3** and the titanocene species. On the reductive sweep (the lower trace) all ligands **L** form an adduct with [Cp_2_Ti(IV)Cl_2_]. This adduct has a more positive reduction potential with **L1** and **L2** (+160 mV) showing a larger shift than **L3** (+80 mV). These differences in the potentials indicate a different strength of the interactions. On the oxidative sweep (the upper trace), for all additives a complex [Cp_2_Ti(III)Cl_2_]^−^*L can be detected. Again, the differences in the shift of the potentials are significant. Interestingly, only **L2** and **L3** seem to form complexes of the type [Cp_2_Ti(III)Cl]*L. The shift of the potentials relative to [Cp_2_Ti(III)Cl] suggest an electron‐donating interaction for **L3** (−120 mV) and an electron‐withdrawing interaction for **L2** (+110 mV).

### Studies at the rotating ring‐disk electrode

Our CV experiments are an ideal tool for the rapid screening of the additives. For a quantitative analysis, CV merely allows the direct extraction of the redox potentials of the species detected and their diffusion coefficients. More complex information on pre‐equilibria or follow‐up reactions can only be obtained via simulation. One general shortcoming of simulation is that, frequently, several models fit the experimental data. This is especially true for situations involving several steps and a large variety of fitting parameters. We decided to provide quantitative data for our qualitative observations made by CV. To this end, we used the rotating ring‐disk electrode (RRDE).^[8]^ This allows assessing the results of future screening experiments on a more solid mechanistic basis.

The RRDE set‐up offers major advantages over CV in stagnant solution. In CV, asymmetrically shaped current waves are observed. Therefore, “real” systems cannot be fitted to a physical equation. With a RRDE set‐up this becomes possible as convection results in a time‐independent and well‐defined thickness of the Nernstian diffusion layer. It is composed of a disk and a ring electrode surrounding the disk. Rotation ensures a steady flow of reactants from the bulk solution to the disk. From here, the electrolyte is radially forced out to the ring and the species generated at the disk can be detected with a theoretical collection efficiency *N*_0_. They can be discerned by their individual redox potentials. The currents measured due to their electrochemical reactions are proportional to their concentration at the electrode surfaces and allow quantitative analysis of the composition of the solution. For potentials negative of the equilibrium of a reduction at the disk, the currents reach a plateau representing the diffusion limited current *I*_lim_ and can be evaluated by the Levich equation (see Supporting Information).^[13]^

**Scheme 3:**
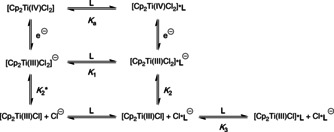
Proposed mesh‐scheme for the Cp_2_TiCl_2_/**L** redox system (**L** = **L1**, **L2** or **L3**).

We started our RRDE measurements of Cp_2_TiCl_2_ in THF with 0.2 m NBu_4_PF_6_ (TBAPF_6_). The data are in agreement with results of the Daasbjerg group[^4c^, ^4d^, ^4e^] by means of CV simulation (see Supporting Information) and show that RRDE measurements are well suited for analyzing electrochemical systems based on Cp_2_TiCl_2_. Based on Daasbjerg's work we propose an extended mesh‐scheme for the species present in solutions of Cp_2_TiCl_2_ and our additives **L** (Scheme 3). The monomer–dimer equilibrium of [Cp_2_Ti(III)Cl] is not observed in CVs with **L** and thus not relevant here.

The RRDE measurements will provide the equilibrium constants given in Scheme 3 and an estimate of the solution composition before and after reduction. We will discuss the experimental results for sulfonamide **L3** and summarize the data for **L1**–**L3** (detailed discussion see Supporting Information).

**Figure 4:**
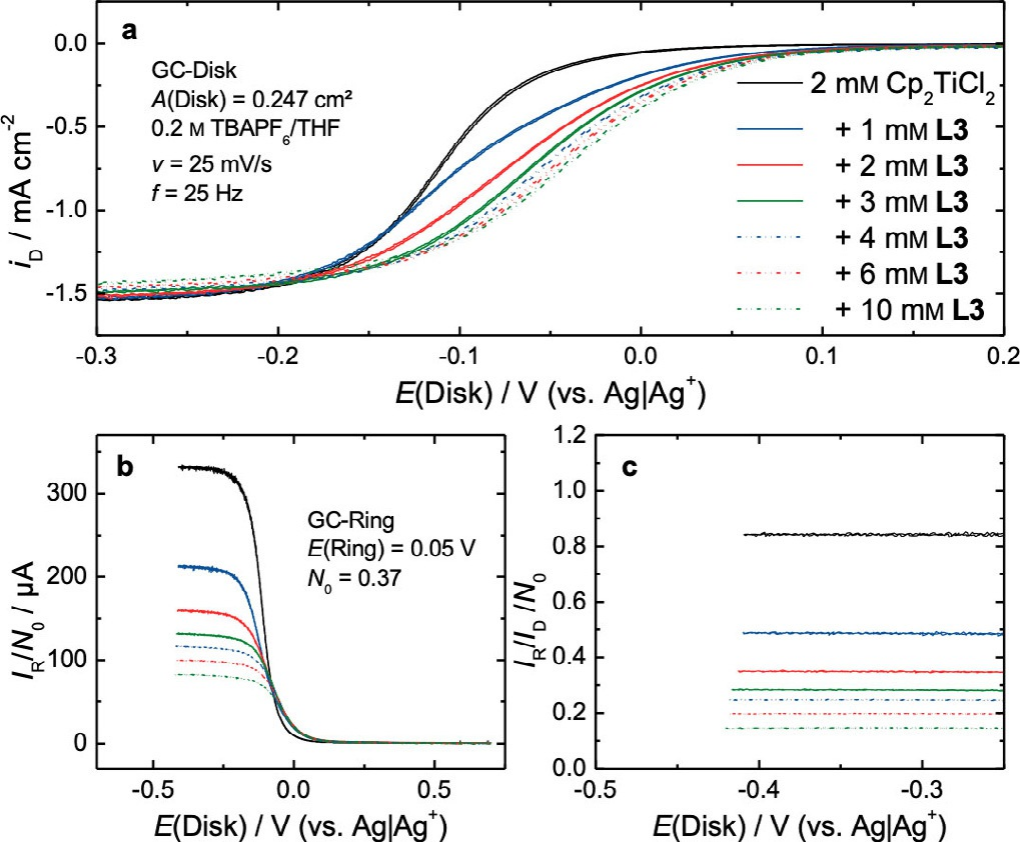
Disk current densities *i*_D_ (a), normalized ring currents *I*_R_/*N*_0_ (b) and transfer ratios *I*_R_/*I*_D_/*N*_0_ (c) of the RRDE measurements of a solution of 2 mm Cp_2_TiCl_2_ in 0.2 m NBu_4_PF_6_/THF with different concentrations of **L3** at a ring potential *E*_R_ of 0.05 V and a rotation frequency *f* of 25 Hz.

The stabilizing effect of **L3** on [Cp_2_Ti(III)Cl_2_]^−^ can be observed directly from the positive shift of the voltammograms detected at the disk *i*_D_ and the decreasing transfer ratios (*I*_R_/*I*_D_/*N*_0_) for measurements with Cp_2_TiCl_2_ and **L3** at a rotation frequency *f* of 25 Hz and a ring potential *E*_R_ of 0.05 V (Figure 4, for measurements at *f* = 4 Hz and *E*_R_ = 0.23, 0.42 and 0.66 V see Supporting Information). A potential of 0.05 V vs. Ag/Ag^+^ solely allows the oxidation of [Cp_2_Ti(III)Cl_2_]^−^ (Figure 3, please note that the Fc^+^/Fc scale is shifted by ca. −1.1 V). Thus, only this species is detected at the ring.

**L3** (10 mm, 5.0 equiv.) leads to a positive shift of the reduction potential and a slight decrease of *i*_D_ in the diffusion limited region at the disk electrode from −1.54 mA cm^−2^ to −1.46 mA cm^−2^ (Figure 4 a) and to a significant decrease of the normalized ring current *I*_R_/*N*_0_ by a factor of 4 (Figure 4 b). The transfer ratio, which is directly proportional to the concentration of [Cp_2_Ti(III)Cl_2_]^−^ in solution (Figure 4 c), decreases from 0.85 in the base electrolyte to 0.18 when adding 10 mm (5.0 equiv.) **L3**.

**L3** (1 mm, 0.5 equiv.) leads to the formation of a shoulder in the disk current at −0.05V (Figure 4 a), which allows us to obtain equilibrium constant *K*_a_ (Scheme 3). The decreased diffusion‐limited current at the disk indicates a reaction of [Cp_2_Ti(IV)Cl_2_] with **L3** to [Cp_2_Ti(IV)Cl_2_]*L3 associated with a change of the diffusion coefficient prior to the electrochemical reaction. If the reaction of **L3** occurred after the electrochemical reduction of [Cp_2_Ti(IV)Cl_2_], this should not have an impact on the diffusion‐limited current.

**Figure 5:**
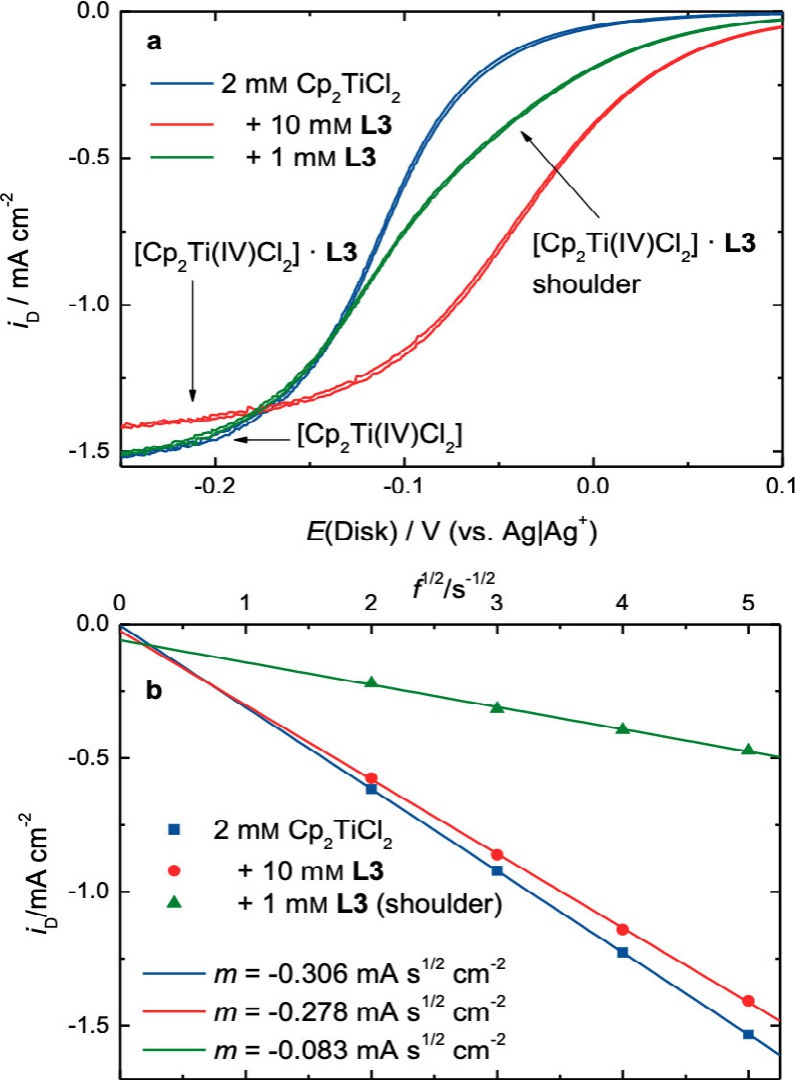
Cut‐out of the RDE measurements shown in Figure 4 a (a) highlighting the shoulder in the 1 mm **L3** measurement and diffusion limited current densities (b) at the disk *i*_D_ as a function of *f* ^1/2^ for Cp_2_TiCl_2_ and with 10 mm of **L3** or 1 mm of **L3** (shoulder). The slopes of the linear fits are given as *m*.

According to the Levich equation, the limiting current density at the disk electrode is proportional to *D* ^2/3^ (*D*: diffusion coefficient) and to *f* ^1/2^. Figure 5b depicts the diffusion‐limited *i*_D_ of [Cp_2_Ti(IV)Cl_2_] and with 10 mm of **L3** alongside the shoulder current density found for the measurement of **L3** (1 mm, 0.5 equiv.) as a function of *f* ^1/2^. The graphical representation of the data yields straight lines. The non‐zero intercept of the line representing the shoulder currents indicates that the current flow is not solely limited by diffusion. Possibly the kinetics of the reduction of the unbound species already play a minor role. We assume that in the solutions with 10 mm of **L** approximately all [Cp_2_Ti(IV)Cl_2_] is bound to **L**. The different slopes observed for the experiments with base electrolyte and with 10 mm **L3** yield the ratio of the diffusion coefficients of [Cp_2_Ti(IV)Cl_2_] and [Cp_2_Ti(IV)Cl_2_]*L3 according to the Levich equation. The diffusion coefficient *D* of [Cp_2_Ti(IV)Cl_2_] is 1.15 times larger than that of [Cp_2_Ti(IV)Cl_2_]*L3 (1.15 with **L1** and 1.33 with **L2**). In the measurement with 1 mm **L3** the diffusion‐limited reduction of [Cp_2_Ti(IV)Cl_2_]*L3 is observed as a shoulder prior to the reduction of [Cp_2_Ti(IV)Cl_2_], as the reaction between [Cp_2_Ti(IV)Cl_2_] and **L3** is incomplete. From the ratio of this shoulder to the subsequent plateau, the equilibrium constant of association *K*_a_ of **L3** to [Cp_2_Ti(IV)Cl_2_] is accessible. With a value of 1.07 mm ^−1^, **L3** lies between **L1** (0.69 mm ^−1^) and **L2** (1.81 mm ^−1^).

**Figure 6:**
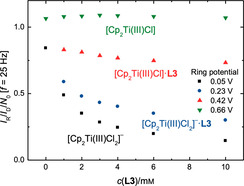
Transfer ratios *I*_R_/*I*_D_/*N*_0_ for the measurements of Cp_2_TiCl_2_ and **L3** at different concentrations of **L3** at ring potentials of 0.05, 0.23, 0.42 and 0.66 V and at a rotation frequency *f* of 25 Hz.

We studied the composition of the reduced solutions of Ti^III^ to obtain *K*_1_ to *K*_3_ (Scheme 3) by analyzing the effect of **L** on the transfer ratios (compare Figure 4 c). The fixed ring potential *E*_R_ allows the detection of all species that can be oxidized up to this potential. The step‐wise increase of *E*_R_ afforded transfer ratios for [Cp_2_Ti(III)Cl_2_]^−^, [Cp_2_Ti(III)Cl_2_]^−^*L3, [Cp_2_Ti(III)Cl] and [Cp_2_Ti(III)Cl]*L3. These transfer ratios are shown in Figure 6 as a function of the concentration of **L3**.

The transfer ratios allow the calculation of the equilibrium constants between the Ti^III^ species (see Supporting Information for details). The association equilibrium constant *K*_1_ of **L3** to [Cp_2_Ti(III)Cl_2_]^−^ amounts to 0.4 mm ^−1^ (2 mm ^−1^ for **L1**, 0.3 mm ^−1^ for **L2**). Dissociation of Cl^−^*L3 from [Cp_2_Ti(III)Cl_2_]^−^*L3 has an equilibrium constant *K*_2_ of 3.0 mm (2.8 mm for **L1**, 54 mm for **L2**). The formation of [Cp_2_Ti(III)Cl]*L3 has a *K*_3_ of 0.7 mm ^−1^ (0.5 mm ^−1^ for **L2**). This adduct is not observed for **L1**.

**Figure 7:**
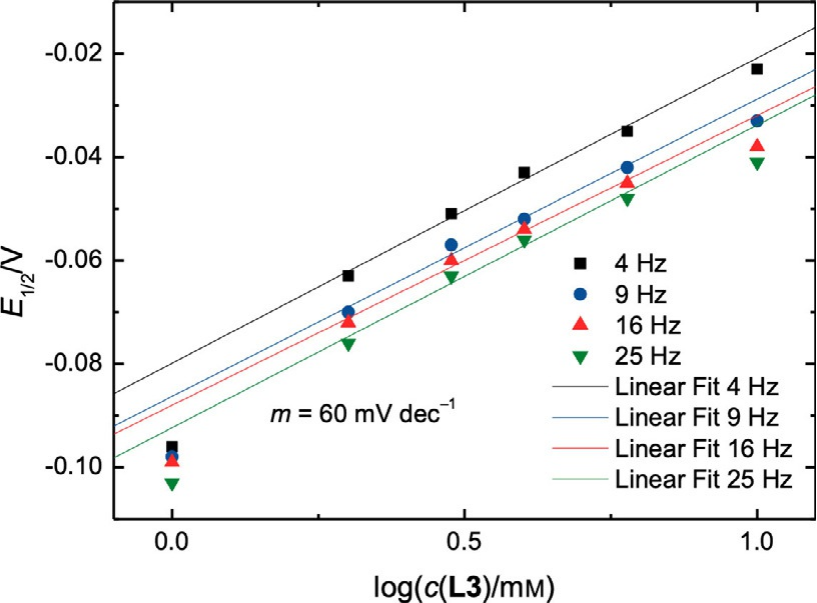
Plots of the half‐wave potential *E*_1/2_ of the RRDE measurements with Cp_2_TiCl_2_ and **L3** as a function of the decadic logarithm of the concentration of **L3**.

For these equilibria we have assumed the formation of 1:1 adducts. The concentration‐dependent measurements can verify this assumption. The voltammograms of *i*_D_ (Figure 4 a) show a gradual shift of the reduction potential starting from a concentration of 2 mm of **L3**. This implies that the Nernst equation for this redox process depends on the concentration of free **L3**. When plotting the half‐wave potential *E*_1/2_ against the decadic logarithm of the concentration of **L3** (Figure 7) a linear relation with a slope *m* of roughly 60 mV dec^−1^ at all rotation frequencies is obtained.

Therefore, the Nernst equation for this redox system must be linearly dependent on log(*c*(**L3**)^*p*^) with *p* representing the number of **L3** ligands attached to the titanocene.^[14]^ With **L1** the same slope of 60 mV dec^−1^ was obtained showing that *p* is equal to 1. This verifies our initial assumption. The mechanisms of chloride abstraction from the Ti^III^ complexes and activation of the Ti^IV^ complexes are likely to be similar for **L1** and **L3**.

**Figure 8:**
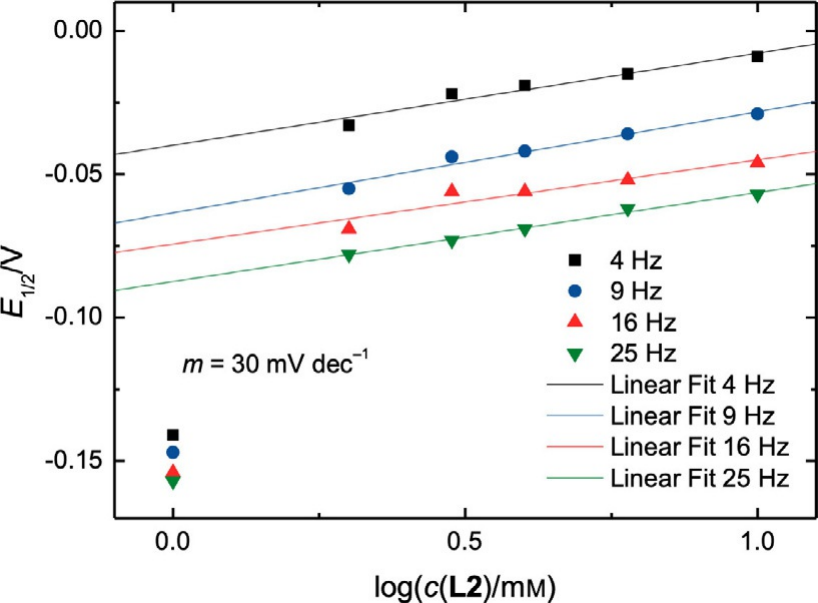
Plots of the half‐wave potential *E*_1/2_ of the RRDE measurements with Cp_2_TiCl_2_ and **L2** as a function of the decadic logarithm of the concentration of **L2**.

For **L2** a slope of 30 mV dec^−1^ was observed (Figure 8). Thus, the redox potential is proportional to log(*c*(**L2**)^*q*^) and *q* = *p*/2. Thus, we propose the reversible formation of a 2:1 adduct between [Cp_2_Ti(III)Cl] and **L2** in addition to the equilibria shown in Scheme 3. For the case that only this 2:1 adduct formation between [Cp_2_Ti(III)Cl] and **L2** is considered (in contrast to the 1:1 adduct considered for *K*_1_ to *K*_3_ given above) a new set of equilibrium constants for the Ti^III^ species has to be calculated (*K*_1_’ = 0.2 mm ^−1^, *K*_2_’ = *K*_2_ = 54 mm, *K*_3_’ = 0.1 mm ^−2^). However, it is likely that both adducts are present in the reduced solutions.

**Scheme 4:**
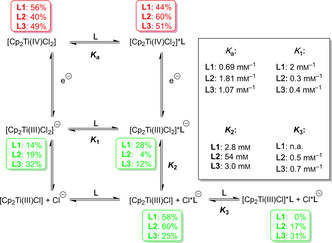
Mesh‐scheme for the Cp_2_TiCl_2_/**L** redox system (**L** = **L1**, **L2** or **L3**) including the equilibrium constants obtained in the RRDE measurements and the solution composition in % [Ti^IV^ in red, Ti^III^ in green].

The equilibrium constants obtained from the RRDE‐studies together with the relative amounts of the relevant Ti^IV^ (in red) and Ti^III^ species (in green) at concentrations of 2 mm in Ti and **L** are summarized in Scheme 4. [Cp_2_Ti(IV)Cl_2_] is complexed in 40 %–60 % amounts (red numbers) by **L1**–**L3** with **L2** being slightly more efficient than the other additives as indicated by the *K*_a_ values.

The additives **L1**–**L3** have a stronger influence on the equilibria involving [Cp_2_Ti(III)Cl]. In the presence of **L2**, essentially no [Cp_2_Ti(III)Cl_2_]^−^*L2 is formed because **L2** binds Cl^−^ too strongly. Interestingly, **L2** complexes [Cp_2_Ti(III)Cl] less efficiently than **L3** whereas **L1** forms no [Cp_2_Ti(III)Cl]*L1. As a result, the use of **L2** results in the highest combined relative amount (77 %) of the catalytically active [Cp_2_Ti(III)Cl] and [Cp_2_Ti(III)Cl]*L2. For **L1** and **L3**, the solution contains relatively high proportions of [Cp_2_Ti(III)Cl_2_]^−^*L and [Cp_2_Ti(III)Cl_2_]^−^ that constitute the resting state of the active catalyst. This situation may be advantageous when catalyst stability is more important than catalyst activity.

The RRDE investigations underline that our use of the E_q_C_r_ equilibrium as predictor for the performance of the bulk electrolysis of Cp_2_TiCl_2_ in THF as well as for the titanocene catalyzed radical arylation of epoxides has a sound mechanistic basis. Moreover, the quantitative data obtained allow an accurate prediction of the composition of an electrochemically reduced solution of Cp_2_TiCl_2_ containing additives that is essential for a precise control of the reaction conditions. This may be essential for applications on large scale.

### Density functional theory studies

To relate the data obtained from RRDE and CV measurements to molecular properties, we studied the structures and energies of the species involved in the E_q_C_r_ equilibrium by DFT methods. The Grimme group has recently developed a powerful multilevel approach to address such situations. It consists of the CREST code^[15]^ for searching the low‐energy chemical space by tight‐binding semi‐empirical theory based *meta*‐dynamics (MTD) calculations (see Ref. [16]). The resulting conformer ensemble is refined efficiently in multiple DFT steps with the ENSO code^[17]^ as a driver for ORCA or TURBOMOLE quantum chemistry packages.^[18]^ For a recent overview of the main xTB code used for the GFN tight‐binding or force field calculations see Ref. [19].

Accordingly, we used the xTB, CREST and ENSO programs to determine the structures with the lowest free energy in THF solution for each ligand **L** and all complexes. These calculations were conducted in the same workflow. Manually prepared starting structures, which are initially preoptimized with the GFN2‐xTB[GBSA] tight binding model are used in the CREST program that employs MTD at the same level in order to obtain a relative complete ensemble of likely structures. The ENSO program determines the equilibrium (Boltzmann) populations for a few low‐lying conformers at higher theoretical levels in three steps. First, already relatively accurate B97‐3c[DCOSMO‐RS(THF)] (a composite low‐cost DFT method^[20]^) single point energies are calculated on the CREST ensemble. Structures within an energy threshold of 4 kcal mol^−1^ above the lowest lying structure are then fully optimized at the same level. In this first filtering step thermostatistical free energies in the modified rigid‐rotor/harmonic‐oscillator (mRRHO) approximation^[21]^ calculated with GFN2‐xTB[GBSA] and the free energy of solvation in THF calculated with the accurate COSMO‐RS^[22]^ solvation model are added. Finally, for all structures within a 2 kcal mol^−1^ threshold an even better single point energy is computed at the PW6B95‐D3/def2‐TZVPP^[23]^ hybrid DFT level which basically replaces the corresponding B97‐3c energy. In summary, the final complete total free energy used consists of the mRRHO part from the GFN2‐xTB treatment, the COSMO‐RS part in THF for solvation and the basic electronic energy with the PW6B95‐D3 functional. In the following, the conformer of each species with the lowest total free energy is given, if not stated otherwise.

### Structures of L and Cl^−^*L

We started our investigations with the ligands **L** and their chloride complexes Cl^−^*L. As free species, **L2** shows higher conformational rigidity than **L1** and **L3** (for a detailed discussion see Supporting Information). The structures of the most stable conformers were chosen as reference points for the energies of formation of all other complexes. A potential self‐aggregation of **L2** in THF is beyond the scope of this study.^[24]^

**Figure 9:**
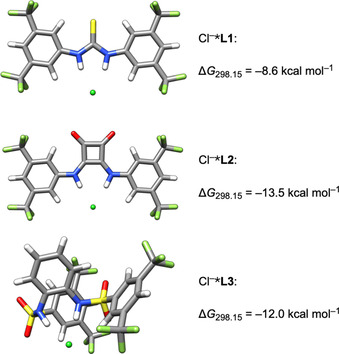
DFT structures of the Cl^−^*L adducts of **L1**, **L2** and **L3**.

The structures of the Cl^−^*L and energies of complexation with respect to the lowest energy conformer of the respective **L** are depicted in Figure 9. Cl^−^ is bound to both N−H groups of all ligands. The Cl^−^*L complexes with **L1** and **L2** show *C*_2*v*_ symmetry. Cl^−^*L2 is conformationally rigid in agreement with its X‐ray structure.^[25]^ Cl^−^*L1 has a second conformer 4.6 kcal mol^−1^ higher in energy than the one shown with one NHR group rotated by almost 180° and the other bound to Cl^−^. A second conformer of Cl^−^*L3 is 2.0 kcal mol^−1^ higher in energy and has a similar structure to the one shown. In agreement with a previous study on the chloride binding of thioureas, squaramides and sulfonamides,^[12]^ Δ*G*_298.15_ for Cl^−^*L show that **L2** binds chloride stronger than **L1** and **L3**. As also pointed out in that study, the strength of chloride binding does not correlate with the p*K*_a_ values of the different receptors.

### Supramolecular complexes of L and Ti^III^

The supramolecular complexes [Cp_2_Ti(III)Cl_2_]^−^*L are crucial intermediates in the electrochemical generation of [Cp_2_Ti(III)Cl] from [Cp_2_Ti(IV)Cl_2_]. They provide a mechanism for shifting the E_q_C_r_ equilibrium to [Cp_2_Ti(III)Cl] by facilitating chloride abstraction and dissolution of [Cp_2_Ti(III)Cl_2_]^−^*L. Another intriguing aspect is that binding of **L** to [Cp_2_Ti(III)Cl] directly impacts the redox properties of [Ti] in the arylation reaction.

**Figure 10:**
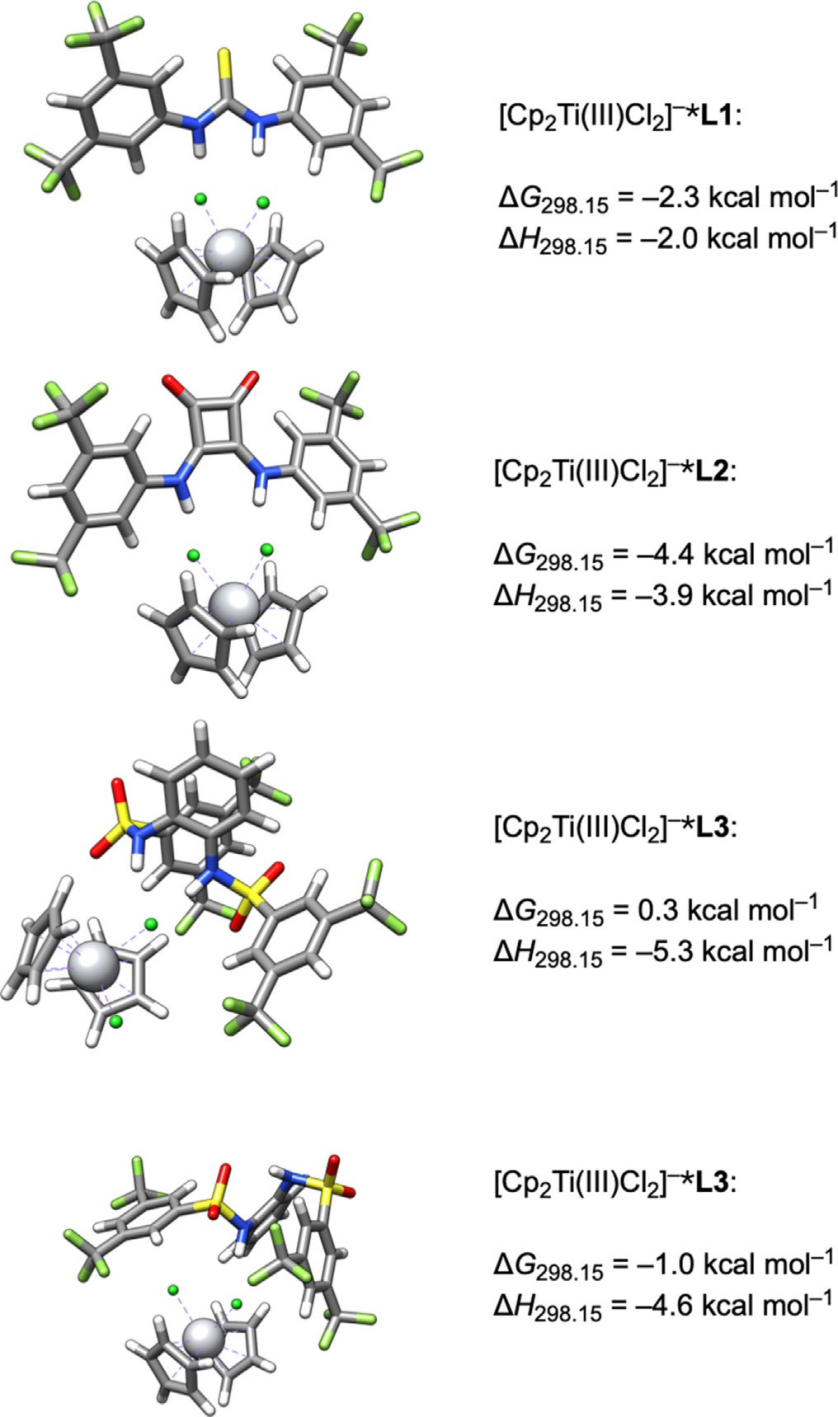
DFT structures of the [Cp_2_Ti(III)Cl_2_]^−^*L adducts of **L1**, **L2** and **L3**.

The structures of [Cp_2_Ti(III)Cl_2_]^−^*L together with the corresponding values for Δ*G*_298.15_ and Δ*H*_298.15_ are depicted in Figure 10 and reveal that **L1** and **L2** bind both chloride ligands with the N−H groups, providing complexes that are *C*_2_‐symmetric within the limits of accuracy. This is exemplified by the Ti‐Cl bond lengths ([Cp_2_Ti(III)Cl_2_]^−^*L1: 2.545 Å and 2.540 Å, [Cp_2_Ti(III)Cl_2_]^−^*L2: 2.547 Å and 2.550 Å).

Curiously, for **L3** a binding of both N−H groups was not observed. In the more stable complex (Figure 10, lowest structure) **L3** only binds to one of the chloride ligands with one N−H group (Ti−Cl bond lengths: 2.53 Å and 2.50 Å). In the second most stable complex (3rd structure from the top of Figure 10), both N−H groups coordinate to one chloride in a “side‐on” geometry. This results in distinctly different Ti−Cl bond lengths (2.59 Å and 2.49 Å). Enthalpically, the less stable complex is slightly favored (Δ*H*_298.15_ = −5.3 vs. −4.6 kcal mol^−1^). However, the entropically disfavored restriction of conformational freedom in this binding mode is the more relevant contribution to Δ*G*_298.15_. According to the values for Δ*G*_298.15_, the binding via both N−H and Ti−Cl groups by **L1** and **L2** results in a stronger binding of [Cp_2_Ti(III)Cl_2_]^−^.

**Figure 11:**
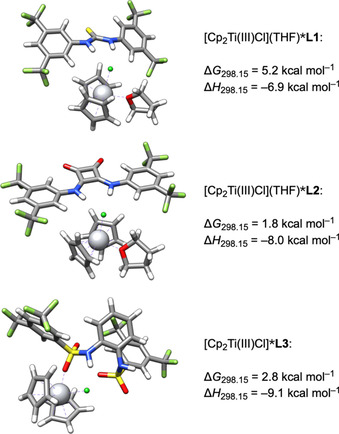
DFT structures of the [Cp_2_Ti(III)Cl](THF)*L adducts of **L1** and **L2** and of [Cp_2_Ti(III)Cl]*L3.

According to the CV and RRDE measurements, **L1** does not bind to [Cp_2_Ti(III)Cl] whereas both **L2** and **L3** do. However, they bind in a different fashion as evident from the shifted oxidation potentials for [Cp_2_Ti(III)Cl]*L (**L2**: +110 mV, **L3**: −120 mV). We analyzed the complex formation starting from [Cp_2_Ti(III)Cl] and **L** and included the effect of additional coordination with THF (Figure 11, also see Supporting Information).

**L1** and **L2** display a similar complexation behavior. Adduct formation is substantially more favorable with [Cp_2_Ti(III)Cl](THF). As for [Cp_2_Ti(III)Cl_2_]^−^ both additives bind the chloride ligand with both N−H groups. Adduct formation is disfavored with **L1**. This is in agreement with the experiment, where no [Cp_2_Ti(III)Cl]*L1 was observed. The interaction of **L2** with the chloride ligand leads to an increase of the Ti−Cl bond length by 0.13 Å. This is in agreement with the experimentally observed positive shift of the redox potential.

The situation is different for **L3**. The favored complexation mode is realized with [Cp_2_Ti(III)Cl] as starting material. This is due to an intramolecular interaction of one of the sulfonamide groups with Ti that renders an additional complexation of THF superfluous. The coordination of the sulfonamide increases the electron density at Ti in agreement with the experimentally observed negative shift in the redox potential for [Cp_2_Ti(III)Cl]*L3. Only the adjacent N−H group binds to the chloride ligand.

**Figure 12:**
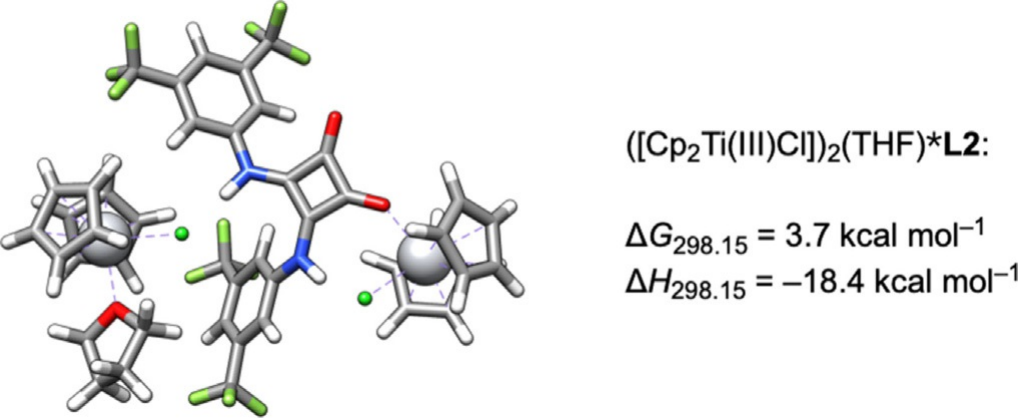
DFT structure of the ([Cp_2_Ti(III)Cl])_2_(THF)*L2 adduct.

The slope of 30 mV dec^−1^ in the RRDE measurements with **L2** (Figure 8) suggests that a 2:1 stoichiometry of [Cp_2_Ti(III)Cl] and **L2** in the adduct formation is possible. The computed structure of the 2:1 complex with one molecule of THF and its Δ*G*_298.15_ and Δ*H*_298.15_ with respect to the dissociated species are depicted in Figure 12. Complex formation without THF is distinctly less favorable.

Enthalpically, complex formation is strongly favored and the slightly positive Δ*G*_298.15_ is due to the entropically disadvantageous formation of the complex from four components.

**L2** binds the second titanocene through one of the carbonyl oxygens. At the same time, the N−H groups each bind to one of the chloride ligands of both titanocenes. This makes **L2** a bifunctional ligand and provides an explanation for the broad oxidation wave in the area of Cp_2_TiCl found in CVs of Cp_2_TiCl_2_ and **L2**. On the oxidative sweep ([Cp_2_Ti(III)Cl])_2_*L2 is oxidized first followed by [Cp_2_Ti(III)Cl] and finally [Cp_2_Ti(III)Cl]*L2.

### Supramolecular complexes of L and Ti^IV^

**Figure 13:**
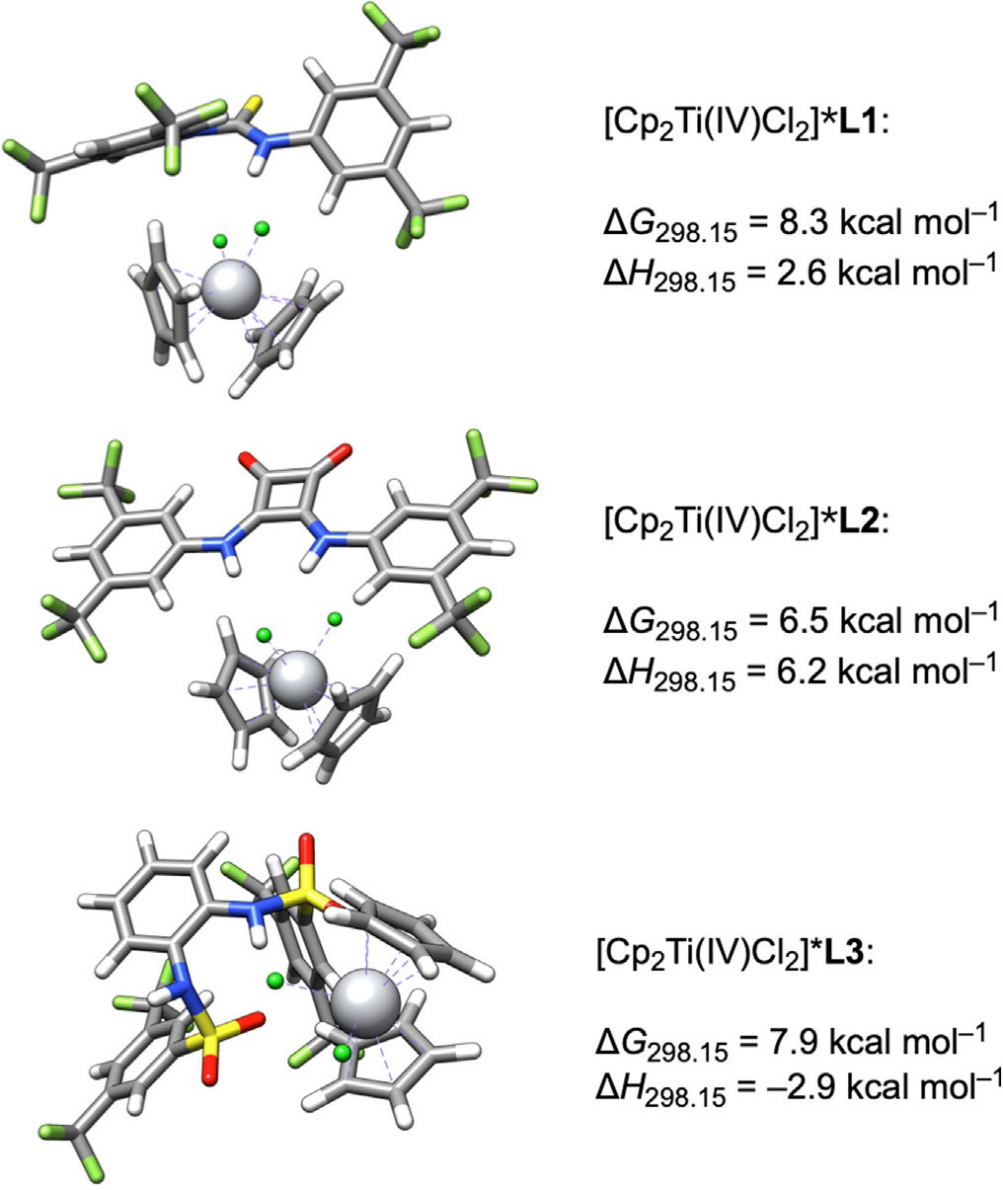
DFT structures of the [Cp_2_Ti(IV)Cl_2_]*L adducts of **L1**, **L2** and **L3**.

Finally, we investigated the adduct formation between [Cp_2_Ti(IV)Cl_2_] and **L**. This pre‐equilibrium to the E_q_C_r_ mechanism allows the use of a less negative reduction potential in bulk electrolysis (−1.3 V vs. Ag/Ag^+^ with **L2**, −1.4 V with **L1** and **L3**). Figure 13 shows the structures of the hydrogen bonded adducts between [Cp_2_Ti(IV)Cl_2_] and **L1**–**L3**. For **L2**, the minimum structure of [Cp_2_Ti(IV)Cl_2_]*L2 is a van der Waals complex that is 0.3 kcal mol^−1^ more stable than the complex shown (see Supporting Information for details). It is not discussed here, as it does not suggest a positive shift of the reduction potential.

Compared to the complexes of [Cp_2_Ti(III)Cl_2_]^−^*L the formation of the adducts of [Cp_2_Ti(IV)Cl_2_]*L is less favorable. The calculated structures show that with the neutral Ti^IV^ complex only **L2** can interact with both N−H groups. However, they both bind to the same chloride ligand. In the adducts of **L1** and **L3** only one of the N−H groups is coordinating chloride, the other is pointing away from the complex resulting in a noticeable conformational change of the additive.

The computed order of stability of the adducts is in agreement with the stronger binding affinity of **L2** to [Cp_2_Ti(IV)Cl_2_] observed in the RRDE measurements.

**Scheme 5:**
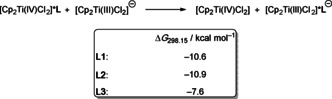
Gibbs energies of the exchange reaction between [Cp_2_Ti(IV)Cl_2_]*L and [Cp_2_Ti(III)Cl_2_]^−^.

The lower stability of [Cp_2_Ti(IV)Cl_2_]*L compared to [Cp_2_Ti(III)Cl_2_]^−^*L is most easily rationalized by the negative charge in [Cp_2_Ti(III)Cl_2_]^−^ that renders hydrogen bonding more attractive because of the coulomb attraction. We investigated this point by analyzing the exchange reactions shown in Scheme 5. The effect of the charge is indeed substantial as highlighted by the highly exergonic formation of the [Cp_2_Ti(III)Cl_2_]^−^*L adducts from their [Cp_2_Ti(IV)Cl_2_]*L counterparts.

## Conclusions

In summary, we have demonstrated that our approach of screening the E_q_C_r_ equilibrium as predictor for the bulk electrolysis of Cp_2_TiCl_2_ in THF and the titanocene catalyzed radical arylation of epoxides has a sound mechanistic basis. This was achieved by a combination of synthesis, the application of the electrochemical techniques cyclic voltammetry (CV) and rotating ring‐disk electrode (RRDE) measurements, as well as DFT investigations.

We have introduced bissulfonamides as additives for enabling bulk electrolysis of Cp_2_TiCl_2_ in THF as well as for the titanocene catalyzed radical arylation of epoxides. For the first time, we have provided quantitative data about the E_q_C_r_ equilibrium in the presence of a thiourea, a squaramide and a bissulfonamide and have established the stoichiometry of adduct formations between [Cp_2_Ti(III)Cl_2_]^−^, [Cp_2_Ti(III)Cl] and [Cp_2_Ti(IV)Cl_2_]. By studying the complexes by DFT methods, we have provided the Gibbs energies and enthalpies of complexation as well as the adduct structures. Our studies show that a balance between activity and stability of the catalyst are vital for reaction efficiency. Changing the inorganic ligand of Cp_2_TiX_2_ was not investigated here. Previous investigations^[5]^ have shown that for Cp_2_TiBr_2_ derived catalysts halide abstraction is easier than for complexes derived from Cp_2_TiCl_2_. The intricate interactions between the additives and the titanocenes by hydrogen bonding and coordination of polar groups are not only in agreement with the electrochemical data. They also provide a design platform for the development of novel additives for catalysis in single electron steps^[26]^ or metalloradical catalysis^[27]^ in particular in enantioselective catalysis.

## Conflict of interest

The authors declare no conflict of interest.

## Supporting information

As a service to our authors and readers, this journal provides supporting information supplied by the authors. Such materials are peer reviewed and may be re‐organized for online delivery, but are not copy‐edited or typeset. Technical support issues arising from supporting information (other than missing files) should be addressed to the authors.

Supplementary

## References

[1a] P. T. Anastas , M. M. Kirchhoff , Acc. Chem. Res. 2002, 35, 686–694; 12234198 10.1021/ar010065m

[1b] Efficient screening processes are essential for green chemistry approaches, see: C. W. Coley , N. S. Eyke , K. F. Jensen , Angew. Chem. Int. Ed. 2020, 10.1002/anie.201909987, Angew. Chem. **2020**, 10.1002/anie.201909987,;

[1c] C. W. Coley , N. S. Eyke , K. F. Jensen , Angew. Chem. Int. Ed. 2020, 10.1002/anie.201909989 Angew. Chem. **2020**, 10.1002/anie.201909989;

[1d] S. Ahn , M. Hong , M. Sundararajan , D. H. Ess , M.-H. Baik , Chem. Rev. 2019, 119, 6509–6560; 31066549 10.1021/acs.chemrev.9b00073

[1e] for selected examples see: M. N. Hopkinson , A. Gómez-Suárez , M. Teders , B. Sahoo , F. Glorius , Angew. Chem. Int. Ed. 2016, 55, 4361–4366; 10.1002/anie.201600995 27000485

[1f] M. Teders , A. Gómez-Suárez , L. Pitzer , M. N. Hopkinson , F. Glorius , Angew. Chem. Int. Ed. 2017, 56, 902–906; 10.1002/anie.201609393 28000346

[2a] A. Gansäuer , A. Fleckhaus , M. Alejandre Lafont , A. Okkel , K. Kotsis , A. Anoop , F. Neese , J. Am. Chem. Soc. 2009, 131, 16989–16999; 19919150 10.1021/ja907817y

[2b] A. Gansäuer , S. Hildebrandt , E. Vogelsang , R. A. Flowers II , Dalton Trans. 2016, 45, 448–452. 26575367 10.1039/c5dt03891j

[3] J. I. van der Vlugt , Chem. Eur. J. 2019, 25, 2651–2662. 30084211 10.1002/chem.201802606 PMC6471147

[4a] Y. Mugnier , C. Moise , E. Laviron , J. Organomet. Chem. 1981, 204, 61–66;

[4b] E. Samuel , J. Vedel , Organometallics 1989, 8, 237–241;

[4c] R. J. Enemærke , J. Larsen , T. Skrydstrup , K. Daasbjerg , Organometallics 2004, 23, 1866–1874;

[4d] R. J. Enemærke , J. Larsen , T. Skrydstrup , K. Daasbjerg , J. Am. Chem. Soc. 2004, 126, 7853–7864; 15212533 10.1021/ja0491230

[4e] R. J. Enemærke , J. Larsen , G. H. Hjøllund , T. Skrydstrup , K. Daasbjerg , Organometallics 2005, 24, 1252–1262.

[5] CV-Screening:

[5a] T. Liedtke , P. Spannring , L. Riccardi , A. Gansäuer , Angew. Chem. Int. Ed. 2018, 57, 5006–5010; 10.1002/anie.201800731 29488673

[5b] T. Liedtke , T. Hilche , S. Klare , A. Gansäuer , ChemSusChem 2019, 12, 3166–3171. 30779429 10.1002/cssc.201900344

[6] Arylation:

[6a] P. Wipf , J. P. Maciejewski , Org. Lett. 2008, 10, 4383–4386; 18781767 10.1021/ol801860s PMC5467310

[6b] A. Gansäuer , M. Behlendorf , D. von Laufenberg , A. Fleckhaus , C. Kube , D. V. Sadasivam , R. A. Flowers II , Angew. Chem. Int. Ed. 2012, 51, 4739–4742; 10.1002/anie.201200431 22461392

[6c] A. Gansäuer , C. Kube , K. Daasbjerg , R. Sure , S. Grimme , G. D. Fianu , D. V. Sadasivam , R. A. Flowers II , J. Am. Chem. Soc. 2014, 136, 1663–1671; 24397383 10.1021/ja4121567

[6d] A. Gansäuer , D. von Laufenberg , C. Kube , T. Dahmen , A. Michelmann , M. Behlendorf , R. Sure , M. Seddiqzai , S. Grimme , D. V. Sadasivam , G. D. Fianu , R. A. Flowers II , Chem. Eur. J. 2015, 21, 280–289; 25351963 10.1002/chem.201404404

[6e] A. Gansäuer , S. Hildebrandt , A. Michelmann , T. Dahmen , D. von Laufenberg , C. Kube , G. D. Fianu , R. A. Flowers II , Angew. Chem. Int. Ed. 2015, 54, 7003–7006; 10.1002/anie.201501955 25924582

[6f] R. B. Richrath , T. Olyschläger , S. Hildebrandt , D. G. Enny , G. D. Fianu , R. A. Flowers II , A. Gansäuer , Chem. Eur. J. 2018, 24, 6371–6379; 29327511 10.1002/chem.201705707

[6g] F. Mühlhaus , H. Weißbarth , T. Dahmen , G. Schnakenburg , A. Gansäuer , Angew. Chem. Int. Ed. 2019, 58, 14208–14212; 10.1002/anie.201908860 PMC6852184 31394024

[6h] Comparison to 5-*exo* cyclization: A. Gansäuer , M. Seddiqzai , T. Dahmen , R. Sure , S. Grimme , Beilstein J. Org. Chem. 2013, 9, 1620–1629; 24062821 10.3762/bjoc.9.185 PMC3778327

[6i] A. Gansäuer , M. Pierobon , Synlett 2000, 1357–1359.

[7] With H_2_O as supramolecular chloride binder, Cp_2_Ti^+^ is irreversibly formed:

[7a] A. Gansäuer , M. Behlendorf , A. Cangönül , C. Kube , J. M. Cuerva , J. Friedrich , M. van Gastel , Angew. Chem. Int. Ed. 2012, 51, 3266–3270; *Angew. Chem*. **2012**, *124*, 3320–3324. 10.1002/anie.201107556 22337565

[7b] For the use of this concepts with other metals see: E. P. Farney , S. J. Chapman , W. B. Swords , M. D. Torelli , R. J. Hames , T. P. Yoon , J. Am. Chem. Soc. 2019, 141, 6385–6391. 30897327 10.1021/jacs.9b01885 PMC6519111

[8a] C. Sandford , M. A. Edwards , K. J. Klunder , D. P. Hickey , M. Li , K. Barman , M. S. Sigman , H. S. White , S. D. Minteer , Chem. Sci. 2019, 10, 6404–6422; 31367303 10.1039/c9sc01545k PMC6615219

[8b] W. J. Albery , Trans. Faraday Soc. 1966, 62, 1915–1919;

[8c] W. J. Albery , S. Bruckenstein , Trans. Faraday Soc. 1966, 62, 1920–1931;

[8d] K. B. Prater , A. J. Bard , J. Electrochem. Soc. 1970, 117, 1517–1520;

[8e] A. J. Bard , L. R. Faulkner , Electrochemical Methods , 2nd ed., Wiley, New York, 2001, Chapter 9;

[8f] W. J. Albery , M. L. Hitchman , Ring-Disc Electrodes, Clarendon, Oxford, 1971.

[9a] A. J. Bard , L. R. Faulkner , Electrochemical Methods: Fundamentals and Applications , 2nd ed., Wiley, New York, 2001;

[9b] A. Jutand , Chem. Rev. 2008, 108, 2300–2347; 18605756 10.1021/cr068072h

[9c] W. E. Geiger , Coord. Chem. Rev. 2013, 257, 1459–1471.

[10a] A. Wittkopp , P. R. Schreiner , Chem. Eur. J. 2003, 9, 407–414; 12532289 10.1002/chem.200390042

[10b] K. M. Lippert , K. Hof , D. Gerbig , D. Ley , H. Hausmann , S. Guenther , P. R. Schreiner , Eur. J. Org. Chem. 2012, 5919–5927;

[10c] G. Jakab , C. Tancon , Z. Zhang , K. M. Lippert , P. R. Schreiner , Org. Lett. 2012, 14, 1724–1727; 22435999 10.1021/ol300307c

[10d] Y. Takemoto , Org. Biomol. Chem. 2005, 3, 4299–4306; 16327888 10.1039/b511216h

[10e] Z. Zhang , P. R. Schreiner , Chem. Soc. Rev. 2009, 38, 1187–1198. 19421588 10.1039/b801793j

[11] Synthesis:

[11a] A. Rostami , A. Colin , X. Y. Li , M. G. Chudzinski , A. J. Lough , M. S. Taylor , J. Org. Chem. 2010, 75, 3983–3992; 20486682 10.1021/jo100104g

[11b] Recent Application: L. Hu , M. Rombola , V. H. Rawal , Org. Lett. 2018, 20, 5384–5388.3013329310.1021/acs.orglett.8b02301

[12] V. Amendola , L. Fabbrizzi , L. Mosca , F.-P. Schmidtchen , Chem. Eur. J. 2011, 17, 5972–5981. 21472802 10.1002/chem.201003411

[13] As cited in: A. J. Bard , L. R. Faulkner , Electrochemical Methods: Fundamentals and Applications , 2nd ed., Wiley, New York, 2001, p. 339.

[14] J.-M. Saveant , J. Phys. Chem. B 2001, 105, 8995–9001.

[15] P. Pracht , F. Bohle , S. Grimme , Phys. Chem. Chem. Phys. 2020, 22, 7169–7192. 32073075 10.1039/c9cp06869d

[16] S. Grimme , J. Chem. Theory Comput. 2019, 15, 2847–2862. 30943025 10.1021/acs.jctc.9b00143

[17] S. Grimme , C. Bannwarth , S. Dohm , A. Hansen , J. Pisarek , P. Pracht , J. Seibert , F. Neese , Angew. Chem. Int. Ed. 2017, 56, 14763–14769; 10.1002/anie.201708266 PMC5698732 28906074

[18] For ORCA see:

[18a] F. Neese , Wiley Interdiscip. Rev.: Comput. Mol. Sci. 2012, 2, 73–78;

[18b] F. Neese , Wiley Interdiscip. Rev.: Comput. Mol. Sci. 2018, 8, e1327;

[18c] for TURBOMOLE see: R. Ahlrichs , M. Baer , M. Haeser , H. Horn , C. Koelmel , Chem. Phys. Lett. 1989, 162, 165–169;

[18d] O. Treutler , R. Ahlrichs , J. Chem. Phys. 1995, 102, 346–354.

[19] C. Bannwarth , E. Caldeweyher , S. Ehlert , A. Hansen , P. Pracht , J. Seibert , S. Spicher , S. Grimme , Wiley Interdiscip. Rev. Comput. Mol. Sci. 2020, 10.1002/wcms.1493.

[20] J. G. Brandenburg , C. Bannwarth , A. Hansen , S. Grimme , J. Chem. Phys. 2018, 148, 064104. 29448802 10.1063/1.5012601

[21] S. Grimme , Chem. Eur. J. 2012, 18, 9955–9964. 22782805 10.1002/chem.201200497

[22] C. C. Pye , T. Ziegler , E. van Lenthe , J. N. Louwen , Can. J. Chem. 2009, 87, 790.

[23a] Y. Zhao , D. G. Truhlar , J. Phys. Chem. A 2005, 109, 5656–5667; 16833898 10.1021/jp050536c

[23b] S. Grimme , J. Antony , S. Ehrlich , H. Krieg , J. Chem. Phys. 2010, 132, 154104; 20423165 10.1063/1.3382344

[23c] S. Grimme , S. Ehrlich , L. Goerigk , J. Comput. Chem. 2011, 32, 1456–1465; 21370243 10.1002/jcc.21759

[23d] F. Weigend , R. Ahlrichs , Phys. Chem. Chem. Phys. 2005, 7, 3297–3305. 16240044 10.1039/b508541a

[24] M. Rombola , C. S. Sumaria , T. D. Montgomery , V. H. Rawal , J. Am. Chem. Soc. 2017, 139, 5297–5300. 28375610 10.1021/jacs.7b01115

[25] N. Busschaert , R. B. P. Elmes , D. D. Czech , X. Wu , I. L. Kirby , E. M. Peck , K. D. Henzdel , S. K. Shaw , B. Chan , B. D. Smith , K. A. Joffe , P. A. Gale , Chem. Sci. 2014, 5, 3617–3626. 26146535 10.1039/C4SC01629G PMC4486358

[26a] G. Frey , J. N. Hausmann , J. Streuff , Chem. Eur. J. 2015, 21, 5693–5696; 25712472 10.1002/chem.201500102

[26b] D. S. G. Henriques , K. Zimmer , S. Klare , A. Meyer , E. Rojo-Wiechel , M. Bauer , R. Sure , S. Grimme , O. Schiemann , R. A. Flowers II , A. Gansäuer , Angew. Chem. Int. Ed. 2016, 55, 7671–7675; 10.1002/anie.201601242 27125466

[26c] N. Funken , F. Mühlhaus , A. Gansäuer , Angew. Chem. Int. Ed. 2016, 55, 12030–12034; 10.1002/anie.201606064 27600090

[26d] W. Hao , X. Y. Wu , J. Z. Sun , J. N. C. Siu , S. N. MacMillan , S. Lin , J. Am. Chem. Soc. 2017, 139, 12141–12144; 28825816 10.1021/jacs.7b06723

[26e] Y.-Q. Zhang , E. Vogelsang , Z.-W. Qu , S. Grimme , A. Gansäuer , Angew. Chem. Int. Ed. 2017, 56, 12654–12657; 10.1002/anie.201707673 28833905

[26f] W. Hao , J. H. Harenberg , S. N. MacMillan , S. Lin , J. Am. Chem. Soc. 2018, 140, 3514–3517; 29465998 10.1021/jacs.7b13710

[26g] C. Yao , T. Dahmen , A. Gansäuer , J. Norton , Science 2019, 364, 764–767; 31123133 10.1126/science.aaw3913

[26h] L. H. Leijendekker , J. Weweler , T. M. Leuther , D. Kratzert , J. Streuff , Chem. Eur. J. 2019, 25, 3382–3390; 30615817 10.1002/chem.201805909

[26i] Z. Zhang , T. Hilche , D. Slak , N. Rietdijk , U. N. Oloyede , R. A. Flowers II , A. Gansäuer , Angew. Chem. Int. Ed. 2020, 59, 9355–9359; 10.1002/anie.202001508 PMC7317808 32216162

[27a] M. Lankelma , A. M. Olivares , B. de Bruin , Chem. Eur. J. 2019, 25, 5658–5663; 30844097 10.1002/chem.201900587 PMC6563703

[27b] M. Zhou , M. Lankelma , J. I. van der Vlugt , B. de Bruin , Angew. Chem. Int. Ed. 2020, 59, 11073–11079; 10.1002/anie.202002674 PMC7317878 32259369

[27c] K. Lang , S. Torker , L. Wojtas , X. P. Zhang , J. Am. Chem. Soc. 2019, 141, 12388–12396; 31280562 10.1021/jacs.9b05850 PMC6687549

[27d] Y. Hu , K. Lang , C. Li , J. B. Gill , I. Kim , H. Lu , K. B. Fields , M. K. Marshall , Q. Cheng , X. Cui , L. Wojtas , X. P. Zhang , J. Am. Chem. Soc. 2019, 141, 18160–18169. 31622088 10.1021/jacs.9b08894 PMC6854306

