# Supplementary material for: Design Platform for Sustainable Catalysis with Radicals: Electrochemical Activation of Cp2TiCl2 for Catalysis Unveiled
Source: Chemistry. 2021 Jan 12;27(15):4903–12. doi: 10.1002/chem.202004519 (PMC7986168; doi:10.1002/chem.202004519)
Supplement: Supplementary file 1 — Supplementary [file CHEM-27-4903-s001.pdf]

Chemistry–A European Journal

Supporting Information

Design Platform for Sustainable Catalysis with Radicals: Electrochemical Activation of Cp_2TiCl_2 for Catalysis Unveiled

Tobias Hilche,[a] Philip H. Reinsberg,[b] Sven Klare,[a] Theresa Liedtke,[a] Luise Schäfer,[a] and Andreas Gansäuer*[a]

Table of Contents

1. General Information 3

2. Synthesis of Substrates 3

   2.1 Synthesis of *N*,*N'*-(1,2-phenylene)bis(3,5-bis(trifluoromethyl)-benzenesulfonamide) L3 3

3. Cyclic Voltammetry Experiments 5

   3.1 General Information 5

   3.2 General Procedure for Cyclic Voltammetry Experiments 5

   3.3 CVs of the Additives L1 – L3 6

4. Bulk Electrolysis 9

   4.1 Controlled-Potential Electrolysis in THF: 9

5. Catalytic Radical Arylation 10

   5.1 General Procedure for the Arylation of Epoxides (GP 1): 10

   5.2 Synthesis of (3-methyl-1-phenyl-2,3-dihydro-1-H-indol-3-yl)methanol 2 10

6. Rotating Ring-Disk Electrode Experiments 12

   6.1 General Remarks 12

   6.2 Thiourea L1 15

   6.3 Sulfonamide L3 20

   6.4 Squaramide L2 24

   6.5 Comparison 28

7. Density Functional Theory Calculations 29

   7.1 General Information 29

   7.2 Calculated Structures 30

8. References 33

9. Structural Data (xyz-Format) 35

1. General Information

All reactions involving air- or moisture sensitive compounds were prepared in oven dried glassware under inert atmosphere (Ar) using standard Schlenk and vacuum line technique. All chemicals were purchased from *abcr GmbH, TCI GmbH, Alfa Aesar, Fluka, Acros, Flourochem* or *Sigma Aldrich* and were used without further purification. Solvents used in manipulations under inert atmosphere were either dried and deoxygenated by distillation (THF over Na/K-alloy) before use or purified inside a *M-Braun MB-SPS-800* solvent purification system and used after degasification. The NMR analysis was carried out on a *Bruker DPX 300 MHz*, *Bruker 400 MHz* or *Bruker 500 MHz* spectrometer. ^{1}H and ^{13}C NMR chemical shifts were specified in ppm and calibrated by using the residual undeuterated solvent as internal reference [^{1}H NMR: $CHCl_3$ (7.26 ppm), C_6HD_5 (7.16 ppm), DMSO-d_5 (2.50 ppm), CHD_2CN (1.94 ppm), THF-d_7 (1.72 ppm, 3.58 ppm); ^{13}C NMR: $CDCl_3$ (77.0 ppm), C_6D_6 (128.0 ppm), DMSO-d_6 (39.5 ppm), CD_3CN (118.3 ppm), THF-d_8 (25.3 ppm, 67.2 ppm)]. High resolution mass spectra analysis was measured on a *Thermoquest MAT 95 CL* or *Thermo Fisher Scientific LTQ Orbitrap XL* instrument. IR spectra were obtained on an ATR-IR Spectrometer *Thermo Electron Nicolet™ 380* instrument as neat film. CHNS analysis was measured on an *Elementar Analysesysteme varioMICRO* instrument. Silica gel (230-400 mesh) supplied by *Merck* and *Macherey-Nagel* or neutral aluminum oxide 90 supplied by *Merck* was used as stationary phase for column chromatography.

2. Synthesis of Substrates

The substrate **1** was prepared according to the literature procedure. The additives **L1**[S1] and **L2**[S2] were synthesized following the cited literature. Analytical data of the corresponding products can also be found in these references. **L3** was synthesized in analogy to the cited literature.[S3]

2.1 Synthesis of *N,N'*-(1,2-phenylene)bis(3,5-bis(trifluoromethyl)-benzenesulfonamide) L3

ortho-Phenylenediamine (0.98 g, 9.0 mmol, 1.0 eq) was dissolved in dry pyridine (15 mL) and the mixture was cooled to 0 °C. Then, 3,5-bis(trifluoromethyl)benzenesulfonyl chloride (5.63 g, 18.0 mmol, 2.0 eq.) was added. After 30 min the cooling bath was removed and the reaction stirred at room temperature for 19 h. Water (200 mL) was added and the mixture was heated to 45 °C for 1 h. The solvent was removed under reduced pressure at 50 °C. The crude product was recrystallised from EtOH/H_2O 4:1 and dried *in vacuo* at 100 °C. The product was obtained as a slightly red solid (4.85 g, 7.35 mmol, 82%).

^{1}H NMR (500 MHz, THF-d8, 298 K): δ [ppm] = 8.94 (s, 2H), 8.32 (s, 2H), 8.18 (s, 4H), 7.10 (m, 2H), 6.99 (m, 2H).

^{13}C NMR (125 MHz, THF-d8, 298 K): δ [ppm] = 143.3 (2C), 133.4 (q, $^{2}J_{F,C}$ = 34.2 Hz, 4C), 132.3 (2C), 129.0 (4C), 128.5 (2C), 127.8 (2C), 126.7 (2C), 123.9 (q, $^{1}J_{C,F}$ = 237.0 Hz, 4C).
^{19}F NMR (470 MHz, THF-d8, 298 K): δ [ppm] = −63.83 (12F).
IR (pure, ATR): $\tilde{\nu}$ [cm^{-1}] = 3230 (m), 3106 (w), 1736 (w), 1348 (s), 1320 (s), 1278 (s), 1104 (s), 904 (s), 723 (s).
Mp.: 210 – 212 °C.
HR MS (ESI) [M–H]$^{-}$: *m/z* calc. for $C_{22}H_{11}F_{12}N_2O_4S_2$: 658.9974, found: 658.9974.
CHNS calc. for $C_{22}H_{11}F_{12}N_2O_4S_2$: C: 40.01, H: 1.83, N: 4.24, S: 9.71; found: C: 40.14, H: 2.03, N: 4.24, S: 9.89.

The data are in agreement with the literature.[S4]

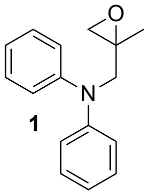

1

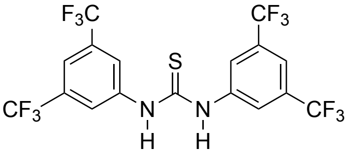

L1

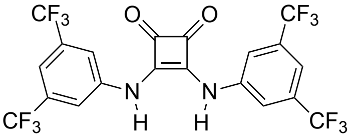

L2

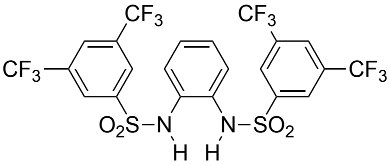

L3

3. Cyclic Voltammetry Experiments

3.1 General Information

The chemicals tetrabutylammonium hexafluorophosphate (NBu_4PF_6), tetrabutylammonium chloride (NBu_4Cl) and $AgNO_3$ were purchased in electrochemical grade from *Aldrich* and stored in a glovebox under an inert atmosphere (Ar).

All cyclic voltammetry experiments were carried out in a glovebox and were performed by a 1400D Electrochemical Analyzer (CH-Instruments). A glassy carbon disk of 1 mm diameter was used as working electrode material. The surface of the working electrode was polished with diamond paste (0.25 µm) provided by Struers followed by cleaning in an ethanol bath. A platinum coil melted into glass served as the counter electrode and the reference electrode consists of a silver wire immersed in a Pyrex tube containing NBu_4PF_6 (0.2 M) and NBu_4I (0.02 M) in THF separated from the main solution by a ceramic frit. The potentials were referenced against the Fc^+/Fc redox couple (the addition of 0.52 V to the potential will lead to the values of a SCE in 0.2 M NBu_4PF_6/THF). The iR compensation mode of the CH-Instrument Electrochemical Analyzer was used for all cyclic voltammetry experiments.

3.2 General Procedure for Cyclic Voltammetry Experiments

The conducting salt NBu_4PF_6 (0.775 g, 0.200 mmol) was dissolved in the freshly distilled solvent (10 mL) and a magnetic stir bar was added to an oven dried cyclic voltammetry cell. Background measurements were conducted to reduce coulomb currents in the analysis by subtraction from the CVs recorded with analyte. Titanocene dichloride (5 mg, 0.02 mmol) was added to the cyclic voltammetry cell and CVs were recorded. After this, the indicated amount of additive was added. The cyclic voltammetry experiment was carried out at different sweep rates (0.05 Vs^{-1}, 0.1 Vs^{-1}, 0.2 Vs^{-1}, 0.5 Vs^{-1}, 1 Vs^{-1}, 2 Vs^{-1}, 5 Vs^{-1}, 10 Vs^{-1}, 20 Vs^{-1}, 50 Vs^{-1}) and the solution was stirred before measuring the respective sweep rate. At the end of the experiment a small amount of ferrocene (0.02 mmol) was added as an internal reference and the potential of the Fc^+/Fc redox couple was recorded.

3.3 CVs of the Additives L1 – L3

Here, the CVs of **L1** – **L3** at 0.2 and 2 Vs^{-1} are depicted for reference:

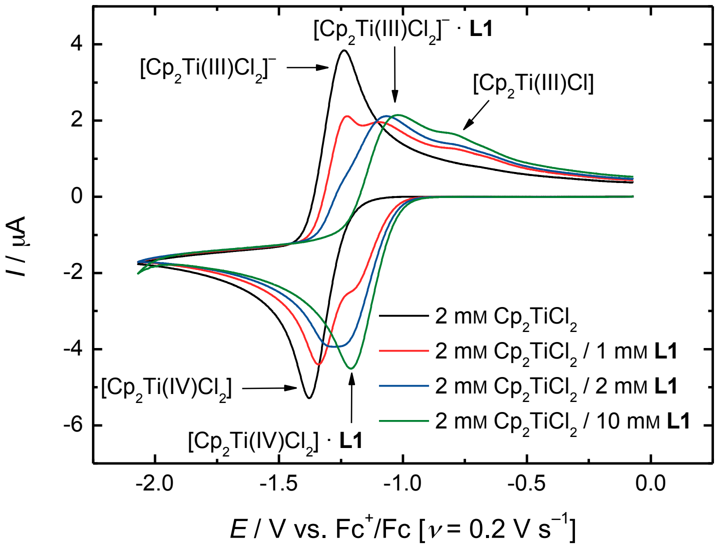

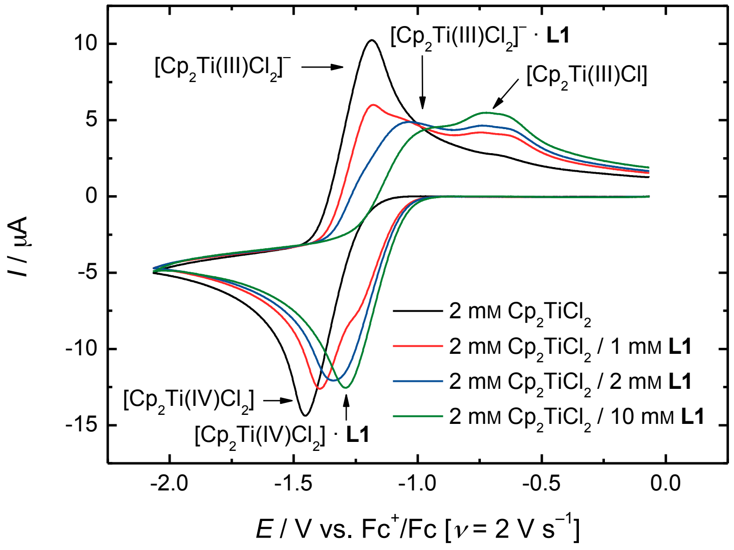

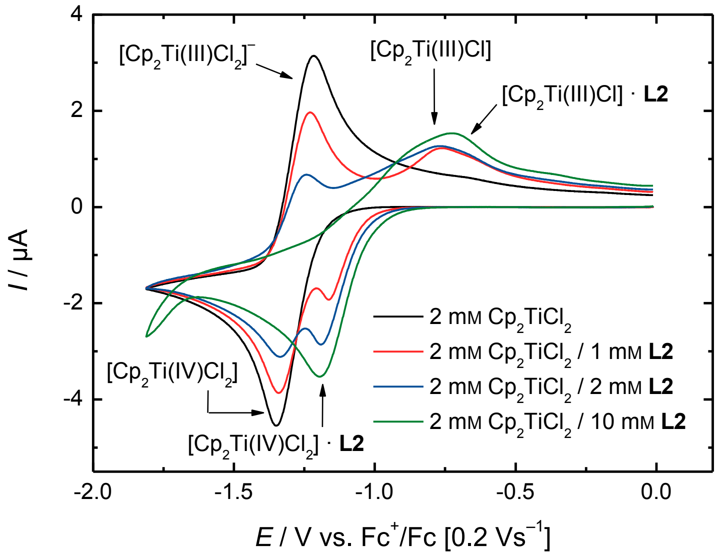
[Cp2Ti(III)Cl2]–
[Cp2Ti(III)Cl]
[Cp2Ti(III)Cl] · L2
[Cp2Ti(IV)Cl2]
[Cp2Ti(IV)Cl2] · L2
2 mM Cp2TiCl2
2 mM Cp2TiCl2 / 1 mM L2
2 mM Cp2TiCl2 / 2 mM L2
2 mM Cp2TiCl2 / 10 mM L2
I / µA
E / V vs. Fc+/Fc [0.2 Vs–1]

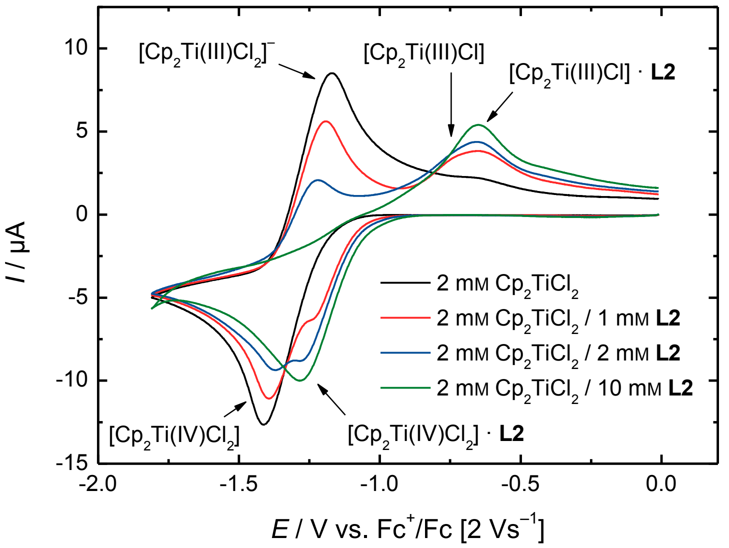
[Cp2Ti(III)Cl2]–
[Cp2Ti(III)Cl]
[Cp2Ti(III)Cl] · L2
[Cp2Ti(IV)Cl2]
[Cp2Ti(IV)Cl2] · L2
2 mM Cp2TiCl2
2 mM Cp2TiCl2 / 1 mM L2
2 mM Cp2TiCl2 / 2 mM L2
2 mM Cp2TiCl2 / 10 mM L2
I / µA
E / V vs. Fc+/Fc [2 Vs–1]

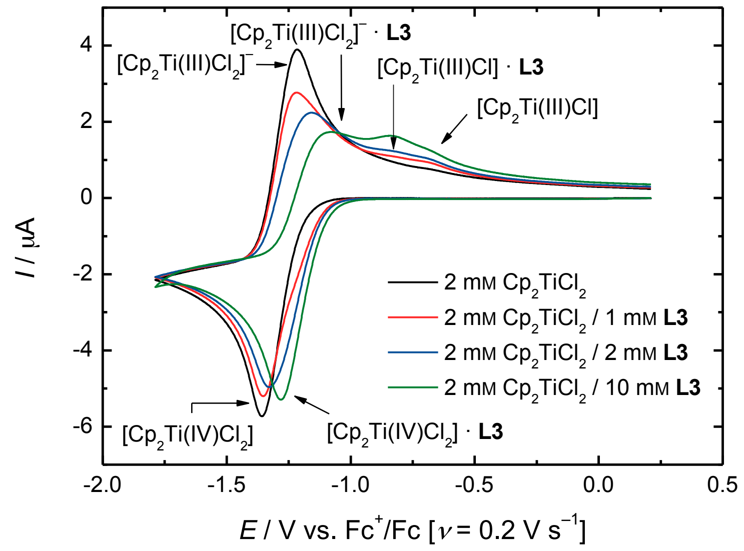
[Cp$_2$Ti(III)Cl$_2$]$^-$ · **L3**
[Cp$_2$Ti(III)Cl$_2$]$^-$
[Cp$_2$Ti(III)Cl] · **L3**
[Cp$_2$Ti(III)Cl]
2 mM Cp$_2$TiCl$_2$
2 mM Cp$_2$TiCl$_2$ / 1 mM **L3**
2 mM Cp$_2$TiCl$_2$ / 2 mM **L3**
2 mM Cp$_2$TiCl$_2$ / 10 mM **L3**
[Cp$_2$Ti(IV)Cl$_2$]
[Cp$_2$Ti(IV)Cl$_2$] · **L3**
I / μA
E / V vs. Fc$^+$/Fc [ν = 0.2 V s^{-1}]
4
2
0
-2
-4
-6
-2.0
-1.5
-1.0
-0.5
0.0
0.5

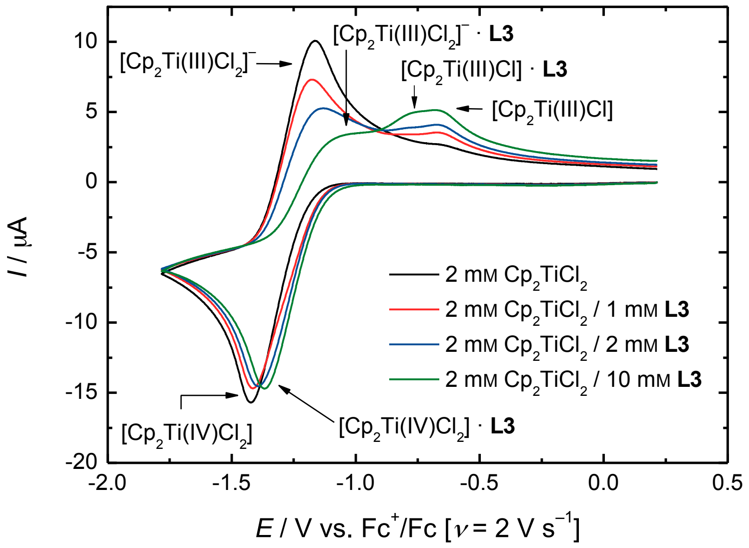
[Cp$_2$Ti(III)Cl$_2$]$^-$ · **L3**
[Cp$_2$Ti(III)Cl$_2$]$^-$
[Cp$_2$Ti(III)Cl] · **L3**
[Cp$_2$Ti(III)Cl]
2 mM Cp$_2$TiCl$_2$
2 mM Cp$_2$TiCl$_2$ / 1 mM **L3**
2 mM Cp$_2$TiCl$_2$ / 2 mM **L3**
2 mM Cp$_2$TiCl$_2$ / 10 mM **L3**
[Cp$_2$Ti(IV)Cl$_2$]
[Cp$_2$Ti(IV)Cl$_2$] · **L3**
I / μA
E / V vs. Fc$^+$/Fc [ν = 2 V s^{-1}]
10
5
0
-5
-10
-15
-20
-2.0
-1.5
-1.0
-0.5
0.0
0.5

4. Bulk Electrolysis

The divided bulk electrolysis cell was purchased from *ALS* which is distributed by *C3 Prozess- und Analysentechnik GmbH* in Germany. All controlled potential electrolysis experiments were carried out in a glovebox and were performed by a 1400D Electrochemical Analyzer (CH-Instruments). A glassy carbon mesh electrode was used as the cathode and a platinum wire immersed into a solution of CH_3CN/NBu_4PF_6 (0.2 M) and Cp_2TiCl_2 (0.01 M) served as anode. The reference electrode is composed of a silver wire and a solution of NBu_4PF_6 (0.1 M) and $AgNO_3$ (0.01 M) in CH_3CN.

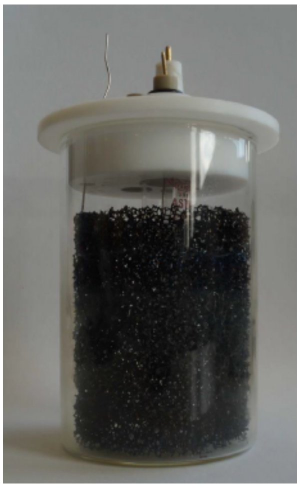

Figure S1: Bulk Electrolysis Cell used for catalyst activation.

4.1 Controlled-Potential Electrolysis in THF:

The bulk electrolysis cell (100 mL) was equipped with a stirring bar and NBu_4PF_6 (4.65 g, 12.0 mmol), Cp_2TiCl_2 (149 mg, 0.60 mmol), additive (**L1**: 300 mg, 0.60 mmol, CPE1; **L2**: 342 mg, 0.60 mmol, CPE2; **L3**: 396 mg, 0.60 mmol, CPE3) and THF (60 mL) were added inside a glovebox. The electrodes were put inside the cell and connected to the potentiostat. The electrolysis was conducted at a controlled potential (E = –1.4 V), during the experiment the stirring was set to 260 rpm and the current flow was monitored. As an indicator the color change from red (Ti^{IV}) to green (Ti^{III}) was used and the electrochemical reduction was stopped when the current flow was below 1 mA. The electrolyzed solution was used as is for the arylation.

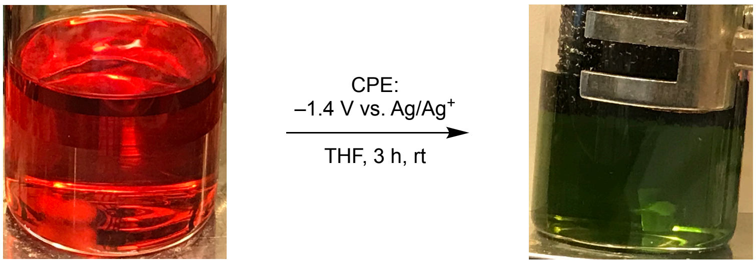

Figure S2: Exemplary Electrolysis of Cp_2TiCl_2 and **L2** in THF.

5. Catalytic Radical Arylation

5.1 General Procedure for the Arylation of Epoxides (GP 1):

A pressure stable Schlenk flask was filled with a stirring bar and the epoxide (1.0 eq.). The solution (**CPE 1 to CPE3**) containing the electrochemically generated [$Cp_2Ti(III)Cl$]-catalyst (0.01 M, 0.1 eq.) was added inside a glovebox. After that, the reaction mixture was stirred for the indicated time at the indicated temperature. The reaction mixture was allowed to cool down to ambient temperature, the solvent was evaporated under reduced pressure and the crude product was purified by column chromatography.

5.2 Synthesis of (3-methyl-1-phenyl-2,3-dihydro-1-H-indol-3-yl)methanol 2

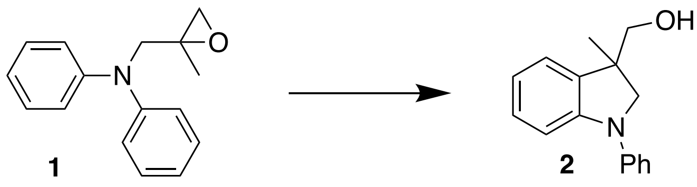

Following **GP 1** epoxide **1** (120 mg, 0.5 mmol, 1.0 eq.) and the ‚Cp_2TiCl/THF'-catalyst system (**CPE 1**, 5 mL, 0.1 eq.) were stirred for 2 h at 90 °C (oil bath temperature). The crude product was purified by column chromatography (SiO_2, CH:EA 9:1, R_f = 0.24) and the product **2** (71 mg, 0.30 mmol, 59%) was obtained as a colorless oil.

Following **GP 1** epoxide **1** (120 mg, 0.5 mmol, 1.0 eq.) and the ‚Cp_2TiCl/THF'-catalyst system (**CPE 2**, 5 mL, 0.1 eq.) were stirred for 2 h at 75 °C (oil bath temperature). The crude product was purified by column chromatography (Al_2O_3, CH:EA 8:2) and the product **2** (104 mg, 0.43 mmol, 87%) was obtained as a colorless oil.

Following **GP 1** epoxide **1** (120 mg, 0.5 mmol, 1.0 eq.) and the ‚Cp_2TiCl/THF'-catalyst system (**CPE 3**, 5 mL, 0.1 eq.) were stirred for 2 h at 75 °C (oil bath temperature). The crude product was purified by column chromatography (Al_2O_3, CH:EA 8:2) and the product **2** (106 mg, 0.44 mmol, 88%) was obtained as a colorless oil.

Following **GP 1** epoxide **1** (120 mg, 0.5 mmol, 1.0 eq.) and the ‚Cp_2TiCl/THF'-catalyst system (**CPE 3**, 5 mL, 0.1 eq.) were stirred for 4 h at 75 °C (oil bath temperature). The crude product was purified by column chromatography (Al_2O_3, CH:EA 8:2) and the product **2** (111 mg, 0.46 mmol, 93%) was obtained as a colorless oil.

Following **GP 1** epoxide **1** (120 mg, 0.5 mmol, 1.0 eq.) and the ‚Cp_2TiCl/THF'-catalyst system (**CPE 3**, 5 mL, 0.1 eq.) were stirred for 5 h at 75 °C (oil bath temperature). The crude product was purified by column chromatography (Al_2O_3, CH:EA 8:2) and the product **2** (110 mg, 0.46 mmol, 92%) was obtained as a colorless oil.

^{1}H NMR (500 MHz, C_6D_6, 298 K): δ [ppm] = 7.23 – 7.16 (m, 2H), 7.16 – 7.09 (m, 2H), 7.02 (ddd, *J* = 8.0, 7.4, 1.4 Hz, 1H), 6.93 (ddd, *J* = 7.3, 1.4, 0.6 Hz, 1H), 6.89 (tt, *J* = 7.3, 1.2 Hz, 1H), 6.75 (td, *J* = 7.4, 1.0 Hz, 1H), 3.62 (d, *J* = 9.3 Hz, 1H), 3.31 – 3.27 (m, 2H), 3.21 (d, *J* = 10.5 Hz, 1H), 1.14 (s, 3H), 0.94 (s, 1H).
^{13}C NMR (126 MHz, C_6D_6, 298 K): δ [ppm] = 147.1, 144.5, 136.3, 129.5, 123.6, 121.2, 119.4, 118.0, 108.9, 68.9, 61.8, 45.5, 22.1.

The data are in agreement with the literature.[S5]

6. Rotating Ring-Disk Electrode Experiments

Here, a more detailed discussion of the RRDE experiments is included. The discussion is started with measurements of the base electrolyte (2 mM Cp_2TiCl_2 and 0.2 M NBu_4PF_6 ($TBAPF_6$) in THF) and will continue with **L1**, **L3** and **L2** according to the increasing complexity of the systems.

6.1 General Remarks

In cyclic voltammetry[S6] mass transport is only given by diffusion. This is why detected currents for an electron transfer event run through a peak value at a potential, where the concentration of the analyte at the surface is close to zero and the flux of species towards the electrode is at maximum. The following decrease of current density is a result of the diffusion layer extending into the bulk electrolyte, which leads to a decreasing concentration gradient. With a rotating electrode like a rotating disk or ring-disk electrode[S7] a continuous flow carries fresh bulk solution in vertical direction toward the electrode surface. However, close to the surface, the velocity of the electrolyte moving orthogonal to the electrode decreases to zero and changes into a radial flux due to the rotation of the electrode. Thus, a thin layer of electrolyte with a constant thickness is established in front of the electrode, through which the analyte has to diffuse. At the boundary to the bulk solution, the analyte concentration always equals the bulk concentration. As a consequence, a plateau rather than a peak is observed in a CV experiment at an RRDE once the concentration of the analyte at the surface of the electrode is close to zero. This is the diffusion limited current I_{lim}. In a four-electrode set-up, with a disk and a ring electrode around it, the species formed in the original redox event at the disk or in a fast follow-up reaction are transported to the ring and can be detected. By adjusting the constant potential at the ring electrode, different species can be discerned.

This feature of the RRDE makes the method perfectly suitable for our aim to analyze the different species already observed in CV measurements. All measurements were conducted at a sweep rate of 25 mV s^{-1} with a reductive sweep followed by an oxidative back-sweep. Each measurement was performed at four rotation frequencies f (4, 9, 16 and 25 Hz). A thin-gap glassy carbon disk and ring (AFE7R9GCGC, *Pine Research*) with a disk surface area of A_{Disk} = 0.247 cm² and a theoretical collection efficiency of N_0 = 0.37 was used as the working electrode. In order to put our later results into relation, we started with a measurement of Cp_2TiCl_2 in 0.2 M NBu_4PF_6/THF, which is the base electrolyte. This system was already studied in depth in the Daasbjerg group.[S8] The ring potential was set to 0.05 and 0.66 V vs. Ag/Ag^+. The voltammograms are shown in Figure S3. Note that the disk potential has been iR-corrected. The uncompensated resistance was determined via electrochemical impedance spectroscopy and was in the range of 500 Ω. Note that the iR-correction does not severely influence the evaluation of the equilibrium constants as the constants are evaluated from diffusion-limited currents and transfer ratios.

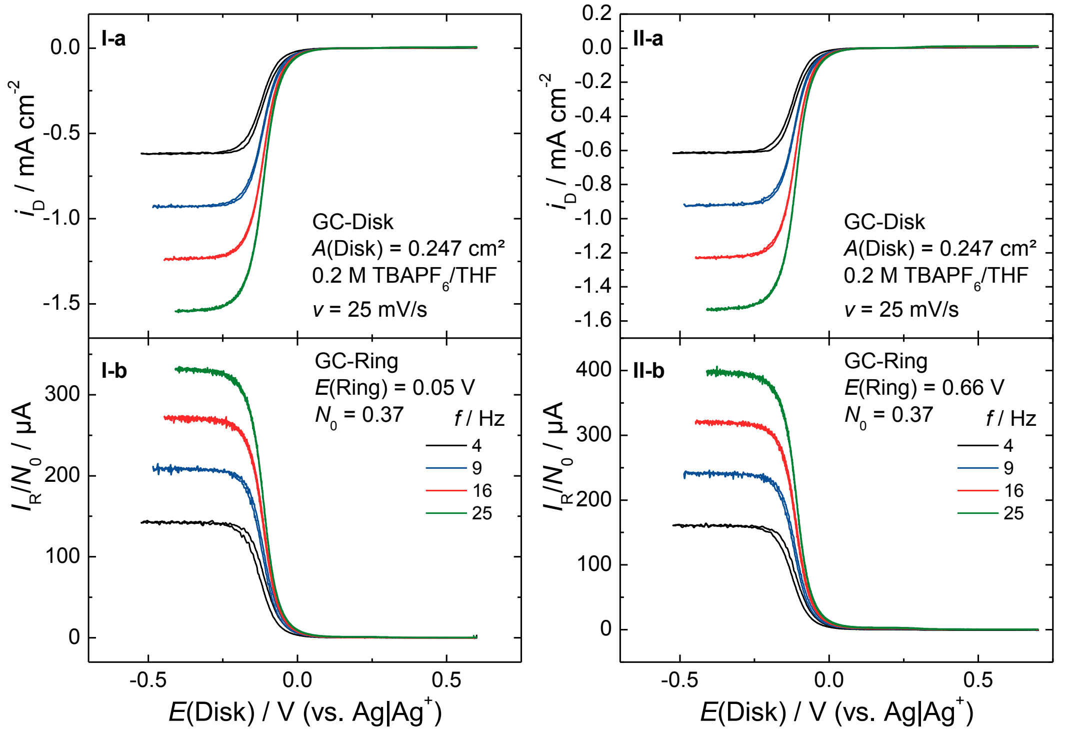

Figure S3. Disk current densities i_D (a) and normalized ring currents I_R/N_0 (b) of the RRDE measurements of a solution of 2 mM Cp_2TiCl_2 in 0.2 M $TBAPF_6$/THF with a ring potential of 0.05 V (I) and 0.66 V (II) at different rotation frequencies. The disk potential has been iR-corrected.

According to the E_qC_r mechanism,[S9] the reduced titanocene $[Cp_2Ti(III)Cl_2]^-$ is in an equilibrium with $[Cp_2Ti(III)Cl]$ and Cl^- (K_2*, (1)).

$$[Cp_2Ti(III)Cl_2]^{\ominus} \xrightleftharpoons{K_2^*} [Cp_2Ti(III)Cl] + Cl^{\ominus} \qquad (1)$$

From the change of the coordination number at Ti results a significant difference of the redoxpotential of the two species. At a ring potential of 0.05 V only $[Cp_2Ti(III)Cl_2]^-$, directly formed in the electron transfer event at the disk electrode, can be detected at the ring. The cleaving product of the reversible follow up reaction, $[Cp_2Ti(III)Cl]$, is only detected at 0.66 V (see the CVs in section 3.3). Therefore, the ratio of ring and disk currents I_R/I_D is higher at 0.66 V than at 0.05 V as both Ti(III) species are detected at the higher potential. Not all reduction products formed at the disk reach the ring electrode and can be detected. A part of the products diffuses into the bulk solution instead. The fraction that is detected at the ring electrode is a number specific for the ring-disk electrode and is called theoretical collection efficiency (N_0). If I_R/I_D is normalized by N_0, the transfer ratio is obtained. At 0.66 V all species are reoxidized and $I_R/I_D/N_0$ should be equal to 1. The transfer ratios of the Cp_2TiCl_2 solution are depicted in Figure S4.

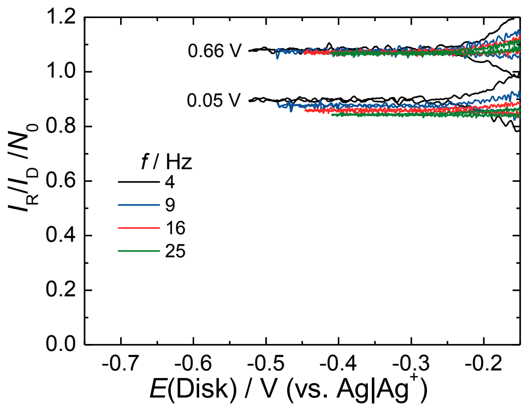

Figure S4. Transfer ratios $I_R/I_D/N_0$ of 2 mM Cp_2TiCl_2 in 0.2 M $TBAPF_6$/THF.

The transfer ratios recorded at 25 Hz (0.845 at 0.05 V and 1.068 at 0.66 V) give access to the dissociation equilibrium K_2* of $[Cp_2Ti(III)Cl_2]^-$ to form [Cp_2Ti(III)Cl] and Cl^-: At 0.05 V the concentration of $[Cp_2Ti(III)Cl_2]^-$ can directly be obtained by multiplication of the initial Ti concentration with the transfer ratio given that the rate constant of the dissociation-association equilibrium is small as compared to the residence time of the species at the ring electrode. Otherwise, additional $[Cp_2Ti(III)Cl_2]^-$ would be generated from the association reaction between [Cp_2Ti(III)Cl] and Cl^-: Therefore the measurement at 25 Hz was used for evaluation, where the transport of the species over the ring is the fastest. Alternatively, the transfer ratio could be extrapolated to an infinite rotation rate (e.g. vs. f^{-1}) to minimize the effect of the association reaction. However, due to experimental uncertainty as well as the lack of a suitable model for fitting, the extrapolation to infinite rotation rate would also pose a large uncertainty on final concentration. Having determined the concentration of $[Cp_2Ti(III)Cl_2]^-$, the concentration of [Cp_2Ti(III)Cl] can be extracted from the transfer ratio at 0.66 V. At 0.66 V both species can be oxidized and thus, the transfer ratio corresponds to the sum of the concentrations of [Cp_2Ti(III)Cl] and $[Cp_2Ti(III)Cl_2]^-$. The ratio of both species is given by (2). K_2* in the base electrolyte amounts to 0.11 mM.

$$\frac{c([\mathrm{Cp_2Ti(III)Cl_2}]^-)}{c([\mathrm{Cp_2Ti(III)Cl}])} = \frac{N(0.05\ \mathrm{V})}{N(0.66\ \mathrm{V}) - N(0.05\ \mathrm{V})} \qquad (2)$$

6.2 Thiourea L1

In the CVs of titanocene dichloride and thiourea **L1** a total of five species were observed. The Ti(IV) species [$Cp_2Ti(IV)Cl_2$] and [$Cp_2Ti(IV)Cl_2$]***L1** are detected on the reductive sweep and the Ti(III) species [$Cp_2Ti(III)Cl_2$]$^-$, [$Cp_2Ti(III)Cl_2$]$^-$***L1** and [$Cp_2Ti(III)Cl$] are detected on the reverse sweep. For these species several equilibrium reactions have to be considered:

$$[Cp_2Ti(IV)Cl_2] + \mathbf{L1} \underset{}{\overset{K_a}{\rightleftharpoons}} [Cp_2Ti(IV)Cl_2]^*\mathbf{L1} \qquad (3)$$

$$[Cp_2Ti(III)Cl_2]^{\ominus} + \mathbf{L1} \overset{K_1}{\rightleftharpoons} [Cp_2Ti(III)Cl_2]^*\mathbf{L1}^{\ominus} \qquad (4)$$

$$[Cp_2Ti(III)Cl_2]^*\mathbf{L1}^{\ominus} \overset{K_2}{\rightleftharpoons} [Cp_2Ti(III)Cl] + Cl^*\mathbf{L1}^{\ominus} \qquad (5)$$

Measurements at the RRDE of titanocene dichloride and different amounts of **L1** were performed in 0.2 M $TBAPF_6$/THF to determine the relative and absolute amounts of each species. The voltammograms of the 4 and 25 Hz measurements and the corresponding transfer ratios at 0.05, 0.30 and 0.55 V ring potential are depicted in Figures S5 and S6.

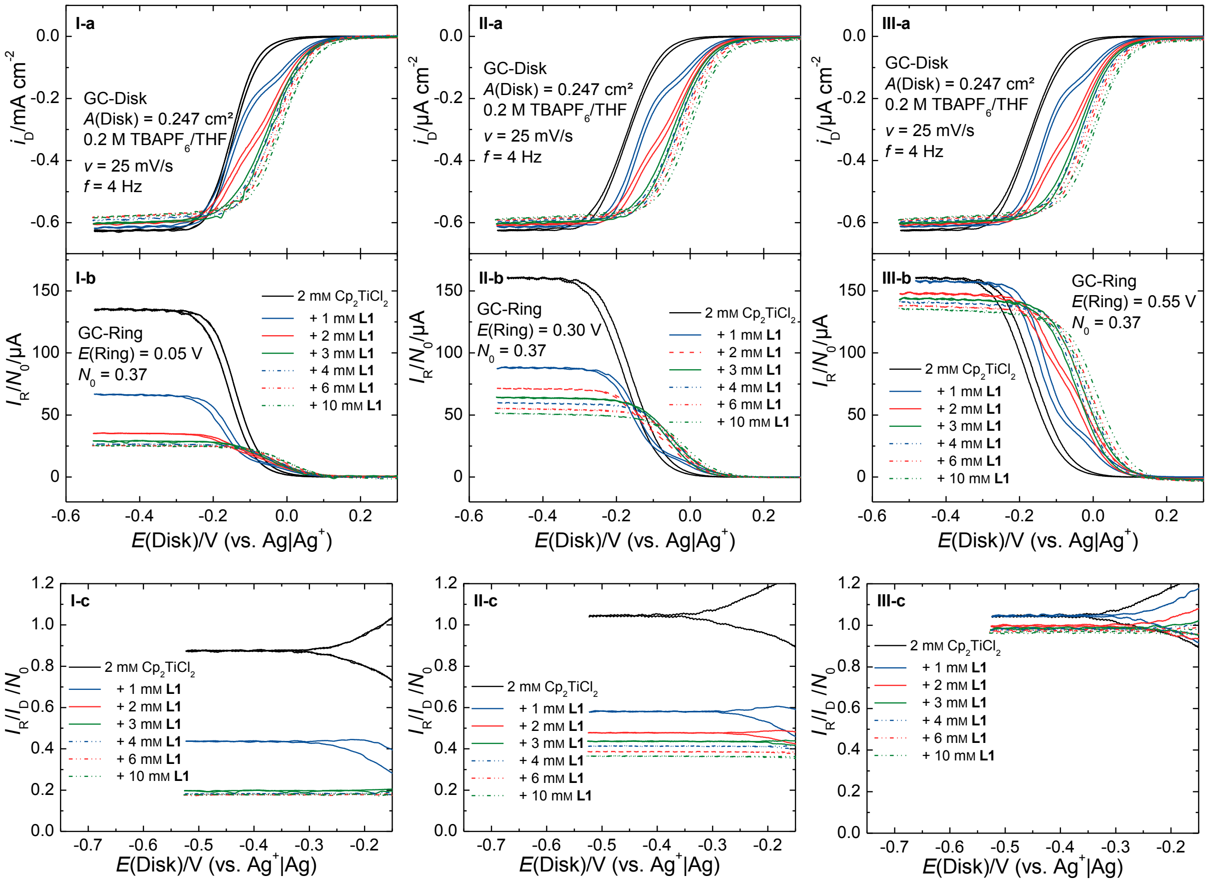

Figure S5: Disk current densities i_D (a), normalized ring currents I_R/N_0 (b) and transfer ratios $I_R/I_D/N_0$ (c) of the RRDE measurements of a solution of 2 mM Cp_2TiCl_2 in 0.2 M $TBAPF_6$/THF with different concentrations of **L1** at a ring potential of 0.05 (I), 0.30 (II) and 0.55 V (III) and a rotation frequency *f* of 4 Hz.

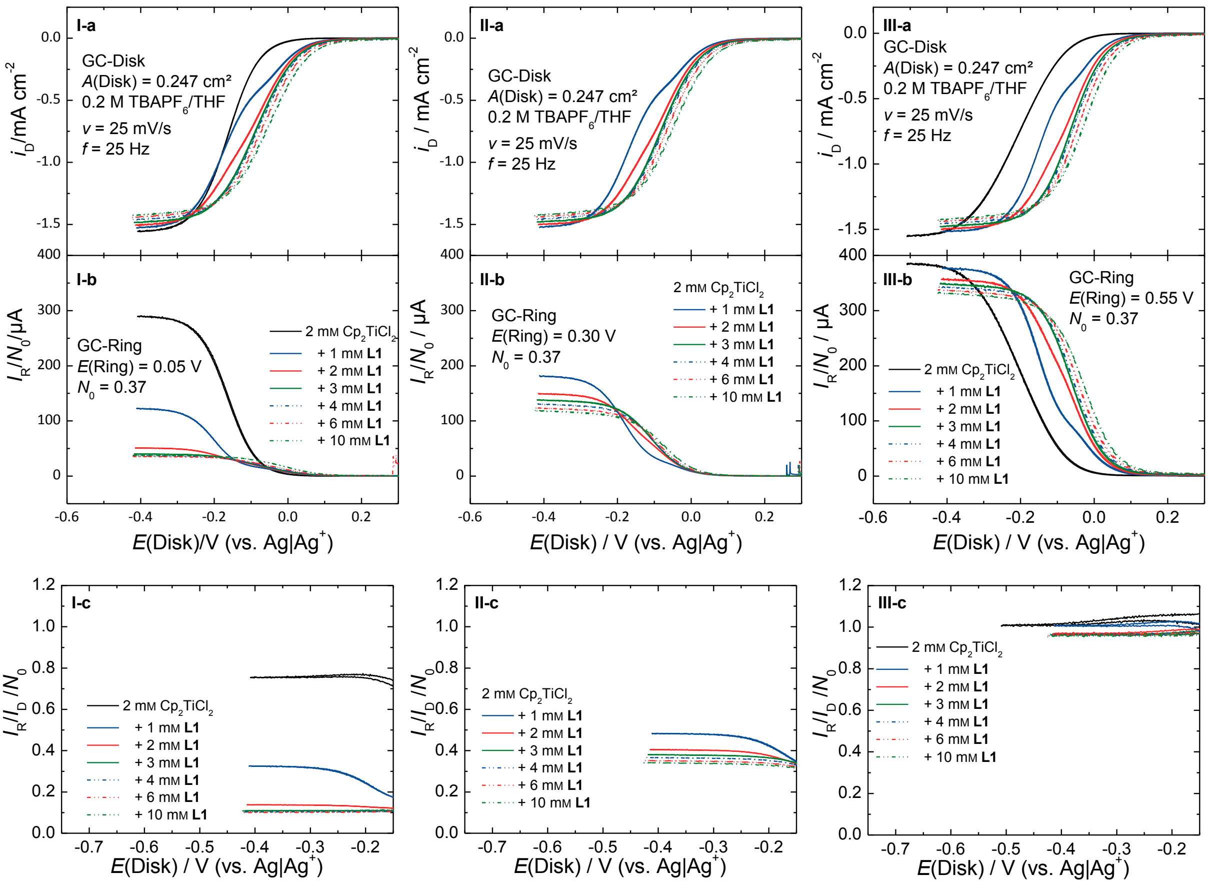

Figure S6: Disk current densities i_D (a), normalized ring currents I_R/N_0 (b) and transfer ratios $I_R/I_D/N_0$ (c) of the RRDE measurements of a solution of 2 mM Cp_2TiCl_2 in 0.2 M $TBAPF_6$/THF with different concentrations of **L1** at a ring potential of 0.05 (I), 0.30 (II) and 0.55 V (III) and a rotation frequency *f* of 25 Hz.

From the i_D voltammograms three important observations can be made each of which will be addressed below. I) The limiting current density i_{lim} at low potentials is decreasing in value with increasing concentration of **L1**. II) In the measurement with 1 mM of **L1** (0.5 equivalents) a shoulder in the current step is formed. III) The new reduction potential found after addition of **L1** is gradually shifting to more positive values with increasing concentration of **L1**.

The limiting current density at the disk electrode is related to the diffusion constant *D* of the analyte, the angular rotation frequency ω, the kinematic viscosity of the electrolyte ν and the bulk concentration of the analyte (*Levich* equation):[S10]

$$i_{lim,c} = -0.62nFAD_O^{2/3}\omega^{1/2}\nu^{-1/6}c_O^*. \qquad (6)$$

The other symbols have the usual meaning. The change of i_{lim} is an indication for the formation of a new species with a smaller diffusion coefficient, which is the $[Cp_2Ti(IV)Cl_2]$*L1 adduct. The gradual change of i_{lim} can be explained with the reversibility of the adduct formation (3). On its path to the disk electrode surface $[Cp_2Ti(IV)Cl_2]$ can associate to **L1** and dissociate again. The higher the concentration of **L1**, the larger is the diffusion length in the associated species. As a consequence, the

diffusion of [$Cp_2Ti(IV)Cl_2$]*$\mathbf{L1}$ is best reflected in the measurement with 10 mM of **L1**. In Figure S7 the diffusion limited currents at the disk for Cp_2TiCl_2 and with 10 mM and with 1 mM **L1** (here the shoulder current was taken) are plotted against the square root of the rotation frequency.

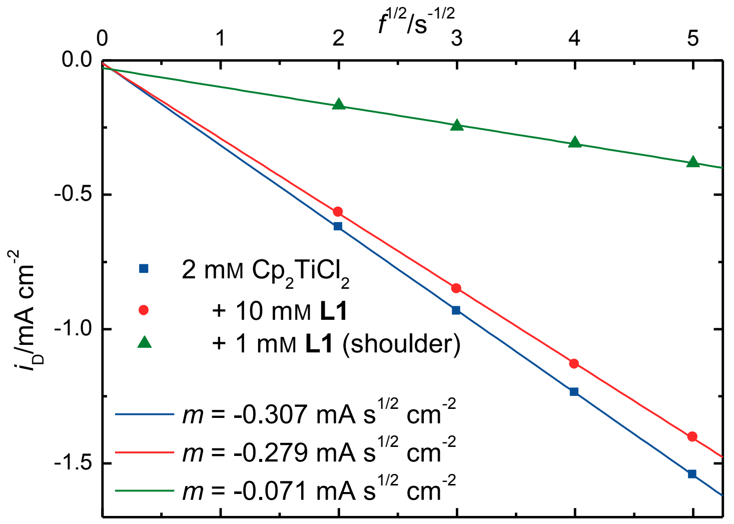

Figure S7. Diffusion limited current densities at the disk i_D as a function of $f^{1/2}$ for Cp_2TiCl_2 and with 10 mM of **L1** or 1 mM of **L1** (shoulder current). The slopes of the linear fits are given as m.

From the slopes m of a linear fit the ratio of the diffusion coefficients of [$Cp_2Ti(IV)Cl_2$] and [$Cp_2Ti(IV)Cl_2$]*$\mathbf{L1}$ can be obtained using (6). This ratio amounts to 1.15:

$$\frac{D([\mathrm{Cp_2Ti(IV)Cl_2}])}{D([\mathrm{Cp_2Ti(IV)Cl_2}]*\mathbf{L1})} = \left(\frac{m_{\mathrm{Diff}}([\mathrm{Cp_2Ti(IV)Cl_2}])}{m_{\mathrm{Diff}}([\mathrm{Cp_2Ti(IV)Cl_2}]*\mathbf{L1})}\right)^{\frac{3}{2}} \qquad (7)$$

The diffusion coefficient D([$Cp_2Ti(IV)Cl_2$]) for our system is not known in the literature. However, it can be easily calculated from the slope in Figure S7 (blue trace), if the kinematic viscosity v is known. Since v scales to the power of –1/6 (see (6)) it is acceptable to approximate this value by using v(THF) without conducting salt. From the literature a value of 5.2×10^{-3} cm^2 s^{-1} is obtained.[S11] Thus, 8.8×10^{-6} cm^2 s^{-1} results for D([$Cp_2Ti(IV)Cl_2$]) and 7.6×10^{-6} cm^2 s^{-1} for D([$Cp_2Ti(IV)Cl_2$]*$\mathbf{L1}$). The diffusion coefficient ratio together with the slopes of the base electrolyte and of the linear fit of the shoulder currents can be used to determine the equilibrium constant of association for (3) as the current – and as a result the slope m – in the shoulder is proportional to the concentration of the adduct in this equilibrium. The following relation is obtained:

$$\frac{c_0(\mathrm{Cp_2TiCl_2})}{c_{\mathrm{eq}}([\mathrm{Cp_2Ti(IV)Cl_2}]*\mathbf{L1})} = \frac{m_{\mathrm{Diff}}([\mathrm{Cp_2Ti(IV)Cl_2}]*\mathbf{L1})}{m_{\mathrm{shoulder}}([\mathrm{Cp_2Ti(IV)Cl_2}]*\mathbf{L1})} \qquad (8)$$

Here, c_0 denotes the initial concentration of Cp_2TiCl_2. K_a then amounts to 0.69 mM^{-1}.

The ring potential for the measurements with Cp_2TiCl_2 and **L1** was set to 0.05, 0.30 and 0.55 V vs. Ag/Ag^+. Analogous to before, the detection of $[Cp_2Ti(III)Cl_2]^-$, $[Cp_2Ti(III)Cl_2]^-$***L1** and $[Cp_2Ti(III)Cl]$ was achieved step-wise. The transfer ratios as a function of the concentration of **L1** are given in Figure S8.

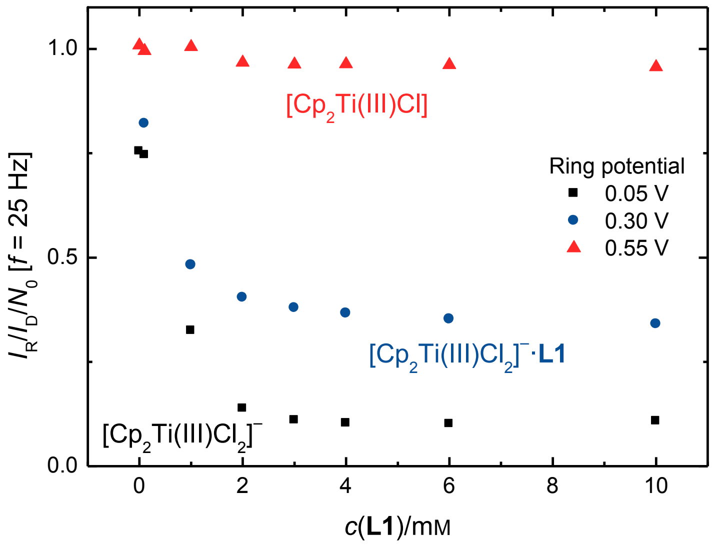

Figure S8. Transfer ratios $I_R/I_D/N_0$ for the measurements of Cp_2TiCl_2 and **L1** at different concentrations of **L1** at ring potentials of 0.05, 0.30 and 0.55 V and at a rotation frequency *f* of 25 Hz.

The transfer ratios yield the ratios of the equilibrium concentrations for equations (4) and (5).

$$\frac{c([\mathrm{Cp_2Ti(III)Cl_2}]^-)}{c([\mathrm{Cp_2Ti(III)Cl_2}]^-{*}\mathbf{L1})} = \frac{N(0.05\ \mathrm{V})}{N(0.30\ \mathrm{V}) - N(0.05\ \mathrm{V})} \tag{9}$$

$$\frac{c([\mathrm{Cp_2Ti(III)Cl_2}]^-{*}\mathbf{L1})}{c([\mathrm{Cp_2Ti(III)Cl}])} = \frac{N(0.30\ \mathrm{V}) - N(0.05\ \mathrm{V})}{N(0.5\ \mathrm{V}) - N(0.30\ \mathrm{V}) - N(0.05\ \mathrm{V})} \tag{10}$$

For the association of **L1** to the anionic Ti(III) species K_1 amounts to 2 mM^{-1} and for the cleaving of Cl^- ***L1** off $Cp_2TiCl_2^-$***L1** K_2 equals 2.8 mM.

The observed gradual shift in the reduction potential with increasing amount of **L1** gives insight into the overall mechanistics of the equilibria in both oxidation states of Ti. It implies that the Nernst equation[S12] for this redox process depends on the concentration of free thiourea.

$$E = E_0 - \frac{RT}{F}\ln c(\mathbf{L1})^p - \frac{RT}{F}\ln\left\{K'\frac{c([\mathrm{Cp_2Ti(III)Cl_2}]^-{*}\mathbf{L1}_p)}{c([\mathrm{Cp_2Ti(IV)Cl_2}]{*}\mathbf{L1}_p)}\right\} \tag{11}$$

K' is introduced as a constant factor to account for the complex mesh of equilibria. A plot (see Figure S9) of the half-wave potential $E_{1/2}$ as a function of the decadic logarithm of *c*(**L1**) shows a slope of roughly 60 mV dec^{-1} at all rotation frequencies.

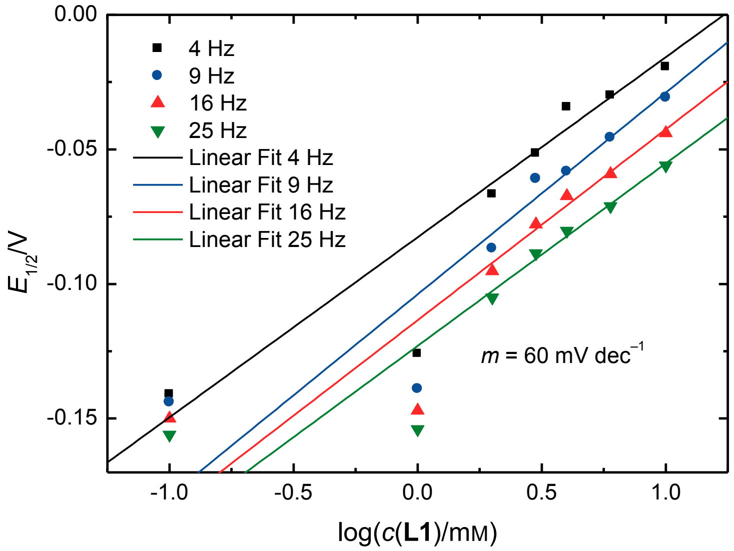

Figure S9. Plot of the half-wave potential $E_{1/2}$ of the RRDE measurements with Cp_2TiCl_2 and **L1**.

Therefore, *p* in (11) must be equal to 1, which is indicative of 1:1 complexation between **L1** and the titanocene species. With all experimental findings in mind we propose the electrochemical mesh-scheme shown in Scheme S1 for the Cp_2TiCl_2/**L1** couple.

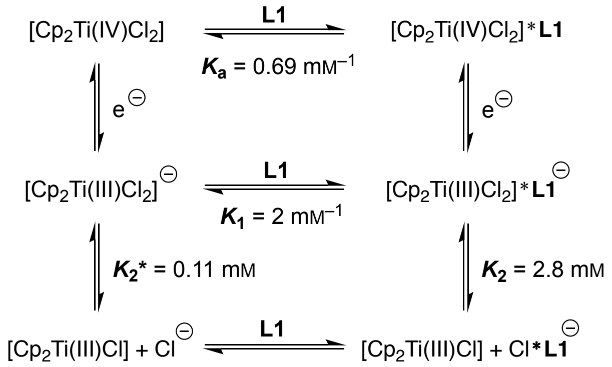

Scheme S1. Electrochemical mesh-scheme for the redox system of Cp_2TiCl_2 in the presence of **L1**.

It considers the E_qC_r mechanism of titanocene dichloride and adds the **L1** containing species. With the equilibrium constants we can quantify how effectively the C_r reaction (cleaving off of ‘Cl^-‘) is amplified in the presence of **L1** by comparing K_2 to K_2*. This gives a factor of 25.

6.3 Sulfonamide L3

In contrast to thiourea **L1** the sulfonamide **L3** shows a double peak in the oxidation wave of 'Cp$_2$TiCl'. This lead us to assume the presence of an adduct between the active species and **L3**, [Cp$_2$Ti(III)Cl]***L3**. Thus, the following equilibrium reactions have to be considered:

$$[\mathrm{Cp_2Ti(IV)Cl_2}] + \mathbf{L3} \xrightleftharpoons{K_a} [\mathrm{Cp_2Ti(IV)Cl_2}]^{*}\mathbf{L3} \qquad (12)$$

$$[\mathrm{Cp_2Ti(III)Cl_2}]^{\ominus} + \mathbf{L3} \xrightleftharpoons{K_1} [\mathrm{Cp_2Ti(III)Cl_2}]^{*}\mathbf{L3}^{\ominus} \qquad (13)$$

$$[\mathrm{Cp_2Ti(III)Cl_2}]^{*}\mathbf{L3}^{\ominus} \xrightleftharpoons{K_2} [\mathrm{Cp_2Ti(III)Cl}] + \mathrm{Cl}^{*}\mathbf{L3}^{\ominus} \qquad (14)$$

$$[\mathrm{Cp_2Ti(III)Cl}] + \mathbf{L3} \xrightleftharpoons{K_3} [\mathrm{Cp_2Ti(III)Cl}]^{*}\mathbf{L3} \qquad (15)$$

RRDE measurements with Cp$_2$TiCl$_2$ and **L3** were performed in the same manner as described for **L1**. The voltammograms of the 4 and 25 Hz measurements and the corresponding transfer ratios at 0.05, 0.23, 0.42 and 0.66 V ring potential are depicted in Figures S10 and S11.

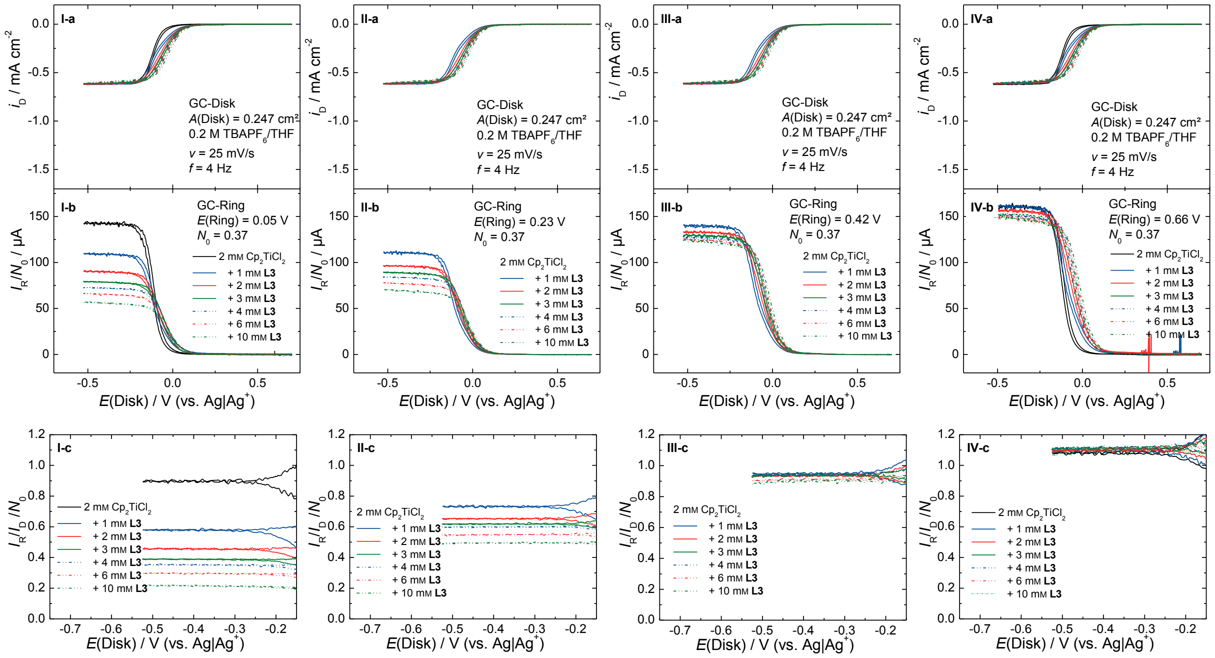

Figure S10: Disk current densities i_D (a), normalized ring currents I_R/N_0 (b) and transfer ratios $I_R/I_D/N_0$ (c) of the RRDE measurements of a solution of 2 mM Cp$_2$TiCl$_2$ in 0.2 M TBAPF$_6$/THF with different concentrations of **L3** at a ring potential of 0.05 (I), 0.23 (II), 0.42 (III) and 0.66 V (IV) and a rotation frequency f of 4 Hz.

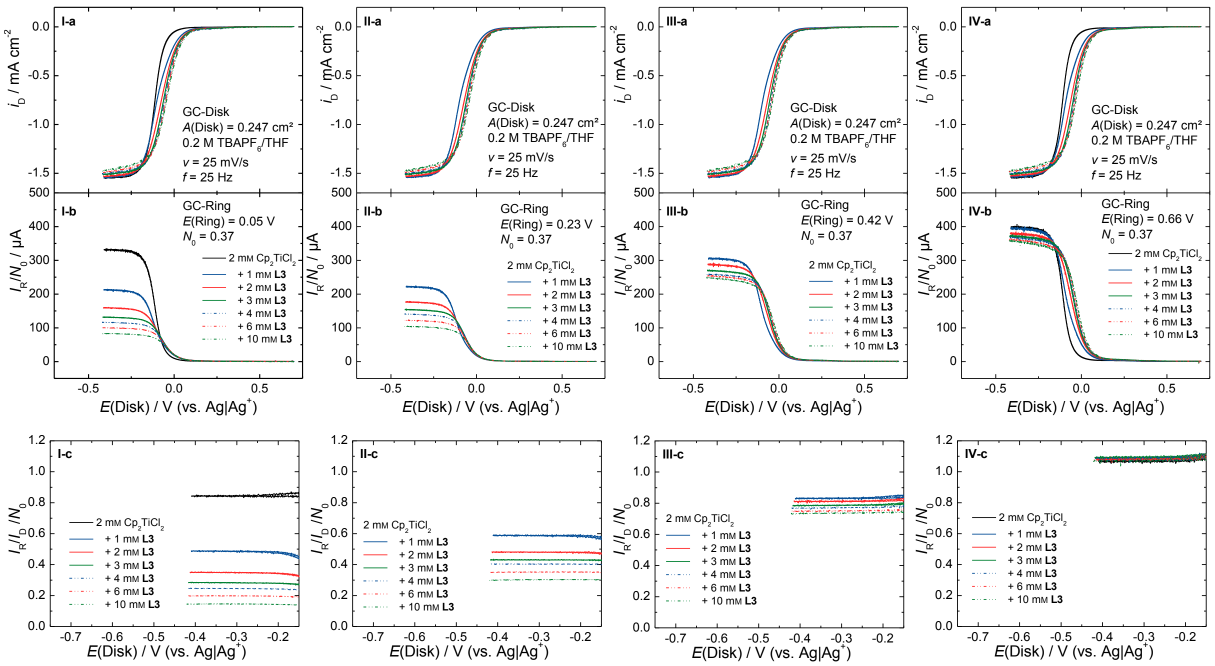

Figure S11: Disk current densities i_D (a), normalized ring currents I_R/N_0 (b) and transfer ratios $I_R/I_D/N_0$ (c) of the RRDE measurements of a solution of 2 mM Cp_2TiCl_2 in 0.2 M $TBAPF_6$/THF with different concentrations of **L3** at a ring potential of 0.05 (I), 0.23 (II), 0.42 (III) and 0.66 V (IV) and a rotation frequency *f* of 25 Hz.

Here, the same general observations can be made from the voltammograms at the disk as for **L1**. The current density i_D in the diffusion limited region is decreasing in value with increasing amount of **L3**, a shoulder at substoichiometric amounts of **L3** indicates the formation of the $[Cp_2Ti(IV)Cl_2]$***L3** adduct and a gradually shifting reduction potential with increasing concentration of **L3** is apparent. The diffusion limited current densities i_D of the base electrolyte containing Cp_2TiCl_2 and after addition of 10 mM of **L3** and the shoulder currents of the measurements with 1 mM of **L3** are plotted against $f^{1/2}$ in Figure S12.

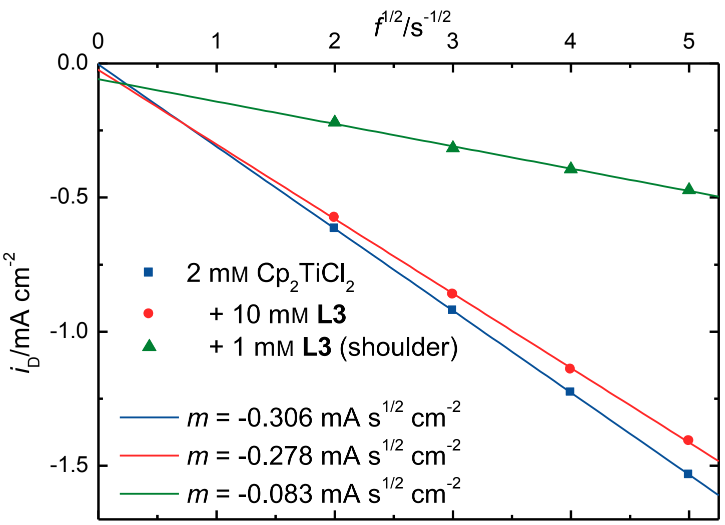

Figure S12. Diffusion limited current densities at the disk i_D as a function of $f^{1/2}$ for Cp_2TiCl_2 and with 10 mM of **L3** or 1 mM of **L3** (shoulder current). The slopes of the linear fits are given as *m*.

Using (7) the ratio of the diffusion coefficients of $[Cp_2Ti(IV)Cl_2]$ and its adduct with **L3** can be calculated to 1.15. Thus, 7.6×10^{-6} cm^2 s^{-1} results for $D([Cp_2Ti(IV)Cl_2]*$**L3**$)$. Accordingly, the equilibrium constant for (12), K_a, amounts to 1.07 mM^{-1} (compare (8)).

By setting the ring potential for the measurements to 0.05, 0.23, 0.42 and 0.66 V, all Ti(III) species could be detected as in the previous cases. The transfer ratios as a function of the concentration of **L3** are given in Figure S13.

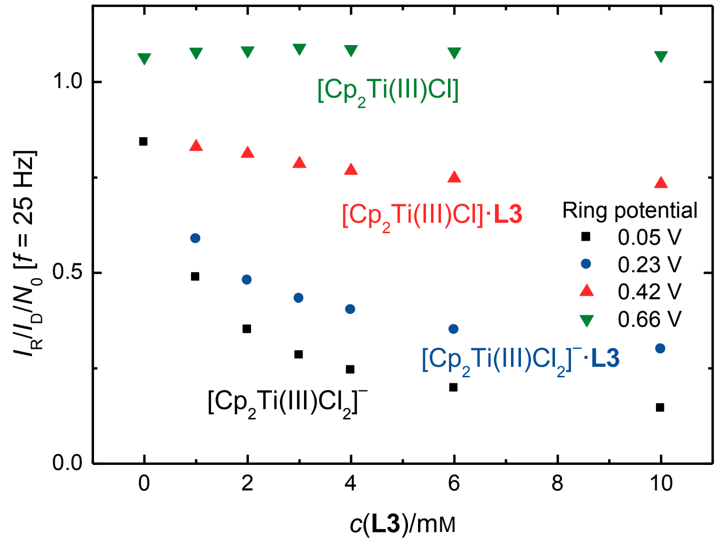

Figure S13. Transfer ratios $I_R/I_D/N_0$ for the measurements of Cp_2TiCl_2 and **L3** at different concentrations of **L3** at ring potentials of 0.05, 0.23, 0.42 and 0.66 V and at a rotation frequency *f* of 25 Hz.

The transfer ratios yield the equilibrium constants for (13) to (15) by following the same procedure as for **L1** (compare (9) and (10)). The adduct species $[Cp_2Ti(III)Cl]$***L3** is detected before $[Cp_2Ti(III)Cl]$. This results from the binding mode revealed in the DFT calculations. For the association of **L3** to $[Cp_2Ti(III)Cl_2]^-$ K_1 amounts to 0.4 mM^{-1} and for the cleaving of Cl^-***L3** off $Cp_2TiCl_2^-$***L3** K_2 equals 3.0 mM. The formation of the $[Cp_2Ti(III)Cl]$***L3** adduct has an equilibrium constant K_3 of 0.7 mM^{-1}.

A plot (see Figure S14) of the half-wave potentials $E_{1/2}$ of the disk voltammograms as a function of the decadic logarithm of c(**L3**) yields a slope of 60 mV dec^{-1}.

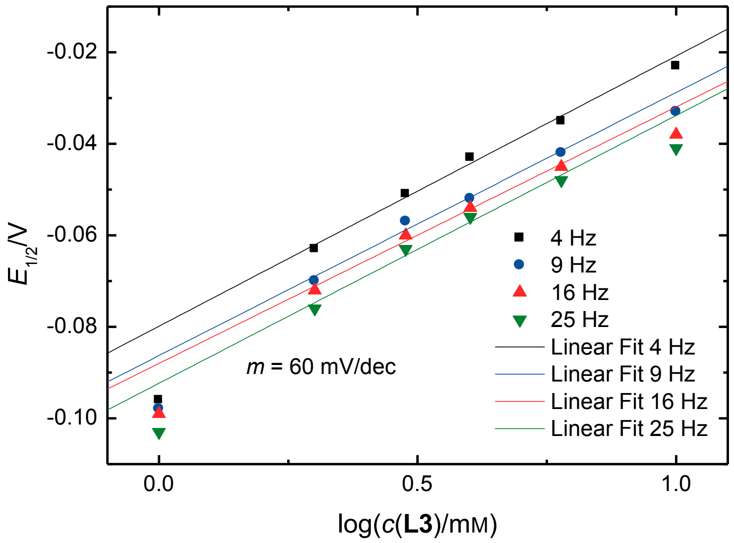

Figure S14. Plot of the half-wave potential $E_{1/2}$ of the RRDE measurements with Cp_2TiCl_2 and **L3**.

This implies a 1:1 complex formation for the Cp_2TiCl_2/**L3** couple as for **L1** before. We propose the electrochemical mesh-scheme shown in Scheme S2 for the Cp_2TiCl_2/**L3** couple.

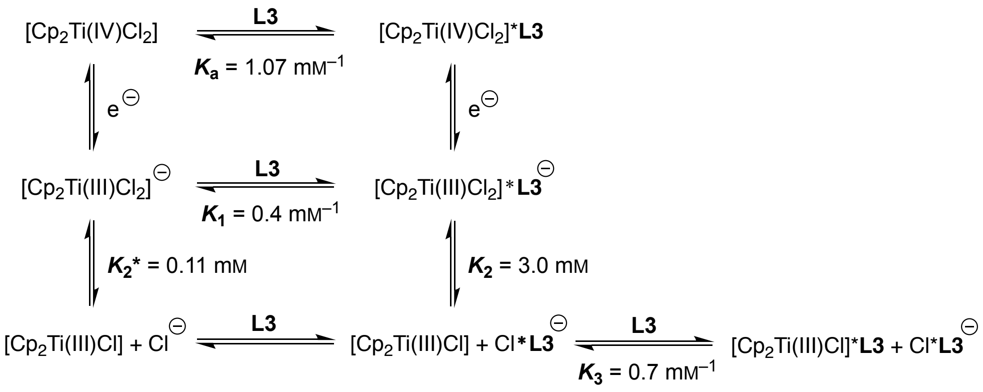

Scheme S2. Electrochemical mesh-scheme for the redox system of Cp_2TiCl_2 in the presence of **L3**.

The mesh-scheme with **L3** looks very similar to the one for **L1**. Only the occurrence of $[Cp_2Ti(III)Cl]$***L3** makes an addition to the scheme necessary. According to the equilibrium constants K_2 and K_2* the C_r

reaction is amplified by a factor of 27 in the presence of **L3**. This is comparable to **L1**. However, the access to $[Cp_2Ti(III)Cl]$*$\textbf{L3}$ increases the extent of the C_r reaction further.

6.4 Squaramide L2

Squaramide **L2** similar to **L3** exhibits a complicated oxidation wave in the range of the neutral Ti(III) with at least two oxidation peaks. Therefore we considered the same equilibrium reactions as we did for **L3**:

$$[Cp_2Ti(IV)Cl_2] + \mathbf{L2} \underset{}{\overset{K_a}{\rightleftharpoons}} [Cp_2Ti(IV)Cl_2]^*\mathbf{L2} \quad (16)$$

$$[Cp_2Ti(III)Cl_2]^{\ominus} + \mathbf{L2} \overset{K_1,\ K_1'}{\rightleftharpoons} [Cp_2Ti(III)Cl_2]^*\mathbf{L2}^{\ominus} \quad (17)$$

$$[Cp_2Ti(III)Cl_2]^*\mathbf{L2}^{\ominus} \overset{K_2}{\rightleftharpoons} [Cp_2Ti(III)Cl] + Cl^*\mathbf{L2}^{\ominus} \quad (18)$$

$$[Cp_2Ti(III)Cl] + \mathbf{L2} \overset{K_3}{\rightleftharpoons} [Cp_2Ti(III)Cl]^*\mathbf{L2} \quad (19)$$

The voltammograms of the 4 and 25 Hz measurements and the corresponding transfer ratios at 0.05, 0.23, 0.42 and 0.72 V ring potential are depicted in Figures S15 and S16.

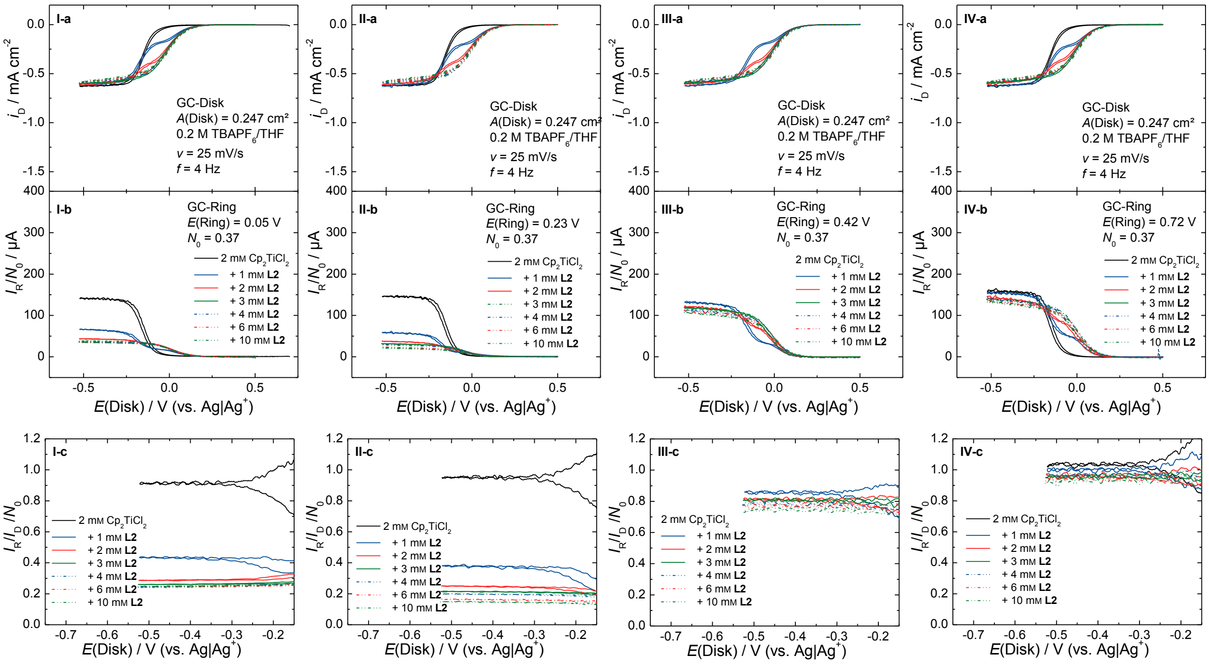

Figure S15: Disk current densities i_D (a), normalized ring currents I_R/N_0 (b) and transfer ratios $I_R/I_D/N_0$ (c) of the RRDE measurements of a solution of 2 mM Cp_2TiCl_2 in 0.2 M $TBAPF_6$/THF with different concentrations of **L2** at a ring potential of 0.05 (I), 0.23 (II), 0.42 (III) and 0.72 V (IV) and a rotation frequency *f* of 4 Hz.

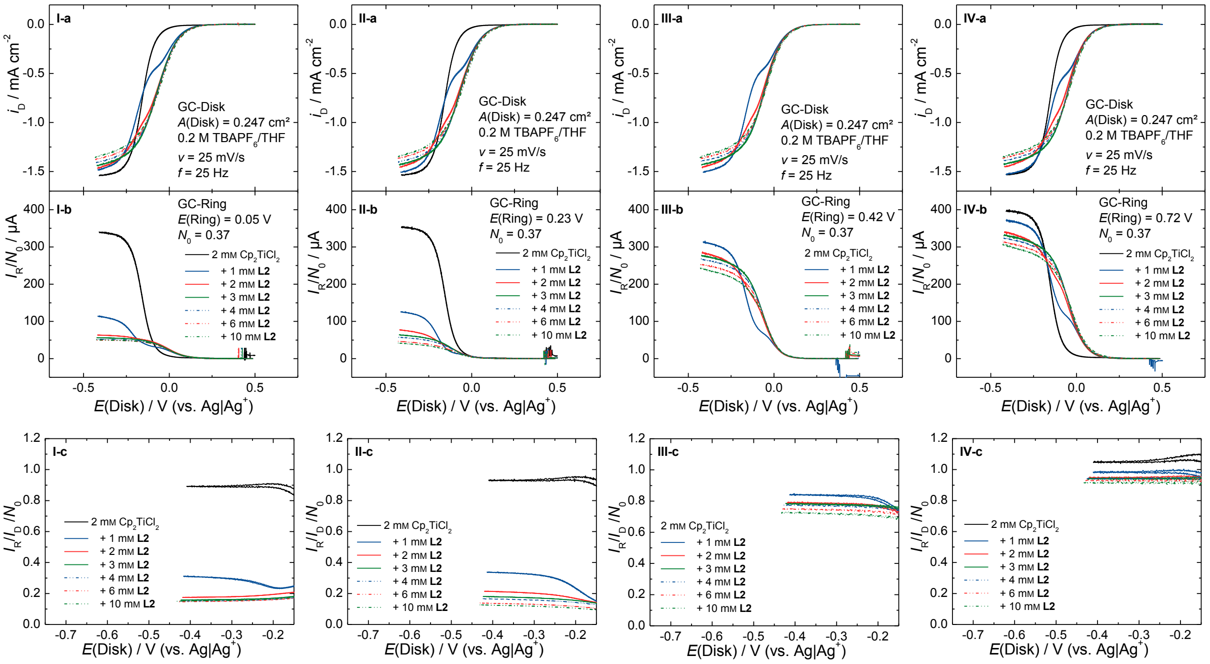

Figure S16: Disk current densities i_D (a), normalized ring currents I_R/N_0 (b) and transfer ratios $I_R/I_D/N_0$ (c) of the RRDE measurements of a solution of 2 mM Cp_2TiCl_2 in 0.2 M $TBAPF_6$/THF with different concentrations of **L2** at a ring potential of 0.05 (I), 0.23 (II), 0.42 (III) and 0.72 V (IV) and a rotation frequency *f* of 25 Hz.

The diffusion limited current densities i_D of the base electrolyte containing Cp_2TiCl_2 and after addition of 10 mM of **L2** and the shoulder currents of the measurements with 1 mM of **L2** are plotted against $f^{1/2}$ in Figure S17.

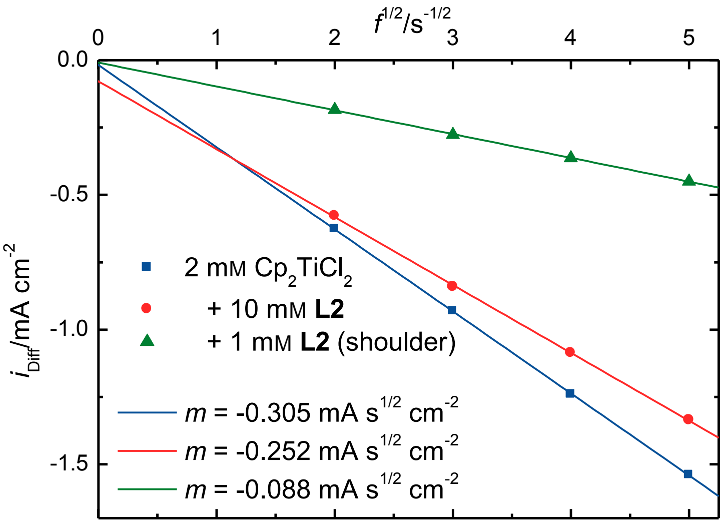

Figure S17. Diffusion limited current densities at the disk i_D as a function of $f^{1/2}$ for Cp_2TiCl_2 and with 10 mM of **L2** or 1 mM of **L2** (shoulder current). The slopes of the linear fits are given as *m*.

Using (7) the ratio of the diffusion coefficients of $[Cp_2Ti(IV)Cl_2]$ and its adduct with **L2** can be calculated to 1.33. Thus, 6.6×10^{-6} cm^2 s^{-1} results for $D([Cp_2Ti(IV)Cl_2]$*$\mathbf{L2})$. Accordingly, the equilibrium constant for (16), K_a, amounts to 1.81 mM^{-1}.

By setting the ring potential for the measurements to 0.05, 0.23, 0.42 and 0.72 V, all Ti(III) species could be detected. The transfer ratios as a function of the concentration of **L2** are given in Figure S18.

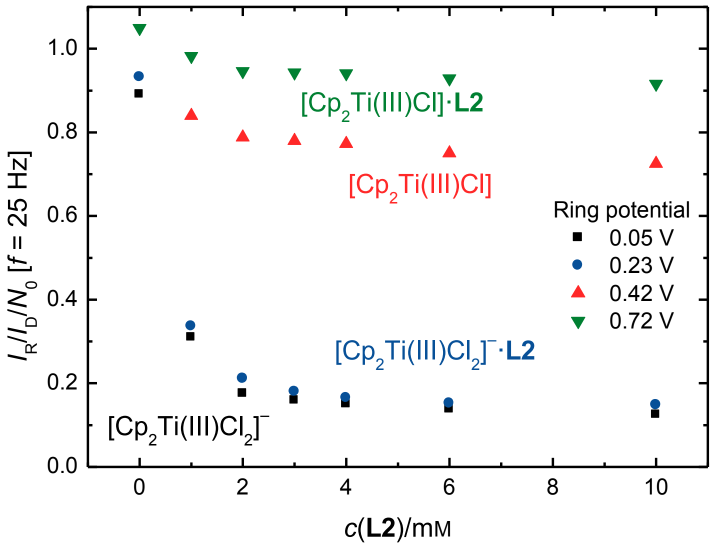

Figure S18. Transfer ratios $I_R/I_D/N_0$ for the measurements of Cp_2TiCl_2 and **L2** at different concentrations of **L2** at ring potentials of 0.05, 0.23, 0.42 and 0.72 V and at a rotation frequency f of 25 Hz.

The transfer ratios yield the equilibrium constants for (17)–(19). For the association of **L2** to $[Cp_2Ti(III)Cl_2]^-$ K_1 amounts to 0.3 mM^{-1} and for the cleaving of Cl^-*$\mathbf{L2}$ off $[Cp_2Ti(III)Cl_2]^-$*$\mathbf{L2}$ K_2 equals 54 mM. The formation of the $[Cp_2Ti(III)Cl]$*$\mathbf{L2}$ adduct has an equilibrium constant K_3 of 0.5 mM^{-1}.

A plot (see Figure S19) of the half-wave potentials $E_{1/2}$ of the disk voltammograms yields a slope of 30 mV dec^{-1} for **L2**.

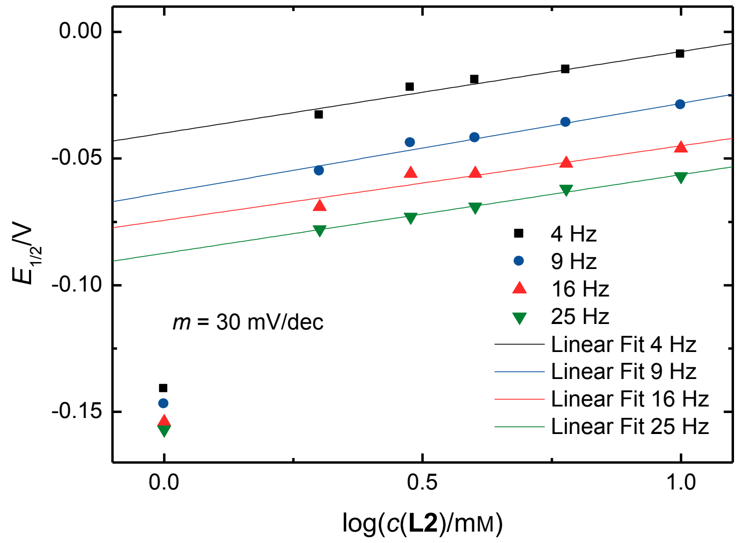

Figure S16. Plot of the half-wave potential $E_{1/2}$ of the RRDE measurements with Cp_2TiCl_2 and **L2**.

The smaller slope in the plot must originate from a different Nernst equation. Here the redox potential is proportional to log(c(**L2**)^q) wih $q = p/2$ (compare (11)). This can be explained with a 2:1 complexation of [Cp_2Ti(III)Cl] with **L2** as shown in reaction (20):

$$2\,[\mathrm{Cp_2Ti(III)Cl}] + \mathbf{L2} \underset{}{\overset{K_3'}{\rightleftharpoons}} ([\mathrm{Cp_2Ti(III)Cl}])_2{}^{*}\mathbf{L2} \qquad (20)$$

Here, two molecules of [Cp_2Ti(III)Cl] are bound to a single molecule of **L2**, resulting in a 2:1 complexation. This result was validated by the DFT calculations. Evaluation of the transfer ratios considering (20) instead of (19), different values for K_1' (0.2 mM^{-1}) and K_3' (0.1 mM^{-2}) are obtained. K_2 remains unchanged. In reality, the equilibria from (19) and (20) are likely to be present side by side. The given numbers for $K_1^{(\prime)}$ and $K_3^{(\prime)}$ can therefore only be considered as a simplification. We propose the electrochemical mesh-scheme shown in Scheme S3 for the Cp_2TiCl_2/**L2** couple.

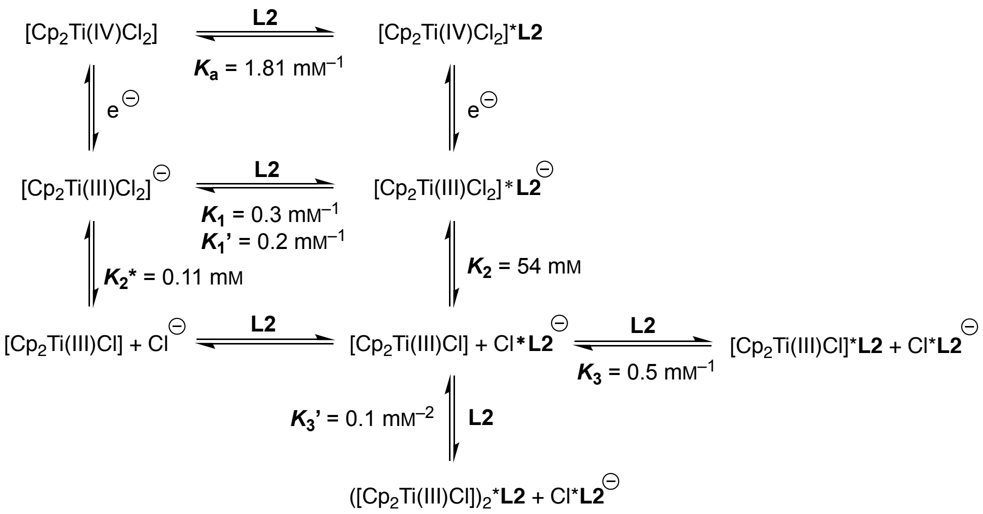

Scheme S3. Electrochemical mesh-scheme for the redox system of Cp_2TiCl_2 in the presence of **L2**.

The mesh-scheme with **L2** looks very similar to the one for **L3**. However it adds the formation of the 2:1 adduct. According to the equilibrium constants the C_r reaction is amplified by a factor of 491 in the presence of **L2**. The access to [Cp_2Ti(III)Cl]***L2** and ([Cp_2Ti(III)Cl])$_2$***L2** increases the extent of the C_r reaction even more.

6.5 Comparison

In order to easily compare the solutions of Cp_2TiCl_2 with **L1** – **L3** it is helpful to calculate the solution composition of the discussed titanocene species in %. Table S1 shows the compositions before and after reduction for a 1:1 mixture of 2 mM Cp_2TiCl_2 and each additive. For **L2**, (20) is not considered here.

Table S1. Solution compositions of titanocene species before (left) and after reduction (right) for 2 mM Cp_2TiCl_2 and with the additives **L1** – **L3** in a 1:1 mixture in %.

	[Cp_2Ti(IV)Cl_2]	[Cp_2Ti(IV)Cl_2]***L**	[Cp_2Ti(III)Cl_2]$^-$	[Cp_2Ti(III)Cl_2]$^-$***L**	[Cp_2Ti(III)Cl]	[Cp_2Ti(III)Cl]***L**
no **L**	100	—	79	—	21	—
**L1**	56	44	14	28	58	0
**L2**	40	60	19	4	60	17
**L3**	49	51	32	12	25	31

From these values a trend in the receptor ability of the additives can be derived. The binding to [Cp_2Ti(IV)Cl_2] decreases in the order **Z** > **X** > **Y**. However, [Cp_2Ti(IV)Cl_2] and [Cp_2Ti(IV)Cl_2]***L** are present in the solution in similar amounts in all three cases. All additives can effectively reduce the amount of anionic titanocene in the reduced solutions and increase the amount of active catalyst. The total amount of neutral Ti(III) complexes is however largest for **L2**, followed by **L1** and **L3**.

7. Density Functional Theory Calculations

7.1 General Information

All visualizations of structures were created with UCSF Chimera[S13] 1.14.0.

We used the xTB,[S14] CREST[S15] and ENSO[S16] programs to determine the structures with the lowest free energy in THF solution for each ligand **L** and all complexes. These calculations were conducted in the same workflow. Manually prepared starting structures, which are initially pre-optimized with the GFN2-xTB[GBSA] tight binding model, are used in the CREST program that employs MTD at the same level in order to obtain a relative complete ensemble of likely structures. The ENSO program determines the equilibrium (Boltzmann) populations for a few low-lying conformers at higher theoretical levels in three steps using TURBOMOLE.[S17] First, already relatively accurate B97-3c[DCOSMO-RS(THF)] (a composite low-cost DFT method[S18]) single point energies are calculated on the CREST ensemble. Structures within an energy threshold of 4 kcal mol^{-1} above the lowest lying structure are then fully optimized at the same level. In this first filtering step thermostatistical free energies in the modified rigid-rotor/harmonic-oscillator (mRRHO) approximation[S19] calculated with GFN2-xTB[GBSA] and the free energy of solvation in THF calculated with the accurate COSMO-RS[S20] solvation model are added. Finally, for all structures within a 2 kcal mol^{-1} threshold an even better single point energy is computed at the PW6B95-D3/def2-TZVPP[S21] hybrid DFT level which basically replaces the corresponding B97-3c energy. In summary, the final complete total free energy used consists of the mRRHO part from the GFN2-xTB treatment, the COSMO-RS part in THF for solvation and the basic electronic energy with the PW6B95-D3 functional. In the following, the conformer of each species with the lowest total free energy is given, if not stated otherwise.

7.2 Calculated Structures

The basic receptor ability of the discussed additives can already give useful information for later analysis. The calculated structures of the H-bond donors **L1**, **L2** and **L3** are given in Figure S17. These conformers are the minimum structures in the electric field of a capacitor resembling the ε_r of THF.

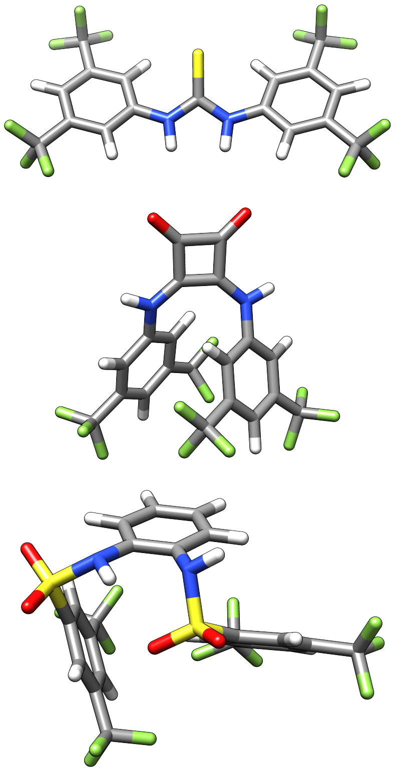

L1:

$\Delta G_{298.15}$ = –0.8 kcal mol^{-1}

L2:

E_{el} = –4.9 kcal mol^{-1}

L3:

$\Delta G_{298.15}$ = –0.1 kcal mol^{-1}

Figure S17. Minimum structures of the H-bond donors **L1**, **L2** and **L3** obtained from DFT calculations. The energetic differences ($\Delta G_{298.15}$) to the next higher lying conformers are given in kcal mol^{-1}. The number shown for **L2** is the energy difference to the respective conformer in the first optimization step (E_{el}) as no conformer was within the threshold.

L1 shows a preferred orientation that allows H-bonding to a single H-bond acceptor with both N-H groups. In contrast, the N-H groups in **L2** and **L3** point into different directions. In order to properly discuss the reaction energies of the adduct formations, the binding affinity of the anion receptors to solvent molecules has to be assessed. The corresponding structure optimizations can be considered as a first approximation to explicit solvation. The direct anion receptor ability of each additive can be estimated by complexation with a simple anionic species. The Cl^- anion is a natural choice in the context of our investigations. Therefore, expanding on the details given in the manuscript the supramolecular complexes of the receptors with THF and Cl^- are depicted in Figure S18.

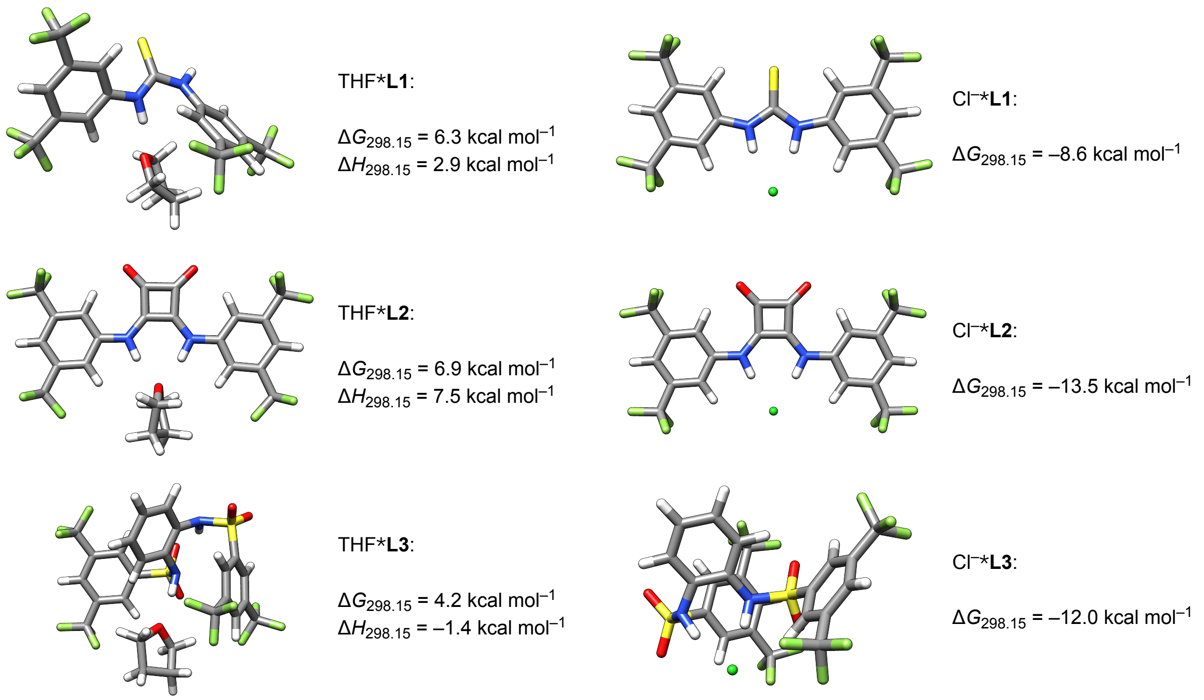

Figure S18. Computed structures of the THF***L** (left) and Cl^-***L** (right) adducts of **L1**, **L2** and **L3**. $\Delta G_{298.15}$ and $\Delta H_{298.15}$ are the Gibbs energy and reaction enthalpy of association for each adduct.

The calculations reveal **L2** as the best H-bond donor. It binds to the oxygen atom of THF with both N—H groups. **L1** and **L3** only form a single H-bond. This observation emphasizes the weak dipole in the THF molecule. In the Cl^-***L** complexes a dramatic change is evident. All receptors bind with both N—H groups. This reflects the association free enthalpies, which are much more strongly binding for Cl^- than for THF.

The calculations of the $[Cp_2Ti(IV)Cl_2]$***L2** adduct revealed a van der Waals complex as lowest energy structure. It does not align with the experimental observations but is depicted in Figure S19 for reference.

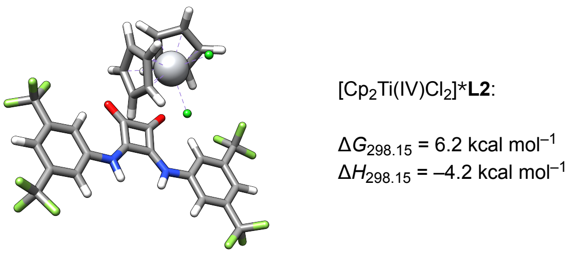

Figure S19. Van der Waals complex found by calculation for the $[Cp_2Ti(IV)Cl_2]$***L2** adduct. $\Delta G_{298.15}$ and $\Delta H_{298.15}$ are the Gibbs energy and reaction enthalpy of association for this adduct.

For the adduct formation between $[Cp_2Ti(III)Cl]$ and **L** calculations without and with a molecule of THF binding to the free coordination site of Ti come to distinctly different results. The important

aspects have been highlighted in the manuscript. In Figure S20 we show the full set of calculated structures.

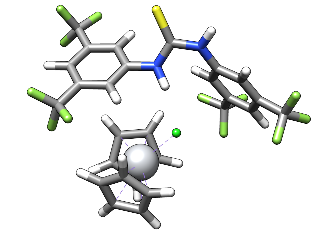

[Cp_2Ti(III)Cl]***L1**:

$\Delta G_{298.15}$ = 10.0 kcal mol^{-1}
$\Delta H_{298.15}$ = 3.7 kcal mol^{-1}

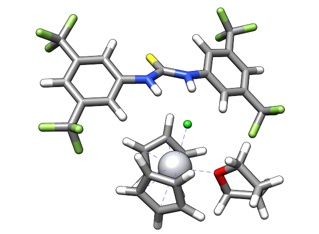

[Cp_2Ti(III)Cl](THF)***L1**:

$\Delta G_{298.15}$ = 5.2 kcal mol^{-1}
$\Delta H_{298.15}$ = –6.9 kcal mol^{-1}

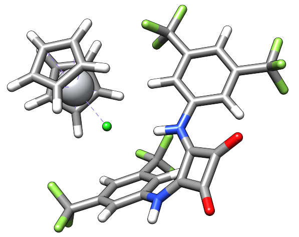

[Cp_2Ti(III)Cl]***L2**:

$\Delta G_{298.15}$ = 8.8 kcal mol^{-1}
$\Delta H_{298.15}$ = 3.7 kcal mol^{-1}

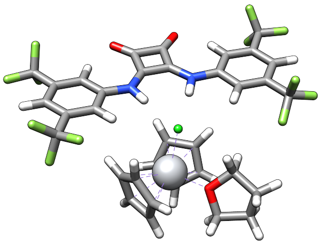

[Cp_2Ti(III)Cl](THF)***L2**:

$\Delta G_{298.15}$ = 1.8 kcal mol^{-1}
$\Delta H_{298.15}$ = –8.0 kcal mol^{-1}

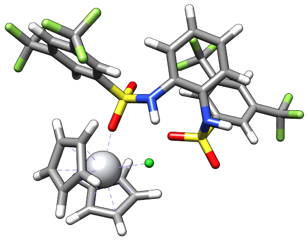

[Cp_2Ti(III)Cl]***L3**:

$\Delta G_{298.15}$ = 2.8 kcal mol^{-1}
$\Delta H_{298.15}$ = –9.1 kcal mol^{-1}

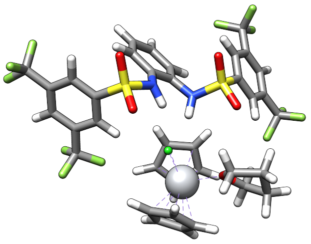

[Cp_2Ti(III)Cl](THF)***L3**:

$\Delta G_{298.15}$ = 4.4 kcal mol^{-1}
$\Delta H_{298.15}$ = –11.3 kcal mol^{-1}

Figure S20. Calculated structures of the [Cp_2Ti(III)Cl]***L** (left) and [Cp_2Ti(III)Cl](THF)***L** complexes (right). $\Delta G_{298.15}$ and $\Delta H_{298.15}$ are the Gibbs energy and reaction enthalpy of association for each adduct starting from [Cp_2Ti(III)Cl], **L** (and THF).

Calculated structures of the 2:1 adducts between [Cp_2Ti(III)Cl] and **L2** without THF and with a single THF molecule are depicted in Figure S21. In both structures coordination of a carbonyl oxygen of **L2** to the Ti center can be observed. A calculation of ([Cp_2Ti(III)Cl](THF))$_2$***L2** resultet in a van der Waals complex, which would have to be considered as a 1:1 complex in the context of our investigations.

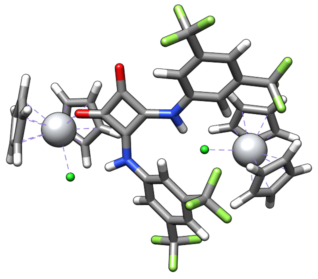

([Cp_2Ti(III)Cl])$_2$*__L2__:

$\Delta G_{298.15}$ = 8.2 kcal mol^{-1}
$\Delta H_{298.15}$ = –2.8 kcal mol^{-1}

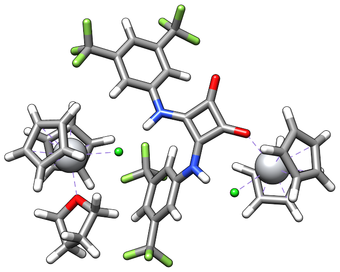

([Cp_2Ti(III)Cl])$_2$(THF)*__L2__:

$\Delta G_{298.15}$ = 3.7 kcal mol^{-1}
$\Delta H_{298.15}$ = –18.4 kcal mol^{-1}

Figure S21. Calculated structures of the ([Cp_2Ti(III)Cl])$_2$*__L2__ and ([Cp_2Ti(III)Cl])$_2$(THF)*__L2__ complexes. $\Delta G_{298.15}$ and $\Delta H_{298.15}$ are the Gibbs energy and reaction enthalpy of association for each adduct starting from [Cp_2Ti(III)Cl], **L** (and THF).

8. References

[S1] N. Vallavoju, S. Selvakumar, B. C. Pemberton, S. Jockusch, M. P. Sibi, J. Sivaguru, *Angew. Chem. Int. Ed.* **2016**, *55*, 5446–5451; *Angew. Chem.* **2016**, *128*, 5536–5541.

[S2] A. Rostami, A. Colin, X. Y. Li, M. G. Chudzinski, A. J. Lough, M. S. Taylor, *J. Org. Chem.* **2010**, *75*, 3983–3992.

[S3] V. Amendola, L. Fabbrizzi, L. Mosca, F.-P. Schmidtchen, *Chem. Eur. J.* **2011**, *17*, 5972–5981.

[S4] J. Thelemann, B. Illarionov, K. Barylyuk, J. Geist, J. Kirchmair, P. Schneider, L. Anthore, K. Root, N. Trapp, A. Bacher, et al., *ChemMedChem* **2015**, *10*, 2090–2098.

[S5] A. Gansäuer, M. Behlendorf, D. Von Laufenberg, A. Fleckhaus, C. Kube, D. V. Sadasivam, R. A. Flowers II, *Angew. Chem. Int. Ed.* **2012**, *51*, 4739–4742; *Angew. Chem.* **2012**, *124*, 4819-4823.

[S6] a) A. J. Bard, L. R. Faulkner, *Electrochemical Methods. Fundamentals and Applications, 2nd ed.*; Wiley, New York, **2001**; b) A. Jutand, *Chem. Rev.* **2008**, *108*, 2300–2347; c) W. E. Geiger, *Coord. Chem. Rev.* **2013**, *257*, 1459–1471.

[S7] a) C. Sandford, M. A. Edwards, K. J. Klunder, D. P. Hickey, M. Li, K. Barman, M. S. Sigman, H. S. White, S. D. Minteer, *Chem. Sci.* **2019**, *10*, 6404–6422; b) W. J. Albery, *Trans. Faraday Soc.*

1966, *62*, 1915–1919; c) W. J. Albery, S. Bruckenstein *Trans. Faraday Soc.* **1966**, *62*, 1920–1931; d) K. B. Prater, A. J. Bard, *J. Electrochem. Soc.* **1970**, *117*, 1517–1520; e) A. J. Bard, L. R. Faulkner, *Electrochemical Methods, 2nd ed.*; Wiley, New York, **2001**; Chapter 9; f) W. J. Albery, M. L. Hitchman, *Ring-Disc Electrodes*, Oxford: Clarendon Press, **1971**.

[S8] a) R. J. Enemærke, J. Larsen, T. Skrydstrup, K. Daasbjerg, *Organometallics* **2004**, *23*, 1866–1874; b) R. J. Enemærke, J. Larsen, T. Skrydstrup, K. Daasbjerg, *J. Am. Chem. Soc.* **2004**, *126*, 7853–7864; c) R. J. Enemærke, J. Larsen, G. H. Hjøllund, T. Skrydstrup, K. Daasbjerg, *Organometallics* **2005**, *24*, 1252–1262.

[S9] a) Y. Mugnier, C. Moise, E. Laviron, *J. Organomet. Chem.* **1981**, *204*, 61–66; b) E. Samuel, J. Vedel, *Organometallics* **1989**, *8*, 237–241.

[S10] As cited in: A. J. Bard, L. R. Faulkner. *Electrochemical Methods: Fundamentals and Applications, 2nd ed.*; John Wiley & Sons Inc.: New York, Weinheim, **2001**, p 339.

[S11] C. Carvajal, K. J. Tölle, J. Smid, M. Szwarc, *J. Am. Chem. Soc.* **1965**, *87*, 5548–5553.

[S12] J.-M. Saveant, *J. Phys. Chem. B* **2001**, *105*, 8995–9001.

[S13] E. F. Pettersen, T. D. Goddard, C. C. Huang, G. S. Couch, D. M. Greenblatt, E. C. Meng, T. E. Ferrin, *J. Comput. Chem.* **2004**, *25*, 1605–1612.

[S14] C. Bannwarth, E. Caldeweyher, S. Ehlert, A. Hansen, P. Pracht, J. Seibert, S. Spicher, S. Grimme, *Wiley Interdiscip. Rev. Comput. Mol. Sci.* **2020**, DOI: 10.1002/wcms.1493.

[S15] a) P. Pracht, F. Bohle, S. Grimme, *Phys. Chem. Chem. Phys.* **2020**, *22*, 7169–7192; b) S. Grimme, *J. Chem. Theory Comput.* **2019**, *15*, 2847–2862.

[S16] S. Grimme, C. Bannwarth, S. Dohm, A. Hansen, J. Pisarek, P. Pracht, J. Seibert, F. Neese, *Angew. Chem. Int. Ed.* **2017**, *56*, 14763–14769.

[S17] a) R. Ahlrichs, M. Baer, M. Haeser, H. Horn, C. Koelmel, *Chem. Phys. Lett.* **1989**, *162*, 165–169; b) O. Treutler, R. Ahlrichs, *J. Chem. Phys.* **1995**, *102*, 346–354.

[S18] J. G. Brandenburg, C. Bannwarth, A. Hansen, S. Grimme, *J. Chem. Phys.* **2018**, *148*, 064104.

[S19] S. Grimme, *Chem. Eur. J.* **2012**, *18*, 9955–9964.

[S20] C. C. Pye, T. Ziegler, E. van Lenthe, J. N. Louwen, *Can. J. Chem.* **2009**, *87*, 790.

[S21] a) Y. Zhao, D. G. Truhlar, *J. Phys. Chem. A* **2005**, *109*, 5656–5667; b) S. Grimme, J. Antony, S. Ehrlich, H. Krieg, *J. Chem. Phys.* **2010**, *132*, 154104; c) S. Grimme, S. Ehrlich, L. Goerigk, *J. Comput. Chem.* **2011**, *32*, 1456–1465; d) F. Weigend, R. Ahlrichs, *Phys. Chem. Chem. Phys.* **2005**, *7*, 3297–3305.

9. Structural Data (xyz-Format)

L1 (Fig. S17, entry 1):

40

S	0.0049776	0.9609810	1.1164922
C	0.0011123	−0.3416911	0.0581255
N	1.1283206	−0.9608054	−0.3995179
C	2.4638247	−0.5788993	−0.2376718
C	2.8709839	0.7527421	−0.2590131
C	4.2202212	1.0565337	−0.1440093
C	5.1783814	0.0613812	−0.0167888
C	4.7601079	−1.2632704	−0.0258371
C	3.4178437	−1.5917486	−0.1370008
H	3.0986479	−2.6223798	−0.1362638
C	5.8027395	−2.3393309	0.0550467
F	5.2823918	−3.5642795	0.2992485
F	6.5102733	−2.4417510	−1.1057146
F	6.7115416	−2.0953108	1.0342690
H	6.2235500	0.3089187	0.0742372
C	4.6459070	2.4959231	−0.1103531
F	4.6600743	2.9836379	1.1622576
F	5.8923919	2.6812569	−0.6072617
F	3.8160339	3.2989927	−0.8202843
H	2.1434965	1.5360708	−0.3761643
H	1.0080519	−1.8628451	−0.8379202
N	−1.1294014	−0.9522608	−0.4018930
C	−2.4644273	−0.5708758	−0.2366287
C	−3.4179960	−1.5858351	−0.1499800
C	−4.7606224	−1.2607126	−0.0375840
C	−5.1806965	0.0634295	−0.0134243
C	−4.2230507	1.0606700	−0.1261942
C	−2.8727829	0.7603118	−0.2416428
H	−2.1455553	1.5457539	−0.3457451
C	−4.6485903	2.4997211	−0.0772052
F	−3.8315980	3.3061933	−0.7984953
F	−5.9036845	2.6877961	−0.5501120
F	−4.6386979	2.9812781	1.1977796
H	−6.2266377	0.3068689	0.0777037
C	−5.8018894	−2.3391195	0.0278293
F	−6.5064427	−2.4288122	−1.1356596
F	−5.2794415	−3.5662889	0.2580480
F	−6.7130268	−2.1092212	1.0080375
H	−3.0971426	−2.6160695	−0.1597688
H	−1.0112183	−1.8527319	−0.8418510

L2 (Fig. S17, entry 2):

44

O	−5.7173392	−1.6510425	−0.0254025
C	−4.8973227	−0.7536750	−0.0313184
C	−3.4205576	−0.6933968	−0.0729465
N	−2.5486540	−1.6992215	−0.2044017
C	−1.2455006	−1.6410191	−0.7058255
C	−0.8146707	−0.5706297	−1.4879820
C	0.4938997	−0.5403300	−1.9371858
C	1.3792859	−1.5746026	−1.6553201
C	0.9222818	−2.6498404	−0.9067434
C	−0.3794759	−2.6954829	−0.4317259
H	−0.7174429	−3.5163858	0.1803655
C	1.8351869	−3.8122351	−0.6389447
F	3.1417886	−3.4598180	−0.6431815
F	1.5890899	−4.4031428	0.5542664
F	1.6917406	−4.7828036	−1.5868714
H	2.3965027	−1.5359336	−2.0104487
C	0.9882406	0.6090901	−2.7676882
F	0.0609280	1.5802395	−2.9335927
F	1.3604367	0.2066357	−4.0128263
F	2.0805158	1.1973999	−2.2142782
H	−1.4939907	0.2264583	−1.7362326
H	−2.9251556	−2.6250554	−0.0500724
C	−3.4170470	0.7058454	0.0752762
N	−2.5404040	1.7075859	0.2057950
C	−1.2369057	1.6444005	0.7062047
C	−0.3683668	2.6975431	0.4340090
C	0.9338268	2.6474595	0.9073697
C	1.3887086	1.5687883	1.6521232
C	0.5009614	0.5365285	1.9328926
C	−0.8082626	0.5712005	1.4855716
H	−1.4886490	−0.2248909	1.7335887
C	0.9932039	−0.6151352	2.7614552
F	0.0660569	−1.5865751	2.9230146
F	2.0872154	−1.2016375	2.2093330
F	1.3626326	−0.2154245	4.0086603
H	2.4062933	1.5285807	2.0041938
C	1.8515233	3.8071744	0.6439078
F	1.5921356	4.4179281	−0.5368400
F	3.1553372	3.4459512	0.6231755
F	1.7290360	4.7645438	1.6081275
H	−0.7057359	3.5217045	−0.1739804
H	−2.9139702	2.6349728	0.0536237
C	−4.8937286	0.7730245	0.0336664
O	−5.7096598	1.6739813	0.0271205

L3 (Fig. S17, entry 3):

54

C	1.3831636	-2.2432192	-1.6855691
C	1.9716277	-1.5194274	-2.7224817
C	1.1886149	-0.9003629	-3.6831844
C	-0.1939314	-1.0197370	-3.6540766
C	-0.7879442	-1.7616754	-2.6466346
C	-0.0171428	-2.3693682	-1.6610210
N	-0.6383356	-3.1849354	-0.6647506
S	-1.1160414	-2.4438537	0.7944608
O	-1.9078792	-3.4497452	1.5162619
O	0.0994577	-1.8585186	1.3837890
C	-2.1850893	-1.0745459	0.3304036
C	-1.5997407	0.1240111	-0.0457683
C	-2.4220187	1.1615050	-0.4598662
C	-3.8026459	1.0040540	-0.4903622
C	-4.3592157	-0.2074390	-0.1036283
C	-3.5550421	-1.2645036	0.3053799
H	-3.9815222	-2.2080581	0.6068116
C	-5.8531403	-0.3798203	-0.0635114
F	-6.3266052	-0.2745810	1.2073484
F	-6.2352277	-1.5973886	-0.5201461
F	-6.5044543	0.5443862	-0.8028444
H	-4.4325504	1.8194618	-0.8056388
C	-1.8080691	2.4480423	-0.9384750
F	-2.6137169	3.5140321	-0.7325323
F	-1.5487443	2.4026625	-2.2728033
F	-0.6325476	2.7106382	-0.3209033
H	-0.5286917	0.2425233	-0.0006188
H	-1.4398237	-3.6933490	-1.0221634
H	-1.8616617	-1.8737663	-2.6009327
H	-0.8047697	-0.5414202	-4.4064540
H	1.6707752	-0.3328852	-4.4667654
H	3.0455602	-1.4591171	-2.7843615
N	2.1449740	-2.9119892	-0.7118380
S	3.5563386	-2.2539898	-0.0259928
O	3.7875100	-3.0693887	1.1752951
O	4.5701259	-2.1570605	-1.0856243
C	3.1370550	-0.5764130	0.4747344
C	2.5690303	-0.3779883	1.7247193
C	2.1804752	0.9038206	2.0730525
C	2.3563051	1.9699154	1.1939622
C	2.9435023	1.7432001	-0.0390105
C	3.3460215	0.4635407	-0.4111324
H	3.8135732	0.2892727	-1.3655703
C	3.1696680	2.8684689	-1.0097578
F	2.5783097	2.6225284	-2.2070377
F	4.4934024	3.0483010	-1.2634625
F	2.6900366	4.0498588	-0.5661110
H	2.0292038	2.9581025	1.4730288
C	1.5115894	1.1694318	3.3925218
F	1.5560914	0.1090978	4.2262867
F	2.0820080	2.2138928	4.0463474
F	0.1997127	1.4889637	3.2259059

H	2.4207204	-1.2085781	2.3940660
H	1.5859228	-3.3188112	0.0293834

*THF*L1 (Fig. S18, left entry 1):*

53

S	-1.2336927	-3.1945195	1.7473956
C	-0.2811730	-1.9542410	1.1354278
N	1.0778930	-2.0854470	1.1250669
C	2.0358777	-1.4294695	0.3473684
C	1.7472930	-0.8586341	-0.8948526
C	2.7631109	-0.2668348	-1.6261398
C	4.0738811	-0.2372512	-1.1619035
C	4.3506429	-0.8307603	0.0601363
C	3.3487226	-1.4190288	0.8177725
H	3.5689899	-1.8667868	1.7739872
C	5.7404293	-0.7676566	0.6238885
F	5.8825803	0.2899733	1.4751251
F	6.0619143	-1.8764837	1.3347068
F	6.6873690	-0.6234031	-0.3310783
H	4.8522839	0.2255826	-1.7458512
C	2.4793874	0.3332214	-2.9735105
F	2.9293958	1.6129765	-3.0609861
F	1.1604738	0.3617733	-3.2725750
F	3.0936584	-0.3568953	-3.9714667
H	0.7442524	-0.8872809	-1.2848207
H	1.4234125	-2.8338180	1.7070869
N	-0.7382313	-0.7503569	0.7021568
C	-2.0582672	-0.3910473	0.4303667
C	-2.9549402	-1.2571501	-0.1898581
C	-4.2404849	-0.8180416	-0.4726078
C	-4.6528986	0.4699961	-0.1598019
C	-3.7385420	1.3308552	0.4335993
C	-2.4529661	0.9114664	0.7337810
H	-1.7471327	1.5806827	1.2007724
C	-4.1852988	2.7203072	0.7787379
F	-5.1208434	2.7179175	1.7668618
F	-4.7542594	3.3506314	-0.2830478
F	-3.1714612	3.5096082	1.2042761
H	-5.6535950	0.8005815	-0.3861544
C	-5.2215659	-1.7752950	-1.0850737
F	-6.1593246	-1.1497855	-1.8367916
F	-5.8955760	-2.4771400	-0.1310607
F	-4.6238437	-2.6915423	-1.8851675
H	-2.6438726	-2.2520480	-0.4556554
H	-0.0958020	0.0419992	0.8191901
O	0.7537186	1.5631821	1.3098852
C	1.2591827	2.5250359	0.3565442
C	2.6694884	2.8450750	0.8074177
H	3.3686667	2.1095788	0.4114690
H	2.9925696	3.8322178	0.4850008
C	2.5578125	2.7134755	2.3226490
H	2.1204056	3.6152633	2.7510883
H	3.5145214	2.5361856	2.8082180
C	1.6103348	1.5381045	2.4778101

H	0.9802223	1.5975823	3.3633070
H	2.1483478	0.5881568	2.4911714
H	0.6176495	3.4090304	0.3789548
H	1.2056609	2.0784821	-0.6336194

*THF*L2 (Fig. S18, left entry 2):*

57

H	1.0981758	0.6646117	-0.0006273
N	1.5410682	-0.2601477	0.0051092
C	0.7006365	-1.3025641	0.0071256
C	-0.7103992	-1.3009716	0.0158199
N	-1.5484910	-0.2566670	0.0255902
C	-2.9412024	-0.2318887	0.0219294
C	-3.7324648	-1.3740817	0.0092952
C	-5.1159762	-1.2448176	0.0023570
C	-5.7328834	-0.0051505	0.0063421
C	-4.9265810	1.1299483	0.0201522
C	-3.5499666	1.0270957	0.0295710
H	-2.9367803	1.9156816	0.0428547
C	-5.5906482	2.4764709	0.0260707
F	-6.3781785	2.6442606	1.1197771
F	-4.7037238	3.5000319	0.0214583
F	-6.3925533	2.6465087	-1.0564989
H	-6.8066304	0.0858965	-0.0014696
C	-5.9325843	-2.5072858	-0.0120974
F	-7.2629754	-2.2753817	-0.0102206
F	-5.6639693	-3.2632821	-1.1092932
F	-5.6650149	-3.2876614	1.0679215
H	-3.2747910	-2.3534225	0.0052297
H	-1.1040078	0.6672005	0.0321724
C	-0.7658389	-2.7798512	0.0103628
O	-1.6234968	-3.6446809	0.0114089
C	0.7527758	-2.7815466	0.0032067
O	1.6085270	-3.6482543	-0.0033345
C	2.9337933	-0.2384832	0.0033110
C	3.7226520	-1.3824214	-0.0008927
C	5.1064554	-1.2561430	0.0001516
C	5.7260899	-0.0178342	0.0058761
C	4.9222110	1.1190379	0.0089773
C	3.5453838	1.0191100	0.0067349
H	2.9341861	1.9090374	0.0071708
C	5.5891062	2.4641597	0.0152150
F	6.3838413	2.6264995	1.1043532
F	4.7042067	3.4895761	0.0195881
F	6.3844059	2.6364199	-1.0719253
H	6.8000543	0.0708754	0.0081819
C	5.9205611	-2.5203034	-0.0068693
F	5.6451897	-3.2981879	1.0730359
F	5.6568406	-3.2777664	-1.1042321
F	7.2513220	-2.2909961	0.0022852
H	3.2629009	-2.3607879	-0.0050178
O	-0.0010423	2.1135618	-0.0136354
C	-0.0512860	2.9204804	-1.2325247
C	-0.2847531	4.3411985	-0.7633112

H	0.1192849	5.0705298	-1.4608421
H	-1.3502838	4.5349136	-0.6427111
C	0.4130484	4.3600420	0.5928142
C	0.0759315	2.9938398	1.1500489
H	0.8288506	2.5897239	1.8191448
H	-0.8968131	2.9882616	1.6399719
H	0.0650730	5.1606557	1.2407044
H	1.4900232	4.4668367	0.4644343
H	-0.8462131	2.5262736	-1.8576633
H	0.9063968	2.8152443	-1.7400383

*THF*L3 (Fig. S18, left entry 3):*

67

H	-0.9432710	2.4664471	1.8639669
N	-1.2898876	3.0593918	1.1141633
S	-2.9927913	2.9963992	1.0938517
O	-3.4057918	3.1609385	2.4953723
O	-3.4525854	3.9122503	0.0402762
C	-3.3647558	1.3168429	0.5716656
C	-3.4917917	0.3304270	1.5372994
C	-3.6443377	-0.9812867	1.1193595
C	-3.6535631	-1.3019177	-0.2352380
C	-3.5235369	-0.2922057	-1.1752221
C	-3.3875634	1.0350098	-0.7809187
H	-3.2858045	1.8229108	-1.5105533
C	-3.5436915	-0.5962419	-2.6465111
F	-3.5291345	-1.9217934	-2.9100647
F	-4.6513381	-0.0860014	-3.2456565
F	-2.4753038	-0.0535310	-3.2851636
H	-3.7522812	-2.3287892	-0.5457031
C	-3.8546610	-2.0743296	2.1301790
F	-3.2109748	-3.2190119	1.7897133
F	-3.4296428	-1.7322618	3.3642706
F	-5.1737703	-2.3938163	2.2413375
H	-3.4442242	0.5851477	2.5838476
C	-0.6086870	2.8706944	-0.1178262
C	-0.4701024	3.9572622	-0.9744693
C	0.1817745	3.8206954	-2.1899500
C	0.7256115	2.5954824	-2.5537128
C	0.6038592	1.5104829	-1.7016463
C	-0.0759832	1.6277430	-0.4927200
N	-0.2872439	0.4719008	0.3100251
S	0.7365627	0.2084458	1.6394066
O	0.3715387	-1.1101536	2.1719635
O	0.6694037	1.4186236	2.4827655
C	2.3875543	0.1230738	0.9386673
C	3.0569090	1.3060520	0.6559390
C	4.2835468	1.2277884	0.0167244
C	4.8326440	-0.0033730	-0.3308814
C	4.1367153	-1.1654065	-0.0375292
C	2.9011430	-1.1131387	0.5981261
H	2.3465332	-2.0119550	0.8150655
C	4.7053742	-2.5147980	-0.3776595
F	5.8056465	-2.4409771	-1.1548586

F	5.0518458	-3.2041299	0.7407174
F	3.8007491	-3.2832349	-1.0390646
H	5.7887934	-0.0459827	-0.8272947
C	5.0484752	2.4740431	-0.3343914
F	6.2865286	2.4756094	0.2223506
F	5.2271030	2.5838944	-1.6771239
F	4.4279460	3.6005302	0.0750129
H	2.6262837	2.2565878	0.9264421
H	-0.2688546	-0.3955428	-0.2431209
H	1.0282761	0.5535850	-1.9676421
H	1.2503193	2.4851080	-3.4921677
H	0.2788081	4.6755970	-2.8442954
H	-0.8876297	4.9056842	-0.6718465
O	0.1193732	-2.0087676	-1.0508403
C	-0.2605639	-3.2364232	-0.3981862
C	-0.6683419	-4.1680480	-1.5175642
H	-0.6054665	-5.2147094	-1.2286253
H	-1.6904796	-3.9605483	-1.8317830
C	0.3243734	-3.7922873	-2.6136593
H	-0.0310702	-4.0335910	-3.6123462
H	1.2687408	-4.3118568	-2.4582773
C	0.5027835	-2.2914797	-2.4180648
H	-0.1449604	-1.7115482	-3.0749297
H	1.5322796	-1.9686464	-2.5711395
H	-1.0521294	-3.0022051	0.3064570
H	0.5917031	-3.6340488	0.1590646

*Cl-*L1 (Fig. 9, entry 1; Fig. S18, right entry 1):*

41

S	-0.0001610	-2.0618251	-0.0022204
C	-0.0001609	-0.3782175	0.0003833
N	1.1175724	0.4082978	0.0015063
C	2.4753422	0.1251753	0.0009105
C	3.3256694	1.2402611	-0.0014877
C	4.6991491	1.0781246	-0.0023962
C	5.2776980	-0.1863569	-0.0007923
C	4.4306973	-1.2829071	0.0019888
C	3.0491016	-1.1468320	0.0028745
H	2.4093951	-2.0118873	0.0049877
C	4.9845478	-2.6779119	0.0030198
F	6.3370723	-2.7127528	0.0072099
F	4.5714811	-3.3863734	1.0892966
F	4.5783255	-3.3849714	-1.0868916
H	6.3478101	-0.3076244	-0.0018009
C	5.5579028	2.3096149	-0.0042353
F	6.8818149	2.0281550	-0.0112315
F	5.3306302	3.0895441	1.0870675
F	5.3198910	3.0940505	-1.0898484
H	2.8950299	2.2324577	-0.0022498
H	0.9212373	1.4227888	0.0019846
N	-1.1175334	0.4086121	0.0014238
C	-2.4755448	0.1256126	0.0007780
C	-3.0492587	-1.1463338	-0.0004474
C	-4.4309139	-1.2826751	-0.0014299

C	-5.2780200	-0.1862834	-0.0008150
C	-4.6994322	1.0781348	0.0007277
C	-3.3259640	1.2406231	0.0013729
H	-2.8963004	2.2332947	0.0018448
C	-5.5583578	2.3094547	0.0011520
F	-6.8825334	2.0277123	0.0019352
F	-5.3262648	3.0915147	-1.0874582
F	-5.3249878	3.0917393	1.0892783
H	-6.3479981	-0.3084280	-0.0020038
C	-4.9844192	-2.6778213	-0.0030720
F	-6.3367886	-2.7133320	-0.0059358
F	-4.5765918	-3.3857499	1.0857611
F	-4.5720031	-3.3851048	-1.0905449
H	-2.4095747	-2.0113984	-0.0008990
H	-0.9210798	1.4230215	0.0022482
Cl	0.0034436	3.3410684	0.0024602

*Cl-*L2 (Fig. 9, entry 2, Fig. S18, right entry 2):*

45

H	1.1014147	0.9494553	0.0050130
N	1.5457894	0.0127638	0.0039158
C	0.7073819	-1.0280001	0.0029940
C	-0.7074073	-1.0280162	0.0032236
N	-1.5459362	0.0127763	0.0027125
C	-2.9364504	0.0334660	0.0014474
C	-3.5498063	1.2881650	-0.0052709
C	-4.9309847	1.3846389	-0.0076233
C	-5.7325043	0.2499677	-0.0025741
C	-5.1082414	-0.9900371	0.0045055
C	-3.7267501	-1.1137350	0.0065699
H	-3.2639520	-2.0908703	0.0118078
C	-5.9200382	-2.2536764	0.0084505
F	-5.6615940	-3.0222847	-1.0835797
F	-7.2532497	-2.0259838	0.0187818
F	-5.6451223	-3.0262551	1.0933253
H	-6.8063327	0.3310370	-0.0040428
C	-5.5422775	2.7557063	-0.0116294
F	-6.8934938	2.7312233	-0.0388361
F	-5.1397975	3.4843575	-1.0871771
F	-5.1836732	3.4704270	1.0890734
H	-2.9312775	2.1758681	-0.0092446
H	-1.1016179	0.9495303	0.0020117
C	-0.7573469	-2.5062777	0.0026394
O	-1.6132533	-3.3766928	0.0030133
C	0.7574783	-2.5062625	0.0026359
O	1.6133893	-3.3765875	0.0015175
C	2.9362594	0.0335640	0.0026475
C	3.7266574	-1.1135168	-0.0047463
C	5.1081763	-0.9897613	-0.0057240
C	5.7323880	0.2502732	0.0006832
C	4.9307860	1.3848523	0.0084413
C	3.5495754	1.2882825	0.0088828
H	2.9309942	2.1760218	0.0148145
C	5.5426744	2.7556319	0.0099682

F	5.1326033	3.4899792	1.0785142
F	6.8938325	2.7307591	0.0468653
F	5.1921185	3.4646125	-1.0970862
H	6.8062611	0.3305431	0.0008924
C	5.9200065	-2.2534357	-0.0119157
F	7.2531158	-2.0261198	-0.0272874
F	5.6407356	-3.0266190	-1.0952900
F	5.6653056	-3.0213156	1.0815559
H	3.2639264	-2.0906808	-0.0094396
Cl	0.0000139	2.7337886	0.0033980

*Cl-*L3 (Fig. 9, entry 3, Fig. 18, right entry 3):*

55

H	-1.5053231	-2.5131281	1.4250046
N	-1.9853172	-1.9213031	2.1255326
S	-3.5853357	-1.6488181	1.6513204
O	-4.0658049	-2.9316603	1.1128774
O	-4.2765945	-0.9810402	2.7675980
C	-3.4833004	-0.4615293	0.3044061
C	-3.3407342	-0.9293366	-0.9912813
C	-3.1375128	-0.0059734	-2.0047903
C	-3.0604950	1.3527370	-1.7263224
C	-3.2002523	1.7885242	-0.4172311
C	-3.4282528	0.8857673	0.6129393
H	-3.5336505	1.2199869	1.6327113
C	-3.1421082	3.2531418	-0.0945014
F	-4.3948714	3.7708951	0.0781351
F	-2.4682424	3.5012181	1.0522336
F	-2.5568869	3.9840572	-1.0686253
H	-2.8591427	2.0545557	-2.5169819
C	-3.0367776	-0.4800584	-3.4262233
F	-4.2744857	-0.5425198	-4.0069463
F	-2.2948222	0.3412517	-4.2024232
F	-2.5061335	-1.7163848	-3.5269835
H	-3.3521855	-1.9882315	-1.1944121
C	-1.2454275	-0.8116045	2.6215359
C	-1.3797984	-0.4891531	3.9666271
C	-0.7130680	0.5940890	4.5128768
C	0.1208057	1.3519868	3.7034735
C	0.2808407	1.0381970	2.3638392
C	-0.4086337	-0.0329349	1.7941152
N	-0.3418372	-0.3545430	0.4296172
S	0.4796545	0.5044853	-0.7367186
O	0.2173463	1.9428671	-0.5740482
O	0.1735376	-0.1828255	-1.9995114
C	2.2317546	0.2173885	-0.4023795
C	2.6760091	-1.0933703	-0.3373181
C	4.0288287	-1.3213984	-0.1303855
C	4.9181347	-0.2619645	0.0118676
C	4.4381168	1.0386946	-0.0473193
C	3.0866002	1.2926458	-0.2570480
H	2.7065578	2.3019379	-0.2936357
C	5.3784760	2.2044243	0.0665194
F	4.8856131	3.1794180	0.8704451

F	6.5872895	1.8572132	0.5629700
F	5.6043447	2.7820487	-1.1454735
H	5.9668978	-0.4501744	0.1723410
C	4.5088030	-2.7431233	-0.0346920
F	4.0398918	-3.5004639	-1.0559396
F	5.8578805	-2.8424526	-0.0559269
F	4.0889872	-3.3338531	1.1142461
H	1.9762544	-1.9172798	-0.4329538
H	-0.3758596	-1.3640784	0.1725460
H	0.9225826	1.6536671	1.7564410
H	0.6630975	2.1933345	4.1128705
H	-0.8347790	0.8350649	5.5597853
H	-2.0302021	-1.1051477	4.5697740
Cl	-0.2191148	-3.3922728	-0.0378396

*Cp2TiCl2*L1 (Fig. 13, entry 1):*

63

S	2.1127157	2.8310114	-2.4729179
C	0.9894533	2.1396831	-1.4320561
N	1.2648653	1.1161134	-0.5822061
C	2.5479210	0.6366144	-0.2913847
C	3.5514224	1.5056670	0.1361456
C	4.7968403	0.9974160	0.4546324
C	5.0672094	-0.3676678	0.3694500
C	4.0571152	-1.2171182	-0.0440616
C	2.7992079	-0.7229942	-0.3768991
H	2.0142075	-1.3835898	-0.7070892
C	4.2855849	-2.6966439	-0.1228033
F	3.6120959	-3.3616448	0.8587712
F	5.5856534	-3.0407727	-0.0037922
F	3.8453154	-3.2153829	-1.3005852
H	6.0439545	-0.7469258	0.6228443
C	5.9029118	1.9090578	0.8984413
F	5.5000471	3.1884729	1.0663230
F	6.9239902	1.9344477	-0.0021802
F	6.4468706	1.5097248	2.0792827
H	3.3416436	2.5582335	0.2181400
H	0.5070425	0.4698564	-0.3792626
N	-0.3020441	2.5708228	-1.4479139
C	-1.3200316	2.4616654	-0.5047304
C	-1.1032151	2.1740480	0.8448364
C	-2.1730181	2.1756890	1.7254992
C	-3.4626028	2.4741827	1.3071445
C	-3.6648531	2.7671458	-0.0357000
C	-2.6176714	2.7490532	-0.9406149
H	-2.7877786	2.9519304	-1.9864294
C	-5.0623854	3.0419490	-0.5110323
F	-5.0962588	3.6865835	-1.7009652
F	-5.7617835	3.8019933	0.3679932
F	-5.7732148	1.8903320	-0.6734879
H	-4.2826515	2.4787704	2.0066889
C	-1.9644013	1.8304290	3.1724498
F	-0.6624006	1.6772420	3.4999357
F	-2.5934395	0.6702343	3.5044067

F	-2.4678290	2.7863032	3.9982436
H	-0.1133258	1.9617539	1.2080572
H	-0.5464751	3.0688620	-2.2904603
Cl	-0.4408885	-1.3994570	0.8264040
Ti	-1.5947706	-2.3408309	-1.0475762
Cl	-0.8807259	-0.6733750	-2.5571791
C	0.3322714	-3.4289236	-2.0140920
C	0.2040188	-3.9122188	-0.7058553
C	-1.0341645	-4.5821929	-0.6012397
C	-1.6441406	-4.5593337	-1.8783217
C	-0.8175616	-3.8221738	-2.7409740
H	-1.0358232	-3.5655367	-3.7634793
H	-2.5944016	-4.9902918	-2.1310120
H	-1.4398491	-5.0275613	0.2908038
H	0.8946867	-3.7474254	0.0998118
H	1.1349778	-2.8245327	-2.3949269
C	-3.7193908	-3.1735902	-0.4063472
C	-3.3919412	-2.1599245	0.5047380
C	-3.3369531	-0.9367772	-0.2039370
C	-3.6461234	-1.1951777	-1.5467362
C	-3.8483847	-2.5820674	-1.6900762
H	-4.0625752	-3.1008623	-2.6080125
H	-3.6580314	-0.4713922	-2.3404197
H	-3.0563456	0.0133339	0.2079883
H	-3.1659017	-2.2946479	1.5476773
H	-3.8145364	-4.2196535	-0.1805278

*Cp2TiCl2*L2 (Fig. 13, entry 2):*

67

H	0.9846716	0.2699952	1.0525515
N	1.3777490	1.1570117	0.7479862
C	0.5052928	2.0521635	0.2734369
C	-0.9006621	1.9778504	0.2003064
N	-1.7158229	0.9804437	0.5699942
C	-3.1091467	0.9184031	0.5747977
C	-3.9229865	1.9462622	0.1123816
C	-5.3020957	1.7933259	0.1569318
C	-5.8934358	0.6370018	0.6408444
C	-5.0649075	-0.3854648	1.0910328
C	-3.6887856	-0.2522209	1.0708629
H	-3.0555601	-1.0500118	1.4285084
C	-5.6846562	-1.6251752	1.6696620
F	-4.8552963	-2.6970432	1.6149998
F	-6.8244350	-1.9767657	1.0290529
F	-6.0119062	-1.4556474	2.9804179
H	-6.9647012	0.5276864	0.6667642
C	-6.1470069	2.9223199	-0.3641864
F	-6.0027975	3.0742039	-1.7085442
F	-5.7998422	4.1093787	0.1959209
F	-7.4652730	2.7462243	-0.1290020
H	-3.4851225	2.8542167	-0.2774990
H	-1.2615915	0.1295633	0.8976634
C	-0.9972292	3.3272639	-0.3985189
O	-1.8747737	4.0687721	-0.8026153

C	0.5152612	3.4179158	-0.2989747
O	1.3422963	4.2662328	-0.5782568
C	2.7707414	1.1700409	0.7255006
C	3.5198632	2.2063579	0.1838538
C	4.9069138	2.1265981	0.2051521
C	5.5658474	1.0360679	0.7469982
C	4.7995782	0.0041623	1.2824983
C	3.4195730	0.0609083	1.2754365
H	2.8342774	-0.7466992	1.6868025
C	5.5113566	-1.1914752	1.8465340
F	6.4486505	-0.8392683	2.7626005
F	6.1642211	-1.8870692	0.8752560
F	4.6761119	-2.0653345	2.4560027
H	6.6421858	0.9831386	0.7580833
C	5.6792908	3.2665030	-0.3977093
F	5.3592265	4.4525766	0.1824815
F	7.0168264	3.1192420	-0.2796836
F	5.4127616	3.4032225	-1.7242842
H	3.0289209	3.0689097	-0.2432743
Cl	1.0993504	-0.6158754	-1.9867582
Ti	0.3716113	-2.6776569	-1.1427526
Cl	-0.0717898	-1.8122008	1.1059167
C	2.3434026	-3.5714961	-2.0948301
C	2.7537947	-2.9150602	-0.9094071
C	2.1749281	-3.5794065	0.1784650
C	1.3844030	-4.6396314	-0.3205169
C	1.5296429	-4.6580531	-1.7253264
H	1.0603981	-5.3516834	-2.3965367
H	0.7735828	-5.3058022	0.2643818
H	2.2668912	-3.2999657	1.2108799
H	3.3558292	-2.0267532	-0.8602001
H	2.5831497	-3.2696299	-3.1003122
C	-1.2562712	-2.2482807	-2.8517837
C	-1.8657401	-1.9702154	-1.6161196
C	-1.9504285	-3.1775422	-0.8867655
C	-1.3663039	-4.1933218	-1.6558555
C	-0.9211429	-3.6137381	-2.8744611
H	-0.4191221	-4.1279179	-3.6737883
H	-1.2472772	-5.2209713	-1.3645837
H	-2.3426422	-3.2873268	0.1076600
H	-2.1809337	-1.0010721	-1.2758969
H	-1.0272678	-1.5297837	-3.6172212

*Cp2TiCl2*L2 (vdW complex, Fig. S19):*

67

H	-0.7043895	2.6431705	-0.1764888
N	-1.2083385	1.9957523	0.4108054
C	-0.4677528	1.1069833	1.0871276
C	0.9314157	0.9072406	1.0731071
N	1.8778100	1.5557850	0.3899341
C	3.2534660	1.3132327	0.3294516
C	3.8739806	0.2784075	1.0173179
C	5.2486424	0.1138292	0.9045800
C	6.0188474	0.9501155	0.1142789
C	5.3796289	1.9799951	-0.5706175
C	4.0138779	2.1702445	-0.4719901
H	3.5287978	2.9734401	-1.0042712
C	6.2192287	2.8903603	-1.4192970
F	7.1536598	3.5470592	-0.6803032
F	5.4953566	3.8359060	-2.0610804
F	6.9005648	2.2029429	-2.3736530
H	7.0847724	0.8145301	0.0305407
C	5.8789067	-1.0362071	1.6377287
F	7.2287059	-0.9724772	1.6584187
F	5.5505851	-2.2291371	1.0690833
F	5.4658452	-1.1010906	2.9291949
H	3.2949521	-0.3873345	1.6402195
H	1.5612511	2.2724460	-0.2469117
C	0.8291764	-0.2009367	2.0505836
O	1.5801091	-0.9695022	2.6239464
C	-0.6688390	0.0065284	2.0489080
O	-1.6050519	-0.5459056	2.6054477
C	-2.5955524	2.1607341	0.3769141
C	-3.4775549	1.3134954	1.0446030
C	-4.8404464	1.5346001	0.9354279
C	-5.3530546	2.5795073	0.1775286
C	-4.4595355	3.4135946	-0.4803324
C	-3.0909671	3.2176878	-0.3874414
H	-2.4049872	3.8701257	-0.9054998
C	-4.9912054	4.5870055	-1.2517357
F	-5.2330243	5.6506670	-0.4342271
F	-6.1605219	4.3074723	-1.8752927
F	-4.1310297	5.0210666	-2.2038630
H	-6.4171036	2.7326548	0.0993212
C	-5.8098632	0.6077710	1.6128204
F	-6.7315955	1.2844692	2.3452626
F	-6.5070252	-0.1325741	0.7070904
F	-5.2078286	-0.2665922	2.4490091
H	-3.1022426	0.5003489	1.6468428
Cl	-0.1735984	-4.4264176	-3.0459792
Ti	-0.7729249	-3.2809083	-1.0603703
Cl	0.8301289	-1.5486320	-1.3328448
C	-0.4471718	-3.6101181	1.2608836
C	-1.2612787	-4.6718113	0.8035783
C	-0.5070708	-5.4361573	-0.1012494
C	0.7916109	-4.8734690	-0.1736159
C	0.8286385	-3.7642416	0.6804145

H	1.6604380	-3.1012682	0.8241941
H	1.5879107	-5.2047965	-0.8151480
H	-0.8651025	-6.2744158	-0.6736335
H	-2.2861890	-4.8377557	1.0761191
H	-0.7417255	-2.8337563	1.9445851
C	-3.1431163	-3.4098834	-1.0257926
C	-2.7973649	-3.1544900	-2.3634395
C	-2.1755025	-1.8933094	-2.4265997
C	-2.1758302	-1.3489467	-1.1254429
C	-2.7603491	-2.2813134	-0.2555550
H	-2.8790124	-2.1666732	0.8060085
H	-1.7413517	-0.4091407	-0.8445577
H	-1.7264922	-1.4502893	-3.2978664
H	-2.9245782	-3.8357765	-3.1846026
H	-3.6141036	-4.3016724	-0.6545249

*Cp2TiCl2*L3 (Fig. 13, entry 3):*

77

H	0.2915812	2.2220907	-2.4556333
N	0.2233532	1.3688749	-1.9142881
S	-0.1385842	1.7738162	-0.3116031
O	0.5740629	3.0247687	-0.0231818
O	0.0853800	0.5578031	0.4892935
C	-1.9096092	2.0838331	-0.2635524
C	-2.3745454	3.3816903	-0.3643296
C	-3.7489513	3.5902398	-0.3897337
C	-4.6341149	2.5229984	-0.3349302
C	-4.1348166	1.2284675	-0.2454551
C	-2.7689122	0.9985318	-0.1949615
H	-2.3730404	-0.0008124	-0.1077942
C	-5.0815092	0.0641622	-0.1548768
F	-5.3817742	-0.2342870	1.1375556
F	-4.5549850	-1.0569942	-0.7013380
F	-6.2536831	0.3018443	-0.7845222
H	-5.6976029	2.6913034	-0.3705023
C	-4.2535520	5.0060118	-0.4495547
F	-3.9974504	5.6712996	0.7077759
F	-3.6528580	5.7114793	-1.4417446
F	-5.5846274	5.0804395	-0.6611235
H	-1.6823393	4.2076254	-0.4140698
C	-0.5232975	0.3308635	-2.5438247
C	-1.6788456	0.6270827	-3.2591104
C	-2.3888636	-0.3729366	-3.9029426
C	-1.9292983	-1.6814317	-3.8397664
C	-0.7637704	-1.9878681	-3.1565758
C	-0.0529332	-0.9926297	-2.4863270
N	1.1499475	-1.2664589	-1.8157599
S	1.3740726	-2.6386964	-0.8440614
O	1.3935014	-3.8182597	-1.7223060
O	2.5271625	-2.3032864	0.0057836
C	-0.0896167	-2.7789584	0.1943161
C	-0.1082046	-2.1025399	1.4032656
C	-1.2582407	-2.1485464	2.1713053
C	-2.3700952	-2.8705438	1.7460144

C	−2.3142873	−3.5605321	0.5458138
C	−1.1692795	−3.5240528	−0.2440266
H	−1.1235969	−4.0673944	−1.1727351
C	−3.4720945	−4.4083150	0.0947970
F	−3.7177236	−4.2698008	−1.2303623
F	−3.2238065	−5.7300758	0.3063501
F	−4.6183390	−4.1167333	0.7461171
H	−3.2657259	−2.8836826	2.3458838
C	−1.3488400	−1.3711913	3.4547213
F	−1.8288621	−2.1274753	4.4733553
F	−0.1542924	−0.8827206	3.8520898
F	−2.1904063	−0.3110123	3.3294547
H	0.7545102	−1.5443846	1.7234291
H	1.5950768	−0.4564146	−1.3899090
H	−0.3902758	−2.9984531	−3.1566982
H	−2.4690168	−2.4736275	−4.3394862
H	−3.2925011	−0.1325034	−4.4449637
H	−2.0173385	1.6527732	−3.2853636
Cl	6.4578602	2.7309178	−0.1852728
Ti	5.2994516	0.7177306	0.3074482
Cl	3.6246334	1.1076201	−1.3270105
C	6.5781067	−0.2363868	−1.4733170
C	7.4461582	−0.1094852	−0.3693921
C	6.9684224	−0.9335022	0.6629781
C	5.8061230	−1.5937518	0.1876830
C	5.5804193	−1.1713327	−1.1320906
H	4.7480532	−1.4607075	−1.7459188
H	5.2018078	−2.2962326	0.7304201
H	7.4087005	−1.0457986	1.6365357
H	8.2911118	0.5518245	−0.3055536
H	6.6326585	0.3260105	−2.3887330
C	3.9321370	2.0510587	1.7465136
C	3.3217976	0.7848309	1.6500831
C	4.1908366	−0.1670021	2.2048387
C	5.3437869	0.5166155	2.6705387
C	5.1706437	1.8845202	2.3982123
H	5.8880203	2.6604561	2.5940266
H	6.1938263	0.0704041	3.1536278
H	4.0056930	−1.2232163	2.2646859
H	2.3789033	0.5802582	1.1802754
H	3.5440059	2.9680097	1.3399264

*Cp2TiCl2-*L1 (Fig. 10, entry 1):*

63

S	−0.0848429	3.4536451	−0.2407508
C	−0.0200604	1.7864968	−0.0595212
N	−1.1077455	0.9591329	−0.1148383
C	−2.4611715	1.2510316	−0.0625363
C	−2.9943678	2.3595803	0.5934303
C	−4.3709682	2.5376528	0.6287069
C	−5.2394295	1.6307170	0.0399857
C	−4.6970359	0.5142692	−0.5872532
C	−3.3289573	0.3223793	−0.6472546
H	−2.9102332	−0.5372403	−1.1490325
C	−5.6362026	−0.4897024	−1.1850378
F	−5.0016917	−1.4730534	−1.8637793
F	−6.5121198	0.0839391	−2.0531835
F	−6.3938109	−1.1001284	−0.2307725
H	−6.3062101	1.7802157	0.0770021
C	−4.9245945	3.7766044	1.2695901
F	−6.1782503	3.6015169	1.7540971
F	−4.1636232	4.2139146	2.3030322
F	−4.9973359	4.8093898	0.3810650
H	−2.3404151	3.0656883	1.0733431
H	−0.8956464	0.0013467	−0.4224236
N	1.1041768	1.0446895	0.1481148
C	2.4552736	1.3344352	0.0362897
C	2.9877071	2.5093134	−0.4929654
C	4.3660152	2.6633997	−0.5712588
C	5.2395298	1.6797003	−0.1364495
C	4.6976926	0.5085298	0.3838123
C	3.3310665	0.3289202	0.4693034
H	2.9163231	−0.5802899	0.8793732
C	5.6311384	−0.5862454	0.8078011
F	6.1662606	−1.2328543	−0.2664328
F	6.6808928	−0.1148352	1.5288876
F	5.0292733	−1.5305776	1.5666560
H	6.3065907	1.8151275	−0.1992742
C	4.9183494	3.9656515	−1.0725479
F	4.1139639	4.5558044	−1.9896627
F	6.1371893	3.8305000	−1.6495507
F	5.0711783	4.8657549	−0.0583310
H	2.3261858	3.2884706	−0.8268471
H	0.9209361	0.0980819	0.5043260
Cl	−0.3724426	−1.6995490	−1.6357736
Ti	0.0174420	−3.5332663	0.0784486
Cl	0.5527645	−1.6607019	1.7162789
C	−2.3661705	−3.7107943	−0.3102950
C	−1.8381584	−5.0099200	−0.3343671
C	−1.3143180	−5.2885728	0.9519528
C	−1.4972927	−4.1517924	1.7583591
C	−2.1435958	−3.1677929	0.9688922
H	−2.3714930	−2.1641225	1.2792916
H	−1.1716846	−4.0324868	2.7767476
H	−0.8340045	−6.2031351	1.2521738
H	−1.8172109	−5.6677643	−1.1868751

H	-2.7885057	-3.1926241	-1.1509478
C	1.4449239	-4.3976018	-1.5656147
C	2.1832908	-3.3984917	-0.8831157
C	2.3981155	-3.8370015	0.4352954
C	1.7772048	-5.0860091	0.5913456
C	1.2038573	-5.4417556	-0.6540364
H	0.6566103	-6.3444290	-0.8600508
H	1.7317167	-5.6608393	1.5011158
H	2.8776950	-3.2762026	1.2155093
H	2.4742674	-2.4466351	-1.2890417
H	1.1039697	-4.3480386	-2.5850197

*Cp2TiCl2-*L2 (Fig. 10, entry 2):*

67

H	-1.1653819	0.2388645	0.4253735
N	-1.5658848	1.1472167	0.1508850
C	-0.7094029	2.1681403	0.0395277
C	0.7001657	2.1675056	-0.0435184
N	1.5561717	1.1471901	-0.1614316
C	2.9477069	1.1919729	-0.1126628
C	3.6465865	2.2721041	0.4214650
C	5.0321288	2.2446984	0.4463360
C	5.7479899	1.1549622	-0.0293263
C	5.0375338	0.0785094	-0.5455758
C	3.6533793	0.0872623	-0.5921780
H	3.1033115	-0.7396317	-1.0153760
C	5.7817138	-1.1398002	-1.0080966
F	7.0293642	-0.8516649	-1.4504901
F	5.9287712	-2.0477973	-0.0027129
F	5.1459803	-1.7829669	-2.0184358
H	6.8248049	1.1421472	-0.0009256
C	5.7631449	3.4571866	0.9457244
F	5.8901953	4.3995372	-0.0308988
F	7.0172728	3.1736239	1.3719313
F	5.1230726	4.0589092	1.9778554
H	3.1165808	3.1369362	0.7824686
H	1.1551198	0.2398000	-0.4402247
C	0.7531662	3.6483073	-0.0498873
O	1.6090514	4.5139507	-0.1029040
C	-0.7607705	3.6488359	0.0507302
O	-1.6156004	4.5156710	0.1045315
C	-2.9570767	1.1893022	0.0986550
C	-3.6587349	2.2779353	-0.4149323
C	-5.0438221	2.2450657	-0.4459898
C	-5.7576060	1.1432475	0.0054220
C	-5.0445802	0.0603091	0.5035763
C	-3.6604191	0.0730039	0.5535616
H	-3.1084181	-0.7603206	0.9618500
C	-5.7807035	-1.1703971	0.9456409
F	-7.0488526	-0.9056171	1.3412160
F	-5.8722245	-2.0867796	-0.0583242
F	-5.1681892	-1.7945759	1.9821675
H	-6.8345600	1.1295595	-0.0280512
C	-5.7785345	3.4646910	-0.9220172

```
F    -7.0252438    3.1827124   -1.3706946
F    -5.9251614    4.3794692    0.0778771
F    -5.1313420    4.0987757   -1.9297662
H    -3.1311368    3.1532250   -0.7540979
Cl   -0.5086664   -1.4408299    1.6493865
Ti    0.0064618   -3.3283779    0.0184522
Cl    0.4682690   -1.4399067   -1.6309719
C    -2.3671759   -3.6305803    0.3494501
C    -2.1406935   -3.1194251   -0.9416889
C    -1.4174508   -4.0908301   -1.6787486
C    -1.1974243   -5.1890993   -0.8290726
C    -1.7702848   -4.8970413    0.4321174
H    -1.7386290   -5.5238446    1.3070111
H    -0.6609161   -6.0862927   -1.0824843
H    -1.0696279   -3.9884599   -2.6918973
H    -2.4170766   -2.1430128   -1.2961917
H    -2.8416837   -3.1152244    1.1634071
C     2.1527966   -3.0419194    0.9503300
C     1.4639456   -4.0100469    1.7235515
C     1.2628952   -5.1377207    0.9092404
C     1.8161025   -4.8689717   -0.3659110
C     2.3796334   -3.5856111   -0.3281626
H     2.8325637   -3.0833956   -1.1623091
H     1.7916716   -5.5220204   -1.2215760
H     0.7500511   -6.0394453    1.1942035
H     1.1230494   -3.8860948    2.7365764
H     2.4068679   -2.0487217    1.2735499
```

*Cp2TiCl2-*L3 (Fig. 10, entry 3):*

```
77

H     0.0321206   -1.5722365    1.8184360
N    -0.8516793   -1.6248762    2.3345753
C    -1.4754017   -0.3777212    2.6214147
C    -2.0777218   -0.2092200    3.8605494
C    -2.7518427    0.9615281    4.1666729
C    -2.8046949    1.9800777    3.2264708
C    -2.1910272    1.8367474    1.9922152
C    -1.5267743    0.6576963    1.6657032
N    -0.9304442    0.4047146    0.4181179
S    -1.0000495    1.3723742   -0.9481025
O    -2.3591426    1.9137198   -1.0815005
O    -0.3958795    0.5412672   -2.0007245
C     0.1375503    2.7365084   -0.6484846
C     1.4962690    2.4846944   -0.7638618
C     2.3823451    3.5281607   -0.5414047
C     1.9199917    4.7881966   -0.1870323
C     0.5533732    5.0065085   -0.0666896
C    -0.3549651    3.9841618   -0.3097566
H    -1.4175587    4.1534322   -0.2429416
C     0.0761421    6.3864592    0.2818008
F    -1.2340666    6.4194519    0.6114428
F     0.2432202    7.2516870   -0.7557868
F     0.7603502    6.9097338    1.3298364
H     2.6167401    5.5926385   -0.0131182
```

C	3.8644189	3.2930720	-0.6536584
F	4.1584514	2.2301013	-1.4246655
F	4.4276748	3.0817138	0.5621857
F	4.5019937	4.3661500	-1.1931129
H	1.8522453	1.4985634	-1.0167902
H	-0.0032843	-0.0463341	0.4648865
H	-2.2569154	2.6373631	1.2768869
H	-3.3192040	2.9041538	3.4505658
H	-3.2216351	1.0792949	5.1329916
H	-2.0169866	-1.0214737	4.5694519
S	-1.8340613	-2.8789512	1.7609528
O	-0.9087635	-3.9326609	1.3120460
O	-2.8511512	-3.1437567	2.7911893
C	-2.7053528	-2.2145590	0.3335959
C	-3.8051580	-1.4014421	0.5538645
C	-4.4100548	-0.8006585	-0.5414948
C	-3.9462902	-1.0357498	-1.8273390
C	-2.8557217	-1.8741798	-2.0183006
C	-2.2181077	-2.4645564	-0.9377423
H	-1.3502348	-3.0895909	-1.0731834
C	-2.4045538	-2.1659537	-3.4215247
F	-3.2067954	-3.1062028	-4.0050698
F	-1.1452641	-2.6451443	-3.4774844
F	-2.4577778	-1.0738778	-4.2181286
H	-4.4095548	-0.5507345	-2.6705584
C	-5.6149595	0.0732086	-0.3375062
F	-5.6062146	0.6946285	0.8635986
F	-5.7277290	1.0332151	-1.2826512
F	-6.7675795	-0.6570416	-0.3881062
H	-4.1664421	-1.2262792	1.5540569
Cl	1.9355625	-0.5654677	1.1029693
Ti	3.2587263	-2.6536776	0.3446836
Cl	5.1583609	-1.0738530	0.0625352
C	2.9442489	-2.0765392	-1.9313534
C	1.6587923	-2.3824592	-1.4522393
C	1.6400646	-3.7317567	-1.0676284
C	2.9168694	-4.2756188	-1.3442685
C	3.7286461	-3.2541974	-1.8706390
H	4.7678802	-3.3369954	-2.1364790
H	3.2230529	-5.2894167	-1.1556357
H	0.8161907	-4.2478335	-0.6070209
H	0.8556033	-1.6788340	-1.3436087
H	3.2915506	-1.1027015	-2.2244877
C	3.6586206	-2.7571913	2.6822916
C	2.5108533	-3.5366785	2.3931699
C	2.9265028	-4.6296631	1.6138324
C	4.3250311	-4.5250306	1.4125838
C	4.7719783	-3.3811516	2.0920275
H	5.7740601	-2.9935444	2.0792535
H	4.9347776	-5.1893739	0.8232902
H	2.2850928	-5.3966668	1.2170884
H	1.5004920	-3.3257867	2.6943803
H	3.6684292	-1.8167630	3.2036753

*Cp2TiCl2-*L3 (Fig. 10, entry 4):*

77

H	3.4680526	2.4219320	0.0926981
N	3.6046854	2.3827726	-0.9148040
C	2.3829019	2.3082545	-1.6387991
C	2.3033109	2.9282089	-2.8795640
C	1.1259874	2.8883033	-3.6110162
C	0.0083473	2.2494836	-3.0907541
C	0.0712973	1.6368437	-1.8492141
C	1.2579507	1.6449548	-1.1242270
N	1.3534942	0.8935176	0.0755871
S	1.2626571	1.6383076	1.5746147
O	2.1263548	2.8409167	1.5023799
O	1.5215685	0.5923521	2.5731143
C	-0.4197135	2.2201419	1.8000715
C	-1.2610479	1.5132578	2.6435503
C	-2.5590092	1.9646438	2.8076103
C	-3.0171503	3.0850518	2.1241460
C	-2.1603675	3.7537208	1.2647492
C	-0.8448586	3.3348920	1.1011198
H	-0.1744063	3.8662168	0.4440453
C	-2.6215383	4.9699276	0.5156626
F	-2.0542224	6.1071251	1.0085448
F	-3.9614403	5.1484093	0.5758289
F	-2.2883199	4.9153162	-0.7978579
H	-4.0354721	3.4145362	2.2516612
C	-3.4936200	1.2676980	3.7519843
F	-3.6946323	2.0064999	4.8837131
F	-3.0306598	0.0634052	4.1560021
F	-4.7183586	1.0628788	3.2110402
H	-0.8995951	0.6329706	3.1503373
H	0.7736056	0.0303418	0.0643885
H	-0.7955805	1.1459649	-1.4262265
H	-0.9185862	2.2334100	-3.6476943
H	1.0786405	3.3733965	-4.5765396
H	3.1780126	3.4354594	-3.2589273
S	4.8324148	1.2707820	-1.2797046
O	5.0787787	1.3468397	-2.7284875
O	5.9009033	1.5528792	-0.3053625
C	4.1635464	-0.3614460	-0.9266080
C	4.1178192	-0.7901715	0.3923158
C	3.5575085	-2.0254881	0.6632823
C	3.0387646	-2.8136345	-0.3605480
C	3.1008191	-2.3596985	-1.6663610
C	3.6639493	-1.1227746	-1.9648657
H	3.7091245	-0.7597074	-2.9796474
C	2.6074608	-3.2145368	-2.7969854
F	3.6542881	-3.7615781	-3.4883164
F	1.8876852	-2.5082624	-3.7000441
F	1.8398224	-4.2455028	-2.3873458
H	2.5769051	-3.7601941	-0.1319649
C	3.4970275	-2.5534405	2.0681460
F	4.1920739	-3.7262328	2.1803794
F	4.0317632	-1.7037874	2.9727124

F	2.2343046	-2.8220077	2.4632591
H	4.4984459	-0.1651766	1.1841144
Cl	-0.2212261	-1.7749504	-0.2389331
Ti	-2.6229695	-1.9478896	-1.0224365
Cl	-2.9256711	0.4215734	-0.2924766
C	-3.0990191	-2.4564615	1.2190463
C	-2.5417345	-3.6338321	0.6722055
C	-3.4230181	-4.1144019	-0.3058045
C	-4.5404652	-3.2449440	-0.3562112
C	-4.3454667	-2.2296040	0.5919290
H	-4.9895187	-1.3851623	0.7595090
H	-5.3739936	-3.3239988	-1.0338560
H	-3.2582897	-4.9733171	-0.9340336
H	-1.5732400	-4.0375115	0.9064563
H	-2.6235542	-1.8072046	1.9312699
C	-2.6890306	-0.7476900	-3.1468639
C	-1.4866004	-1.4648648	-3.0968678
C	-1.7943766	-2.8446506	-3.0333259
C	-3.1994674	-2.9649976	-3.0600881
C	-3.7569405	-1.6652893	-3.1001042
H	-4.8060028	-1.4207319	-3.0874456
H	-3.7549084	-3.8853472	-3.0376698
H	-1.0826971	-3.6486527	-2.9587973
H	-0.5024530	-1.0394469	-3.0329703
H	-2.7853802	0.3220324	-3.1209259

*Cp2TiCl*L1 (Fig. S20, left entry 1):*

62

S	-0.9677908	4.2553882	1.0781022
C	0.0514407	2.9911901	0.6390848
N	-0.3718861	1.7219163	0.3959220
C	-1.7199819	1.3315815	0.3860022
C	-2.6147816	1.9289773	-0.4969583
C	-3.9411117	1.5324805	-0.4904931
C	-4.3905765	0.5411693	0.3758476
C	-3.4841627	-0.0535649	1.2388829
C	-2.1469106	0.3295991	1.2475996
H	-1.4429780	-0.1251318	1.9267948
C	-3.9721680	-1.1385513	2.1515476
F	-5.1588650	-0.8282480	2.7292378
F	-3.1050668	-1.4080784	3.1545022
F	-4.1703241	-2.3105285	1.4804640
H	-5.4282808	0.2475813	0.3788368
C	-4.9416205	2.1885802	-1.3963614
F	-4.3746837	3.0341354	-2.2865905
F	-5.6524520	1.2790447	-2.1148499
F	-5.8523742	2.9149815	-0.6913481
H	-2.2651927	2.6946725	-1.1686769
H	0.3021896	0.9689200	0.5322676
N	1.3939819	3.1868187	0.5664179
C	2.3648958	2.3961189	-0.0659805
C	3.6194369	2.2893942	0.5280125
C	4.6150173	1.5589298	-0.1030883
C	4.3827579	0.9291187	-1.3170286

C	3.1302119	1.0583349	−1.9041460
C	2.1221334	1.7873876	−1.2963916
H	1.1590649	1.8936382	−1.7676986
C	2.9155989	0.4307414	−3.2502008
F	3.6661906	1.0362201	−4.2116450
F	3.2716153	−0.8809665	−3.2617538
F	1.6303511	0.4921676	−3.6654363
H	5.1602822	0.3603198	−1.8013430
C	5.9436179	1.3733541	0.5700441
F	6.9700849	1.3289179	−0.3149754
F	5.9881942	0.2056771	1.2707837
F	6.2267651	2.3627424	1.4507118
H	3.7964069	2.7710856	1.4769646
H	1.7217463	4.0272363	1.0177514
Cl	1.4866000	−0.9115810	0.9664711
Ti	0.1759389	−2.8377277	0.4780503
C	−0.1923473	−1.7355439	−1.5429839
C	0.6108949	−2.8619113	−1.8290824
C	−0.1488501	−4.0129419	−1.5491551
C	−1.4203279	−3.5998658	−1.0915883
C	−1.4484878	−2.1919320	−1.0815812
H	−2.2819596	−1.5785771	−0.7877234
H	−2.2169311	−4.2457179	−0.7658882
H	0.1934111	−5.0308317	−1.6275115
H	1.6342644	−2.8438086	−2.1629928
H	0.1123721	−0.7092628	−1.6309069
C	−0.3695845	−4.9166895	1.3765115
C	1.0277356	−4.7435628	1.5150691
C	1.2347381	−3.6802803	2.4205470
C	−0.0221018	−3.1832304	2.8158716
C	−1.0186792	−3.9396914	2.1649587
H	−2.0817035	−3.7994668	2.2497923
H	−0.1880572	−2.3303034	3.4526606
H	2.1885580	−3.2693157	2.7024108
H	1.7920464	−5.3245045	1.0264709
H	−0.8554255	−5.6463466	0.7544173

*Cp2TiCl*L2 (Fig. S20, left entry 2):*

66

H	-0.6360639	0.8592568	0.5546046
N	-1.6258234	1.0862617	0.5912574
C	-1.9616476	2.2198128	1.2285586
C	-1.1938016	3.3358929	1.6128487
N	0.0568487	3.7691408	1.4432324
C	0.9629391	3.3332499	0.4698066
C	0.5160383	2.8566904	-0.7560325
C	1.4349980	2.3721645	-1.6782018
C	2.7927069	2.3946933	-1.4156798
C	3.2262223	2.9221905	-0.2012434
C	2.3308256	3.3902987	0.7419140
H	2.6688779	3.7712162	1.6925645
C	4.6990361	2.9046966	0.0896053
F	5.1631512	1.6334701	0.2503955
F	5.4235918	3.4483407	-0.9238001
F	5.0270308	3.5816018	1.2125716
H	3.5013361	2.0065365	-2.1282803
C	0.9108110	1.8461634	-2.9824102
F	1.8551803	1.2144747	-3.7146214
F	-0.1023387	0.9582957	-2.7979733
F	0.4076768	2.8418249	-3.7620362
H	-0.5351304	2.8592550	-0.9914505
H	0.3679233	4.4966249	2.0728635
C	-2.3023893	3.9124532	2.3915958
O	-2.4354511	4.8795102	3.1163828
C	-3.1578654	2.7241401	1.9487434
O	-4.2998770	2.3511475	2.1336389
C	-2.4713912	0.1105211	0.0658068
C	-3.8157250	0.3502296	-0.2063874
C	-4.5913525	-0.6617340	-0.7524146
C	-4.0578950	-1.9069834	-1.0560867
C	-2.7122713	-2.1247376	-0.7940160
C	-1.9204547	-1.1341061	-0.2370032
H	-0.8759702	-1.3168096	-0.0255906
C	-2.0725602	-3.4424821	-1.1185528
F	-1.1099005	-3.3108388	-2.0719431
F	-2.9500368	-4.3615480	-1.5721407
F	-1.4585578	-3.9854880	-0.0313026
H	-4.6707919	-2.6810610	-1.4861327
C	-6.0448698	-0.3741688	-1.0036512
F	-6.7205271	-0.1620014	0.1568066
F	-6.2149311	0.7444846	-1.7572187
F	-6.6780704	-1.3795383	-1.6473160
H	-4.2547508	1.3056161	0.0262592
Cl	1.4206714	-0.0468921	1.0857970
Ti	2.3513909	-2.2351214	1.0098817
C	1.0324770	-2.6652498	2.9258240
C	2.2493192	-2.1013507	3.3596883
C	3.2970286	-2.9784541	3.0066230
C	2.7145731	-4.0986337	2.3678057
C	1.3156475	-3.8959316	2.3007055
H	0.6006536	-4.5643297	1.8518463

H	3.2426805	-4.9567382	1.9963826
H	4.3475974	-2.8305361	3.1946039
H	2.3643112	-1.1315066	3.8126059
H	0.0641986	-2.1986008	2.9935377
C	3.4918034	-3.6902828	-0.4185695
C	4.2127910	-2.4732102	-0.3710599
C	3.4007793	-1.4796498	-0.9593851
C	2.1836481	-2.0728420	-1.3475202
C	2.2291670	-3.4385105	-1.0043321
H	1.4468149	-4.1614571	-1.1604642
H	1.3393130	-1.5552604	-1.7693594
H	3.6327611	-0.4312787	-1.0258204
H	5.2020429	-2.3331897	0.0321622
H	3.8418158	-4.6437149	-0.0685815

*Cp2TiCl*L3 (Fig. 11, entry 3; Fig. S20, left entry 3):*

76

Cl	-0.6322794	3.1022554	-1.6717001
Ti	-1.9454231	3.3143832	0.4398898
O	-1.3265649	1.1914151	0.8846958
S	-0.9902380	-0.1637246	0.3704474
O	-0.1638924	-1.0372810	1.2140923
N	-0.3747464	0.0266344	-1.1707874
C	0.5653192	-0.8933388	-1.6771921
C	0.2163835	-2.2342906	-1.8167844
C	1.1032478	-3.1444394	-2.3647932
C	2.3503247	-2.7205188	-2.8054487
C	2.7097632	-1.3904492	-2.6679731
C	1.8359583	-0.4696438	-2.0925929
N	2.2178076	0.8956260	-1.9817186
S	2.8084657	1.4441421	-0.4740690
O	1.7236049	1.3505041	0.5090565
O	3.4813005	2.7210546	-0.7548975
C	4.0311756	0.2180701	0.0188594
C	3.5834659	-0.9220473	0.6649589
C	4.5096708	-1.8967375	1.0090914
C	5.8591027	-1.7267163	0.7265908
C	6.2765615	-0.5686894	0.0827264
C	5.3649741	0.4128966	-0.2881226
H	5.6862708	1.3131245	-0.7888197
C	7.7309987	-0.3332532	-0.2167698
F	8.1995579	0.7683461	0.4259047
F	7.9418975	-0.1246440	-1.5429732
F	8.5176428	-1.3677907	0.1494689
H	6.5730021	-2.4834223	1.0073823
C	4.0106758	-3.1323257	1.7055776
F	3.4118382	-2.8288615	2.8869764
F	3.0822559	-3.7824358	0.9585774
F	4.9934428	-4.0177897	1.9763995
H	2.5350051	-1.0351237	0.9005435
H	2.9103365	1.1587610	-2.6738893
H	3.6847244	-1.0468222	-2.9833869
H	3.0432196	-3.4232061	-3.2466763
H	0.8098279	-4.1798622	-2.4645968

H	-0.7683852	-2.5549944	-1.5127110
H	-0.2223474	1.0310164	-1.3615244
C	-2.5619295	-1.0082464	0.1285769
C	-3.1882214	-1.4944234	1.2647526
C	-4.4279090	-2.1024517	1.1246877
C	-5.0223722	-2.2359079	-0.1244567
C	-4.3634454	-1.7430521	-1.2425604
C	-3.1272272	-1.1185861	-1.1266036
H	-2.6148545	-0.7312516	-1.9922431
C	-4.9487896	-1.9199407	-2.6168042
F	-4.3014180	-2.9005630	-3.3041682
F	-4.8438152	-0.7926232	-3.3616542
F	-6.2558520	-2.2583336	-2.5885995
H	-5.9827570	-2.7144389	-0.2236668
C	-5.1097633	-2.6110694	2.3658947
F	-4.3434359	-3.5214805	3.0200163
F	-5.3515950	-1.6048779	3.2470037
F	-6.2948307	-3.2035514	2.1085456
H	-2.7227103	-1.4022017	2.2332877
C	-3.6681726	2.3875543	-0.8312221
C	-3.6687278	3.7744939	-1.1095565
C	-4.0185266	4.4484467	0.0689203
C	-4.2480216	3.4826589	1.0798166
C	-4.0465598	2.2129680	0.5200030
H	-4.1123794	1.2751501	1.0429443
H	-4.4957705	3.6884823	2.1071656
H	-4.0707479	5.5166439	0.1904637
H	-3.3898048	4.2302499	-2.0428350
H	-3.3938161	1.6137305	-1.5246593
C	0.1267616	4.3565832	1.0575317
C	-0.2010277	3.4853395	2.1047766
C	-1.4263119	3.9019692	2.6654201
C	-1.8357260	5.0658105	1.9732696
C	-0.8894478	5.3353357	0.9584075
H	-0.9356292	6.1347811	0.2378788
H	-2.7229830	5.6371905	2.1759883
H	-1.9545794	3.4223308	3.4728379
H	0.3455741	2.5974301	2.3653880
H	0.9666856	4.2466454	0.3968940

*Cp2TiCl(THF)*L1 (Fig. 11, entry 1; Fig. S20, right entry 1):*

75

S	0.4686580	-3.2690116	-0.7444134
C	0.3317961	-2.0178369	0.3553673
N	-0.8510845	-1.5536845	0.8649127
C	-2.1560183	-1.8959400	0.5084663
C	-2.5774876	-3.2083482	0.3044431
C	-3.9082846	-3.4520041	-0.0013024
C	-4.8359573	-2.4213858	-0.1011763
C	-4.4056343	-1.1226603	0.1253695
C	-3.0792843	-0.8561398	0.4298738
H	-2.7454675	0.1581786	0.5979488
C	-5.3791957	0.0176771	0.0917446
F	-4.8524271	1.1208935	-0.5047154

F	-5.7432403	0.4040010	1.3469281
F	-6.5181061	-0.2802983	-0.5695738
H	-5.8654682	-2.6301862	-0.3408499
C	-4.3515084	-4.8607578	-0.2760275
F	-5.6357367	-5.0813755	0.0948955
F	-4.2761418	-5.1572986	-1.6038726
F	-3.5925942	-5.7794509	0.3677301
H	-1.8759484	-4.0178831	0.3976719
H	-0.7917348	-0.6394531	1.3152120
N	1.3724272	-1.2660496	0.8267753
C	2.7302142	-1.3302847	0.5247719
C	3.4192346	-2.5245523	0.3071051
C	4.7811896	-2.4852270	0.0583048
C	5.4868603	-1.2863367	0.0277496
C	4.7954703	-0.1112849	0.2704860
C	3.4300935	-0.1268010	0.5188317
H	2.8964523	0.7949852	0.7046608
C	5.5022526	1.2117746	0.2760524
F	5.4708097	1.7946185	1.5058418
F	6.7999006	1.1215491	-0.0829244
F	4.9170569	2.0969760	-0.5773910
H	6.5461186	-1.2780487	-0.1698534
C	5.5282292	-3.7582896	-0.2189032
F	5.7876057	-3.9057069	-1.5479890
F	6.7292932	-3.7929078	0.4114126
F	4.8491106	-4.8619955	0.1681612
H	2.8934071	-3.4611460	0.3435984
H	1.0992035	-0.3869107	1.2678203
Cl	-0.1340371	1.4789120	1.6595895
Ti	-0.0048232	3.1182346	-0.2887164
O	-2.0826874	3.6031995	0.3121712
C	-2.9217167	4.4443468	-0.5328163
C	-4.1124140	4.8280352	0.3207759
H	-4.5072090	5.8022469	0.0444507
C	-3.5413879	4.7880873	1.7317315
H	-4.3025132	4.6661639	2.4980992
C	-2.6132181	3.5994784	1.6700801
H	-1.7790223	3.6461588	2.3567671
H	-3.1412410	2.6590304	1.8211283
H	-2.9807066	5.6973191	1.9451550
H	-4.9100047	4.0947219	0.2138308
H	-3.1895018	3.8751009	-1.4151148
H	-2.3340413	5.3101766	-0.8307393
C	0.2240866	5.3789156	0.4993458
C	0.7790819	4.5063624	1.4553322
C	1.9002604	3.8698129	0.8680786
C	2.0207802	4.3440123	-0.4476310
C	0.9715183	5.2668073	-0.6836434
H	0.7696979	5.7731740	-1.6125179
H	2.7644538	4.0362443	-1.1602116
H	2.5321294	3.1408874	1.3437633
H	0.4081052	4.3347015	2.4498187
H	-0.6583190	5.9804037	0.6317897
C	-0.7420918	3.0029252	-2.5139982
C	-1.3030501	1.8653512	-1.8879166

C	−0.2561304	1.0202544	−1.4979041
C	0.9644020	1.6203101	−1.8632375
C	0.6593853	2.8354309	−2.5179523
H	1.3728203	3.5188961	−2.9395077
H	1.9483181	1.2188692	−1.6906389
H	−0.3669744	0.0897607	−0.9771262
H	−2.3472140	1.6991965	−1.6911217
H	−1.2806802	3.8346599	−2.9344320

*Cp2TiCl(THF)*L2 (Fig. 11, entry 2; Fig. S20, right entry 2):*

79

H	−1.1716508	−0.4619317	−0.8261890
N	−1.6252775	−1.3141005	−0.4873437
C	−0.8111256	−2.2839875	−0.0505192
C	0.6008528	−2.3338644	−0.0333118
N	1.4895862	−1.4194950	−0.4429288
C	2.8821810	−1.4872630	−0.4803782
C	3.6076489	−2.5790277	−0.0140924
C	4.9923054	−2.5618460	−0.0933910
C	5.6801869	−1.4838772	−0.6303827
C	4.9422043	−0.3984104	−1.0868668
C	3.5592782	−0.3899895	−1.0172750
H	2.9994825	0.4605165	−1.3775216
C	5.6437565	0.7604380	−1.7346102
F	5.0605967	1.9492125	−1.4356256
F	5.6286620	0.6565662	−3.0923620
F	6.9410220	0.8567900	−1.3669059
H	6.7559160	−1.4845094	−0.6862907
C	5.7336776	−3.7614101	0.4280128
F	5.3804658	−4.8949965	−0.2332412
F	5.4649681	−3.9834255	1.7418117
F	7.0746887	−3.6429377	0.3162788
H	3.0959068	−3.4332314	0.4063350
H	1.1006696	−0.5302967	−0.7709631
C	0.5978696	−3.6881784	0.5661625
O	1.4177862	−4.5128473	0.9283521
C	−0.9161776	−3.6377201	0.5438229
O	−1.7989329	−4.4048151	0.8835180
C	−3.0193196	−1.2911607	−0.5502498
C	−3.6096872	−0.1970695	−1.1887172
C	−4.9864914	−0.1152133	−1.2837400
C	−5.8060666	−1.1075659	−0.7543291
C	−5.2050099	−2.1872679	−0.1291152
C	−3.8248468	−2.2895820	−0.0154581
H	−3.3809181	−3.1449204	0.4731619
C	−6.0352693	−3.3007837	0.4456979
F	−7.3636007	−3.0869208	0.3224448
F	−5.7683899	−4.4872667	−0.1613914
F	−5.7850021	−3.4817375	1.7693066
H	−6.8778593	−1.0359395	−0.8373752
C	−5.6318395	1.0806664	−1.9233230
F	−6.6812511	0.7347673	−2.7086003
F	−6.1177221	1.9432267	−0.9881359
F	−4.7767968	1.7881428	−2.6973214

H	-2.9835175	0.5748366	-1.6081979
Cl	0.0805845	1.3853278	-1.1108331
Ti	-0.3535202	2.7294256	1.0345501
O	1.1734095	4.1122249	0.2584696
C	2.3122940	3.7564343	-0.5776238
C	3.1585386	5.0073267	-0.6417559
H	4.2036737	4.7780944	-0.8326736
C	2.9219504	5.6314722	0.7273053
H	3.1481040	6.6942430	0.7618902
C	1.4510223	5.3623058	0.9545714
H	1.1846017	5.2354325	1.9979519
H	0.8202827	6.1340178	0.5145561
H	3.5234089	5.1272208	1.4826741
H	2.8023506	5.6695718	-1.4303330
H	1.9272505	3.4228251	-1.5342130
H	2.8400035	2.9350095	-0.0977970
C	-1.7235631	4.6057034	1.3912402
C	-2.3856629	3.4785206	1.9229301
C	-2.7129662	2.6106358	0.8554982
C	-2.2882919	3.2282827	-0.3383290
C	-1.6760564	4.4470446	-0.0134308
H	-1.1973336	5.1109161	-0.7126253
H	-2.3569751	2.8145357	-1.3272092
H	-3.1988924	1.6541710	0.9442552
H	-2.5968170	3.3090398	2.9623557
H	-1.3482537	5.4423795	1.9550566
C	1.3444099	1.2917677	1.8594711
C	0.0850879	0.7059787	2.1393066
C	-0.5744233	1.5348065	3.0619807
C	0.2657949	2.6409421	3.3431424
C	1.4561574	2.4707539	2.6177287
H	2.2841103	3.1567461	2.5883349
H	0.0263294	3.4717347	3.9857873
H	-1.5572478	1.3640273	3.4617166
H	-0.3039429	-0.2058246	1.7243261
H	2.0860806	0.9113724	1.1806977

*Cp2TiCl(THF)*L3 (Fig. S20, right entry 3):*

89

H	1.2848426	-0.4534081	1.8879862
N	1.6772024	-1.3882512	1.8168531
S	3.2620028	-1.3478963	2.4008385
O	3.2841817	-0.1740050	3.2855529
O	3.6092749	-2.6834283	2.9019169
C	4.3122609	-1.0127186	0.9707927
C	5.3310729	-1.8959665	0.6737101
C	6.1430312	-1.6256194	-0.4234979
C	5.9333060	-0.4986327	-1.2005483
C	4.8916924	0.3677670	-0.8786139
C	4.0733944	0.1252768	0.2134952
H	3.2569158	0.7922816	0.4588522
C	4.6927710	1.6013606	-1.7143602
F	5.5513041	2.5923250	-1.3495590
F	3.4446827	2.1023846	-1.6014102

F	4.9101128	1.3662083	-3.0307386
H	6.5570454	-0.2968379	-2.0559737
C	7.2493354	-2.5921800	-0.7440248
F	8.1758371	-2.6342314	0.2483902
F	6.7798983	-3.8583254	-0.8882661
F	7.9036692	-2.2821721	-1.8832491
H	5.4748389	-2.7860219	1.2657925
C	1.2610673	-2.1318553	0.6992765
C	2.0169097	-3.1649048	0.1490623
C	1.5537604	-3.8633017	-0.9555724
C	0.3357434	-3.5448576	-1.5385186
C	-0.4249067	-2.5239284	-0.9924320
C	0.0106694	-1.8311975	0.1290808
N	-0.8514926	-0.8624486	0.7047153
S	-2.0061348	-1.3574522	1.8279600
O	-1.6148889	-2.6305271	2.4516220
O	-2.3237417	-0.1644752	2.6329209
C	-3.4335454	-1.6975137	0.7804368
C	-4.3130772	-0.6679540	0.4977906
C	-5.3812071	-0.9159154	-0.3529135
C	-5.5670290	-2.1751158	-0.9108084
C	-4.6702325	-3.1895169	-0.6045506
C	-3.6002402	-2.9646432	0.2528567
H	-2.9039136	-3.7530088	0.4924495
C	-4.8321283	-4.5668355	-1.1872232
F	-3.7144475	-4.9610010	-1.8501316
F	-5.8593513	-4.6497673	-2.0598150
F	-5.0567735	-5.4935139	-0.2202527
H	-6.3952331	-2.3586148	-1.5750585
C	-6.3553719	0.1954428	-0.6262688
F	-7.1796794	-0.0684784	-1.6593923
F	-7.1388392	0.4449409	0.4565267
F	-5.7163416	1.3606961	-0.9130308
H	-4.1615669	0.3050386	0.9412941
H	-0.4729740	0.0643481	0.9305069
H	-1.3771956	-2.2490446	-1.4215454
H	-0.0178354	-4.0816229	-2.4073209
H	2.1628309	-4.6586039	-1.3621143
H	2.9610173	-3.4426512	0.5862428
Cl	0.8452009	1.7837389	1.1534845
Ti	-0.4054170	3.3924326	-0.3756155
O	-2.0257478	3.2015802	1.1218282
C	-3.3213604	3.8189612	0.8675951
C	-3.8692358	4.2090902	2.2236734
H	-3.5522929	5.2177608	2.4864912
C	-3.2170714	3.1900952	3.1484199
H	-3.7349503	2.2334410	3.0999625
H	-3.1878415	3.5113491	4.1865680
C	-1.8331643	3.0638967	2.5622456
H	-1.1748929	3.8632668	2.8987483
H	-1.3580235	2.1090827	2.7340195
H	-4.9557722	4.1781812	2.2440789
H	-3.9451881	3.0761347	0.3726817
H	-3.1762405	4.6591168	0.1965829
C	-0.4417702	5.5752586	0.6765959

C	-0.4528775	5.7569645	-0.7165877
C	0.7669727	5.2465249	-1.2266761
C	1.5068555	4.7183676	-0.1536276
C	0.7520613	4.9220909	1.0280463
H	1.0303665	4.5957695	2.0141026
H	2.4615278	4.2268112	-0.2159600
H	1.0668012	5.2417919	-2.2592736
H	-1.2478793	6.1981750	-1.2945709
H	-1.2416169	5.8279873	1.3506670
C	-0.2647564	1.4222549	-1.7671062
C	0.1333760	2.5191230	-2.5451210
C	-0.9784326	3.3853105	-2.6733969
C	-2.0552791	2.8327158	-1.9560030
C	-1.6067145	1.6158437	-1.3879021
H	-2.1744966	0.9608090	-0.7528554
H	-3.0419346	3.2531632	-1.8746122
H	-0.9947051	4.3152003	-3.2132262
H	1.1158608	2.6849205	-2.9517768
H	0.3678089	0.6087919	-1.4627675

*(Cp2TiCl)2*L2 (Fig. S21, entry 1):*

88

H	0.4885427	0.6485654	0.1749009
N	-0.0334429	1.4929607	0.3969129
C	-1.3668232	1.3718161	0.4697083
C	-2.1926086	0.2248812	0.5930582
N	-2.0294581	-1.0743664	0.8179822
C	-0.9013077	-1.7162624	1.3377622
C	-0.7104472	-3.0597796	1.0145088
C	0.3903516	-3.7277822	1.5129633
C	1.3151740	-3.0824923	2.3317148
C	1.0980033	-1.7564843	2.6597242
C	-0.0128003	-1.0695064	2.1845945
H	-0.1853638	-0.0490449	2.4826334
C	2.0331499	-1.0263043	3.5797049
F	3.1535172	-1.7269119	3.8595985
F	1.4429363	-0.7325005	4.7679145
F	2.4333543	0.1628621	3.0506084
H	2.1789639	-3.6108596	2.6995499
C	0.6373988	-5.1647869	1.1548679
F	-0.3627112	-5.7056838	0.4243901
F	0.7859484	-5.9423642	2.2587362
F	1.7781468	-5.3108590	0.4270329
H	-1.4251512	-3.5586466	0.3783306
H	-2.8906292	-1.6409162	0.7457534
C	-3.3972007	0.9940421	0.3336719
O	-4.6026895	0.7553743	0.1410979
Ti	-5.7521099	-0.7639630	-0.7727859
Cl	-4.7823311	-2.4628809	0.7921223
C	-8.0391838	-0.5638518	-1.2331167
C	-7.8980904	-1.5418418	-0.2264873
C	-7.4409923	-0.8884484	0.9410895
C	-7.2857125	0.4744004	0.6494798
C	-7.6290366	0.6816819	-0.7015985

H	-7.5808204	1.6166440	-1.2345559
H	-6.8841150	1.2166297	1.3170082
H	-7.1949372	-1.3641821	1.8730019
H	-8.0868899	-2.5972443	-0.3306007
H	-8.3820721	-0.7387038	-2.2361425
C	-4.6790701	-2.2428865	-2.3300900
C	-3.8616920	-1.1052239	-2.1541462
C	-4.5554979	0.0189235	-2.6599863
C	-5.8034687	-0.4239089	-3.1238680
C	-5.8816416	-1.8248162	-2.9149384
H	-6.7286637	-2.4515638	-3.1360219
H	-6.5770330	0.1975783	-3.5388241
H	-4.2115273	1.0394334	-2.6493403
H	-2.8781710	-1.0988407	-1.7186526
H	-4.4445658	-3.2420091	-2.0097354
C	-2.5598920	2.2452615	0.2652924
O	-2.7765157	3.4284086	0.0915981
C	0.7359113	2.6472761	0.5081449
C	2.0888738	2.5642958	0.1795666
C	2.9017225	3.6772940	0.3151729
C	2.3945287	4.8879155	0.7689206
C	1.0450415	4.9541277	1.0914085
C	0.2129855	3.8525311	0.9732925
H	-0.8320997	3.9319017	1.2220678
C	0.4439814	6.2572460	1.5375230
F	-0.1972603	6.8842789	0.5130731
F	1.3680892	7.1258244	2.0070155
F	-0.4747605	6.0875918	2.5205537
H	3.0324446	5.7494814	0.8737044
C	4.3611639	3.5352806	-0.0032082
F	5.0119548	4.7157653	-0.0429726
F	5.0003368	2.7629781	0.9199868
F	4.5604820	2.9318165	-1.2055862
H	2.4915038	1.6288328	-0.1838348
Cl	1.5723911	-0.9879861	-1.0575625
Ti	3.7991138	-1.1773206	-1.9034205
C	5.0200880	-0.9736482	-3.8853756
C	3.9230479	-1.8305446	-4.1355638
C	2.7466733	-1.0601469	-4.0032733
C	3.1141621	0.2554054	-3.6547668
C	4.5190602	0.3111922	-3.5694443
H	5.1091525	1.1723926	-3.3063299
H	2.4330018	1.0566919	-3.4221335
H	1.7385665	-1.4286118	-4.0832763
H	3.9776430	-2.8753935	-4.3926466
H	6.0568909	-1.2512245	-3.9256181
C	4.4900680	-1.0919307	0.3555353
C	4.2075389	-2.4356658	0.0439045
C	5.1085800	-2.8596763	-0.9556305
C	5.9629655	-1.7691656	-1.2436434
C	5.5676413	-0.6690881	-0.4467641
H	6.0116682	0.3115959	-0.4511829
H	6.7743661	-1.7746762	-1.9472835
H	5.1449293	-3.8367276	-1.4082472
H	3.3939917	-3.0129051	0.4453448

H	3.9344432	−0.4756708	1.0399424

*(Cp2TiCl)2(THF)*L2 (Fig. 12; Fig. S21, entry 2):*

101

H	0.6425906	0.9848294	−0.2216549
N	0.0789958	1.7949566	0.0713549
C	−1.2469791	1.6808536	−0.0282949
C	−2.1100201	0.5653777	−0.1356102
N	−2.0015488	−0.7661184	−0.0730058
C	−1.1244677	−1.5145105	0.7051831
C	−0.4231387	−0.9490103	1.7675029
C	0.3837445	−1.7524345	2.5572984
C	0.4980845	−3.1151856	2.3220185
C	−0.2207712	−3.6663387	1.2674313
C	−1.0261361	−2.8834090	0.4575817
H	−1.5797168	−3.3198863	−0.3593063
C	−0.1982994	−5.1532796	1.0664476
F	0.9799519	−5.7125567	1.4391111
F	−1.1656020	−5.7680133	1.8033758
F	−0.4116418	−5.5131257	−0.2232112
H	1.1258020	−3.7323882	2.9426604
C	1.0403013	−1.1632895	3.7721330
F	2.2177601	−1.7595266	4.0717575
F	1.2879008	0.1628165	3.6447918
F	0.2473663	−1.3017494	4.8735731
H	−0.5283746	0.0986388	1.9890585
H	−2.7569274	−1.2723252	−0.5560400
C	−3.2578199	1.4067834	−0.4465323
O	−4.4533533	1.2501955	−0.7400412
Ti	−5.9721022	−0.1932428	−0.6383100
Cl	−4.3953569	−1.7529010	−1.8130479
C	−5.4830928	−1.8211293	1.0968035
C	−4.9891090	−0.5747135	1.5149332
C	−6.0828816	0.3143253	1.6512567
C	−7.2504212	−0.4007343	1.3220421
C	−6.8778623	−1.7168639	0.9532543
H	−7.5416728	−2.4957678	0.6175643
H	−8.2511642	−0.0089649	1.3269274
H	−6.0288885	1.3510779	1.9391509
H	−3.9538821	−0.3318559	1.6728035
H	−4.8917001	−2.6857435	0.8543694
C	−6.4844813	0.4460853	−2.8501391
C	−7.3214516	−0.6445922	−2.5089223
C	−8.1904492	−0.2151763	−1.4927949
C	−7.8937982	1.1401238	−1.2018217
C	−6.8596905	1.5483261	−2.0558067
H	−6.3699037	2.5063230	−2.0368485
H	−8.3632441	1.7439693	−0.4438185
H	−8.9313123	−0.8185688	−0.9980097
H	−7.2678511	−1.6365818	−2.9223860
H	−5.6749798	0.4221236	−3.5582663
C	−2.3758679	2.6194358	−0.3164246
O	−2.5149493	3.8203100	−0.4271522
C	0.7712014	2.9947796	0.2558150

C	0.2148441	4.0383359	0.9923973
C	0.9243334	5.2164870	1.1433549
C	2.1881596	5.3752966	0.5879619
C	2.7359728	4.3199088	−0.1293134
C	2.0449534	3.1287522	−0.2945603
H	2.4628639	2.3130942	−0.8659345
C	4.1101308	4.4906970	−0.7080896
F	5.0758588	4.3881028	0.2488244
F	4.2743391	5.7033709	−1.2892366
F	4.4014098	3.5607152	−1.6489353
H	2.7293337	6.2998180	0.7089319
C	0.3239733	6.3706233	1.8949049
F	−0.0071918	7.3917793	1.0586301
F	1.1881809	6.8869096	2.8068512
F	−0.8016245	6.0370745	2.5652577
H	−0.7615087	3.9248577	1.4280444
Cl	1.6750690	−0.5407493	−1.2988630
Ti	4.1357645	−1.0513153	−1.1880620
C	3.6877727	−0.2264672	1.0431983
C	4.7499087	0.5193736	0.5044221
C	5.8325385	−0.3654682	0.2929640
C	5.4260224	−1.6601773	0.6709613
C	4.0917202	−1.5670857	1.1363365
H	3.4685300	−2.3853398	1.4506088
H	6.0304253	−2.5488333	0.6240945
H	6.7983282	−0.0993619	−0.0965615
H	4.7452152	1.5734669	0.2907704
H	2.7098679	0.1489518	1.2782996
C	4.3020439	0.3460199	−3.0649447
C	5.6038392	0.1949254	−2.5571586
C	5.9838477	−1.1622135	−2.7030870
C	4.9249695	−1.8366356	−3.3326347
C	3.8802769	−0.9183725	−3.5434205
H	2.9159899	−1.1363045	−3.9661457
H	4.8943612	−2.8894405	−3.5541461
H	6.9107097	−1.6014091	−2.3737617
H	6.1995333	0.9732827	−2.1148973
H	3.7212254	1.2512328	−3.0800873
O	3.4218093	−3.1522738	−1.2307436
C	2.2819780	−3.6324689	−1.9997880
C	2.6647232	−5.0211787	−2.4559688
H	1.7913018	−5.6469958	−2.6216327
C	3.5481621	−5.5125832	−1.3180377
H	4.2194992	−6.3156596	−1.6118071
C	4.3076560	−4.2634729	−0.9239530
H	4.5460933	−4.2214009	0.1326289
H	5.2230524	−4.1385932	−1.5014051
H	2.9394055	−5.8650833	−0.4873497
H	3.2304435	−4.9737532	−3.3860901
H	2.1020106	−2.9351067	−2.8073557
H	1.4152598	−3.6288481	−1.3424667
